# Common or distinct pathways to psychosis? A systematic review of evidence from prospective studies for developmental risk factors and antecedents of the schizophrenia spectrum disorders and affective psychoses

**DOI:** 10.1186/s12888-015-0562-2

**Published:** 2015-08-25

**Authors:** Kristin R. Laurens, Luming Luo, Sandra L. Matheson, Vaughan J. Carr, Alessandra Raudino, Felicity Harris, Melissa J. Green

**Affiliations:** 1 Research Unit for Schizophrenia Epidemiology, School of Psychiatry, University of New South Wales, Sydney, Australia; 2 Schizophrenia Research Institute, Sydney, Australia; 3 Department of Forensic and Neurodevelopmental Sciences, Institute of Psychiatry, Psychology & Neuroscience, King’s College London, London, UK; 4 Black Dog Institute, Prince of Wales Hospital, Sydney, Australia; 5 Department of Psychiatry, Monash University, Melbourne, Australia; 6 Neuroscience Research Australia, Sydney, Australia

## Abstract

**Background:**

Identifying the unique and shared premorbid indicators of risk for the schizophrenia spectrum disorders (SSD) and affective psychoses (AP) may refine aetiological hypotheses and inform the delivery of universal versus targeted preventive interventions. This systematic review synthesises the available evidence concerning developmental risk factors and antecedents of SSD and AP to identify those with the most robust support, and to highlight remaining evidence gaps.

**Methods:**

A systematic search of prospective birth, population, high-risk, and case-control cohorts was conducted in Medline and supplemented by hand searching, incorporating published studies in English with full text available. Inclusion/exclusion decisions and data extraction were completed in duplicate. Exposures included three categories of risk factors and four categories of antecedents, with case and comparison groups defined by adult psychiatric diagnosis. Effect sizes and prevalence rates were extracted, where available, and the strength of evidence synthesised and evaluated qualitatively across the study designs.

**Results:**

Of 1775 studies identified by the search, 127 provided data to the review. Individuals who develop SSD experience a diversity of subtle premorbid developmental deficits and risk exposures, spanning the prenatal period through early adolescence. Those of greatest magnitude (or observed most consistently) included obstetric complications, maternal illness during pregnancy (especially infections), other maternal physical factors, negative family emotional environment, psychopathology and psychotic symptoms, and cognitive and motor dysfunctions. Relatively less evidence has accumulated to implicate this diversity of exposures in AP, and many yet remain unexamined, with the most consistent or strongest evidence to date being for obstetric complications, psychopathology, cognitive indicators and motor dysfunction. Among the few investigations affording direct comparison between SSD and AP, larger effect sizes and a greater number of significant associations are commonly reported for SSD relative to AP.

**Conclusions:**

Shared risk factors for SSD and AP may include obstetric complications, childhood psychopathology, cognitive markers and motor dysfunction, but the capacity to distinguish common versus distinct risk factors/antecedents for SSD and AP is limited by the scant availability of prospective data for AP, and inconsistency in replication. Further studies considering both diagnoses concurrently are needed. Nonetheless, the prevalence of the risk factors/antecedents observed in cases and controls helps demarcate potential targets for preventative interventions for these disorders.

**Electronic supplementary material:**

The online version of this article (doi:10.1186/s12888-015-0562-2) contains supplementary material, which is available to authorized users.

## Background

Accumulating evidence has identified childhood and adolescent developmental risk factors and antecedents for schizophrenia spectrum disorders (SSD) and affective psychoses (AP), but the extent to which these factors may be common or unique to each disorder remains unclear. In the context of recent evidence of shared genetic vulnerability for schizophrenia and bipolar disorder [1–5], and potential similarities in their neuropathology [6, 7], distinguishing shared versus unique features of the developmental pathways to these disorders is important for determining their pathogenesis and the development of preventative interventions. Antecedents of a disorder may be expressed as premorbid deviations in brain development, evident in subtle deficits in functioning and delayed developmental milestones that likely reflect early expression of pathology, while risk factors include various social or physical environmental exposures that may represent modifiable targets for prevention [8, 9]. The term ‘risk factors’ is applied here to those indicators that may constitute relatively passive markers of increased risk, whereas ‘antecedents’ is used to demarcate factors that are putatively indicative of active risk-modifying mechanisms or processes through which the illness outcome may arise. Nonetheless, this distinction between risk factors and antecedents remains arbitrary with respect to our understanding of the developmental pathway to SSD and AP; this review focuses on summarising the evidence for shared and distinct developmental profiles for these disorders, but does not address the distinction between risk-factors and antecedents directly.

Previous evidence suggests that some risk factors and antecedents may represent generalised precursors to a range of disorders, while others may be specific to SSD or to AP. For instance, childhood emotional and interpersonal deviance is associated with both SSD and AP [9, 10], whereas premorbid impairment in motor, language, and cognitive functioning [11, 12], obstetric complications [13, 14] and various environmental exposures [8, 15], have been more commonly associated with SSD than AP. However, prior reviews have focused on synthesising evidence only for a single diagnostic outcome rather than comparing the disorders directly. Longitudinal studies *prospectively* assessing risk factors and antecedents for SSD and AP during childhood or early adolescence provide a particularly sound basis for delineating the developmental trajectories of each disorder, avoiding potential recall bias inherent in retrospective reporting [16]. These studies predominantly use population-based (including birth) cohorts or nested case-control designs. Whilst the latter allow for greater control over potential confounders, the large non-selected samples utilised in population-based cohorts provide greater precision in effect size estimates [17] and minimise selection bias [18]. Routinely-collected administrative datasets are increasingly used in large-scale population studies as they provide access to a wealth of prospectively gathered developmental data for low-prevalence psychiatric outcomes such as SSD and AP, particularly in relation to demographic and perinatal factors [19–21]. Other prospective investigations have followed the development of children with a family history of SSD or AP, typically the offspring of affected mothers, using a “high-risk” design that enriches the study sample with individuals who later develop the disorder in question (e.g. [22–24]). High-risk studies offer the potential to explore gene-environment interactions; however, as only a minority of people with SSD or AP have a first- or second-degree relative with these disorders [25, 26], findings from these studies may not generalise to the majority without a family history.

The objective of this systematic review was to summarise the available evidence on risk factors and antecedents of SSD and AP from cohorts providing prospectively gathered data, to identify common and distinct factors characterising the risk profiles for SSD and AP, ascertain the prevalence of these factors in both cases and comparison groups, and ultimately highlight potential targets for universal and targeted preventative interventions. Retrospectively gathered data was not included in order to reduce the risk of reporting bias that is inherent in the use of such data. Risk factors were reviewed within three main categories: (i) conception, pregnancy, and birth risk factors, (ii) demographic and familial risk factors, and (iii) childhood and adolescent risk factors. Antecedents were considered within four categories: (i) social, emotional, and behavioural functioning, and psychosis symptoms, (ii) cognitive functioning, (iii) language functioning, and (iv) motor functioning and developmental motor milestones. For each category, we firstly present the effect sizes specific to each for these groups of disorders, to identify factors supported by the most robust evidence. A meta-analytic approach to synthesising this evidence was precluded because too few factors had been measured consistently across studies. Secondly, we highlight where potential risk factors or antecedents have not yet been examined or are available for just one group of disorders, so that future research can be directed to fill these gaps in knowledge.

## Methods

This systematic review was designed and reported according to the guidance provided by the Preferred Reporting Items for Systematic Reviews and Meta-Analyses (PRISMA [27]; see Additional file 1).

### Search strategy

Search terms (exp schizophrenia, schizophreni*, exp bipolar disorder, bipolar.tw, exp affective psychosis, birth cohort, population cohort, longitudinal study, prospective study, high-risk; limited to full-text, English language publications) were applied to Medline in July 2014, and supplemented by extensive hand searching of citations and reference lists to identify additional studies. (‘exp’ and ‘.tw’ are Medline terms respectively signifying ‘explode’ and ‘text word’).

#### Study selection criteria

Studies were included if they satisfied the following criteria: (i) population or birth cohort studies, or case-control studies (including high-risk studies) where risk/antecedent measures were collected prospectively, (ii) measured antecedents and risk factors at mean age ≤15 years (in attempt to distinguish premorbid indicators from potential early symptoms/signs of illness [28]), (iii) diagnoses of SSD or AP obtained through standardised structured interview, hospital records or administrative registers, (iv) written in English, and (v) full text of the manuscript available. Studies excluded were those that relied on retrospective reports of exposures, did not provide a standardised or administrative psychiatric diagnosis, or examined indirect exposures (i.e., assessed only at the population rather than individual level). The decision to include or exclude studies was conducted in duplicate by two of the authors (LL and SLM).

#### Case and comparison groups

Case and comparison groups were defined by adult psychiatric diagnosis. Case groups comprised individuals diagnosed with SSDs (namely, schizophrenia, schizoaffective disorder, schizophreniform disorder, and other schizophrenia spectrum disorders) or APs (namely, bipolar disorder, mania, major depressive disorder with psychosis, and other affective psychoses). These groupings reflected the typical treatment of diagnoses within the primary studies included in the review – for example, a majority of studies included schizoaffective disorder as a SSD rather than AP. Where the primary study provided insufficient diagnostic information to be able to assign it to the SSD or AP outcome (e.g., ‘first-episode psychosis’), the study was excluded from the review. Three types of comparison groups were considered, as shown in the supplementary tables: (i) population controls (i.e., all those in the sample who did not have a SSD or AP), (ii) other (non-SSD or non-AP) psychiatric diagnoses only, and (iii) healthy controls only.

### Data extraction and analysis

Data extraction was conducted in duplicate by two of the authors (LL and SLM). Risk factors were reviewed within three main categories: (i) conception, pregnancy, and birth risk factors, (ii) demographic and familial risk factors, and (iii) childhood and adolescent risk factors. Antecedents were considered within four categories: (i) social, emotional, and behavioural functioning, and psychosis symptoms, (ii) cognitive functioning, (iii) language functioning, and (iv) motor functioning and developmental motor milestones. Each antecedent was further delineated by the age at assessment in order to characterise the developmental timing at which antecedents were apparent. Age was grouped according to those most commonly reported across studies, namely: (i) early childhood (0–5 years), (ii) middle childhood (6–12 years), and (iii) early adolescence (13–15 years). Where studies reported data that overlapped these age groups, the findings were allocated to the age bracket that incorporated the greater number of years (e.g., data reported in [29], spanning ages 3 to 9 years, were allocated to middle rather than early childhood).

Where available, adjusted effect sizes (odds ratios [ORs], risk ratios [RRs], hazard ratios [HRs], or incidence rate ratios [IRRs]) are reported as provided in the individual studies. For studies where adjusted effect sizes were not reported by the original manuscripts, estimated (unadjusted) ORs (calculated using proportions extracted from the primary studies) are presented. Where those proportions were not reported, we instead estimated the ORs from standardised mean differences (SMDs), F- or t-statistics, or regression coefficients (as per the Practical Meta-Analysis Effect Size Calculator: http://www.campbellcollaboration.org/resources/effect_size_input.php). As the majority of outcome data were dichotomous in nature, these effect sizes were estimated using ORs. For those studies that provided data permitting extraction of the prevalence rates of each risk factor/antecedent for each diagnostic group, these are also incorporated in the tables, indicating how common each risk factor/antecedent is in cases and controls.

To synthesise and evaluate qualitatively the strength of evidence available, and for ease of interpretation, the evidence from each study for risk factors and antecedents is summarized in Tables 1 and 2 using symbols that code: (i) study design (birth cohort, population cohort, high-risk cohort, or other non-high-risk case-control control); (ii) the largest reported or estimated significant effect size determined for each factor/antecedent, with grouping guided by the criteria of Rosenthal [30] and GRADEPro 2008 [31] and designated as large (OR/HR/RR/IRR: >5 [or <0.2 for reduced risk]), medium (OR/HR/RR/IRR: 2–5 [or 0.2–0.5]), small (OR/HR/RR/IRR: <2 [or >0.5]), or no statistically significant effect; and (iii) whether the effect is adjusted or unadjusted. Where both adjusted and unadjusted effects sizes were reported, only the adjusted data are presented in the Tables.

**Table 1:**
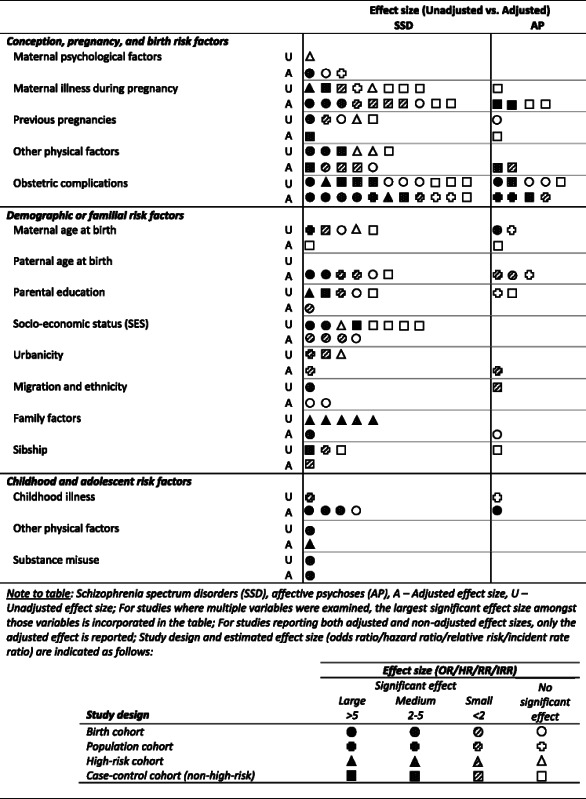
Evidence summary (by estimated magnitude of effect) of factors that increase risk of SSD and AP

**Table 2:**
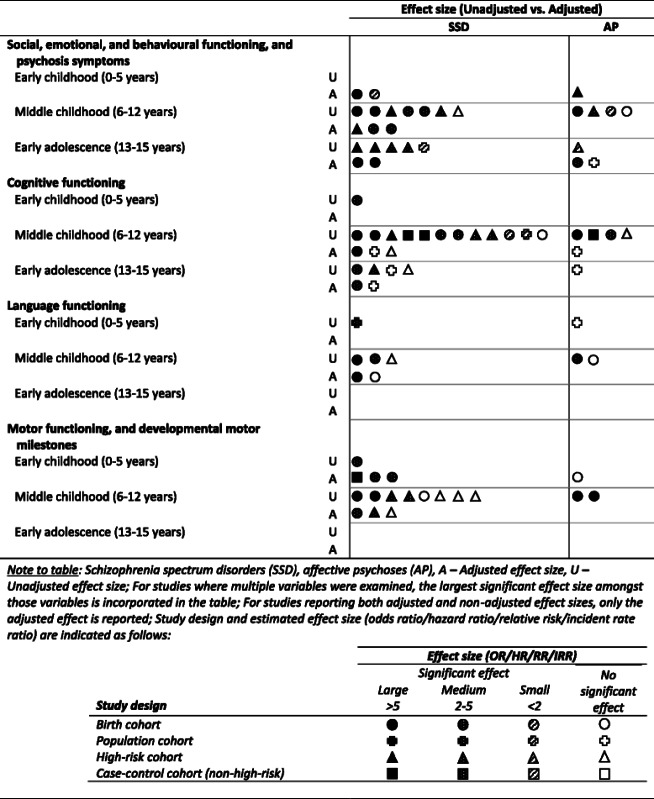
Evidence summary (by estimated magnitude of effect) of childhood antecedents that increase risk of SSD and AP

To reduce the risk of reporting bias, only data from prospective studies were synthesised and evaluated. While a formal quality assessment of the evidence from individual studies (including risk of bias) was beyond the scope of this review, the population/birth cohort studies typically provided greater precision in the effect estimates and less selection bias relative to the high-risk and case-control investigations. Thus, for each risk factor or antecedent category considered, we indicate in the text the number of cohorts of different design providing data, then present the evidence derived from cohorts providing data for both SSD and AP (permitting direct comparison), and additional evidence available from cohorts reporting solely on SSD or AP outcomes.

## Results

**Fig. 1:**
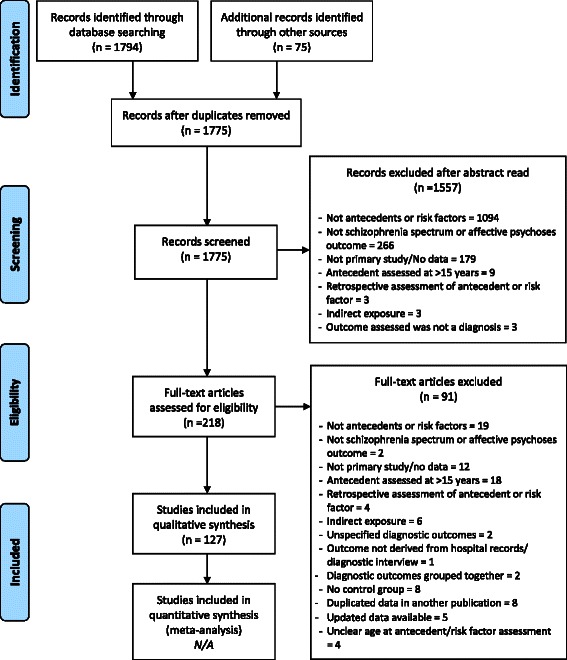
PRISMA diagram summarising the flow of information through the phases of systematic review

Figure 1 presents a PRISMA flow chart summary of the search and review process, including reasons for study exclusion. The Medline search identified 1794 potential studies, with a further 75 studies identified through hand-searching. A total of 127 studies met inclusion criteria.

Tables 1 and 2 present the evidence for risk factors and antecedents, respectively; and, for each risk factor/antecedent category, the design of the cohorts and a synthesis of the findings reported by each study. Additional file 2: Tables S1-S4 provide detailed information from each study (reported by study citation) including sample size, diagnosis, age at outcome assessment (diagnosis), age at exposure assessment (risk factor/antecedent), risk factor/antecedent measure, prevalence rates for the outcome, and effect sizes for cases relative to comparison group. Additional file 3: Table S5 provides further details regarding the study cohort and design for each citation, measurements used for diagnostic outcome and exposure variables and, where applicable, any confounding variables adjusted in the analyses.

### Conception, pregnancy, and birth risk factors (Table 1; Additional file 2: Table S1)

#### Maternal psychological factors during pregnancy

Limited data regarding the effects of maternal psychological factors during pregnancy on risk for later SSD in the offspring were available from two birth cohorts [32, 33], one population cohort [34], and one high-risk cohort [35]; no comparable studies were available for AP. Maternal psychological factors did not confer a significant increase in risk for SSD, with the exception of a medium-sized effect for depressed mood during pregnancy, *only* when either parent had a psychotic disorder [33]. Further research on the impact of maternal psychological factors during pregnancy is needed for both SSD and AP, although their influence may be limited.

#### Maternal illness during pregnancy

Evidence for the effects of maternal illness during pregnancy on SSD was available from three birth cohorts [36–39], two population cohorts [40, 41], two high-risk cohorts [42, 43], and six case-control cohorts [44–53]. Two of the latter [48, 50] also provided comparative data on AP, as did a Bain et al. ([54]; a companion study to the SSD investigation by Kendall et al. [52]). Another two case-control cohorts reported only on AP [55, 56]. The three case-control cohorts providing data on both SSD and AP showed no significant increase in risk for either diagnosis from hypertension and diabetes in pregnancy [50], or from pre-existing maternal physical illness [52, 54]. There was a significant effect for maternal infection and hypertension on SSD in one of the high-risk cohorts [42]. Similarly, herpes simplex virus infection (HSV-2) conferred a significantly increased risk for SSD, but not AP, in one of the case-control studies [48]. Among the studies examining only SSD, a significantly increased risk was identified for in-utero exposure to infectious diseases, including prenatal exposure to maternal genital and reproductive infections around the time of conception [36], gonococcal infection [39], HSV-2 [53], (but see [47]), respiratory infection in the second trimester [37], prenatal upper respiratory bacterial infection (not pneumonia [39]), influenza in the first trimester or first half of pregnancy [44], any treated maternal infection [41], and any physical illness during pregnancy [43] (but see [40, 42, 51, 52]), as well as any bacterial infection in the first trimester [39]. Compared with controls, increased risk of SSD among offspring was indexed also by markers of inflammation, including elevated maternal interleukin-8 levels [45] and C-reactive protein [49], but not maternal antibodies to toxoplasmosis [46]. For AP, two studies from a single case-control cohort provided evidence that maternal influenza was associated with an increased risk for AP [55, 56]. Thus, considerable evidence, mostly of small- or medium-sized effect (two cohorts reported large effects [36, 43]), suggests increased risk of SSD from prenatal exposure to maternal illness, especially infectious diseases. In contrast, the relative lack of studies examining AP precludes definitive conclusions, with current findings limited to the large effect for maternal influenza in two studies based on a single case-control cohort [49, 56].

#### Previous pregnancies

Two birth cohorts [57, 58], a population cohort [59], a high-risk cohort [42], and two case-control cohorts [49, 50] investigated the effects of previous pregnancy history on SSD or AP, with three studies reporting data for both outcomes [50, 58, 59]. Nosarti et al. [59] indicated an increased risk of SSD in offspring of mothers with parity of 1 and of ≥4, but a decreased risk in offspring of mothers with parity of 2–3 (but see [49] for lack of significant finding for parity ≥2); conversely, a reduced risk for AP was observed among offspring of mothers with parity of 2–3, and of ≥4 (but not for parity of 1). Sacker et al. [58] reported increased risk only for SSD in offspring of mothers with two prior pregnancies. No significant effects for grand multiparity (6+), or for prior miscarriages and abortions were observed for SSD, factors that remain untested in AP [42, 57]. Where observed, significant effect sizes were small or medium in magnitude.

#### Other physical factors

Data on the risk of SSD in offspring that is conferred by maternal physical factors during pregnancy (other than those relating to illness or parity) were reported in studies drawn from three birth cohorts [45, 60–64], two high-risk cohorts [65, 66], and three case-control cohorts [50, 51, 67]. Hultman et al. [50] examined both SSD and AP outcomes, while another case-control cohort investigated the AP outcome only [67]. In the only cohort examining both outcomes, an increased risk for both SSD and AP was conferred by winter birth [50], although data from two smaller high-risk cohorts indicated no effect of winter birth on SSD [65, 66]. Compared with controls, increased risk of SSD was reported among cases exposed to maternal docosahexaenoic acid [61] and low maternal retinol during the second trimester [60]. SSD offspring were more likely to be born to mothers with high pre-pregnancy body mass index (BMI ≥30; [63, 64]) or elevated BMI during pregnancy [51]. Decreased risk of SSD in male offspring of mothers receiving ≥2000 IU/day vitamin D (self-reported intake) was also reported [62]; conversely, a subsequent case-control study utilising bio-banked blood samples demonstrated increased risk of SSD conferred by both low and high neonatal vitamin D [68]. An increased risk for AP in offspring exposed to maternal smoking [67] contrasted with a lack of evidence for a maternal smoking effect in SSD [38, 45]. The significant effects observed for SSD and AP were typically small to medium in magnitude.

#### Obstetric complications

There is more extensive evidence concerning risk conferred by obstetric complications for SSD than AP. Seven birth cohorts [13, 38, 58, 63, 69–72], four population cohorts [59, 73–75], two high-risk cohorts [42, 76], and ten case-control cohorts [50–52, 77–83] provided data on SSD. Of these, three birth cohorts [29, 58, 72], three population cohorts [59, 73, 74], and two case-control cohorts [50, 54] also provided data concerning effects on AP. An additional case-control study provided data solely on AP [67].

Among those cohorts providing data for both outcomes, increased risk for both disorders was associated with premature birth [59, 74] and bleeding during pregnancy [58] (but see [50] indicating a significant effect for SSD only). Risk factors with significant effects on SSD, but not AP, included a high obstetric complications index score [13], fewer than 10 antenatal visits [58], and hypoxia-related complications [72]. Conversely, abnormal presentation of the foetus [52, 54] and non-spontaneous delivery [58] were associated with an increased risk for AP, but not SSD, as was small for gestational age ([73]; but see [29] for evidence that this factor also increases risk for SSD). All significant effect sizes for these risk factors ranged from small to large.

While considerable evidence implicates obstetric complications *collectively* as a risk factor contributing to both SSD and AP, there is limited evidence for any individual type of obstetric complication conferring risk for SSD or AP. Nonetheless, the following specific abnormalities are implicated in increasing risk for SSD (typically not yet examined on an individual basis for AP): foetal growth indicators (e.g., small for gestational age [29], small head circumference [77], low and high birth weight [38, 70], and short birth length [70]); potential hypoxia-related factors [29] (including low Apgar score [29, 38], placental abnormality [42], placental abruption [38], umbilical cord knotting [80], and atypical foetal presentation [80, 84]); and obstetric complication composite scores [50, 82]. However, non-significant effects for many of these risk factors were also reported for SSD [42, 63, 69, 78, 83, 84] and AP [67]. Unusually, in one case-control cohort, significant reductions in risk for SSD were associated with exposure to abdominal/pelvic X-ray, pre-eclampsia and maternal admission to hospital [52], but these effects were not observed for AP [54].

#### Summary of conception, pregnancy, and birth risk factors

A greater quantity of research has been undertaken for SSD than AP regarding the effects of exposure to conception, pregnancy, and birth factors, with the consequence that much evidence implicates these factors in SSD. Nonetheless, of all the risk factors summarised in Table 1 for AP, the greatest amount of evidence also implicates these factors. Effect sizes in most instances are small or medium for both SSD and AP (although several large effects were associated with some obstetric complications), and negative findings are almost as commonly observed as positive. There is often a lack of replication for individual risk factors across studies (in some cases attributable to heterogeneity in the categorisation of continuous variables such as birth weight and gestational age). The number of studies presenting data on both SSD and AP is limited.

### Demographic and familial risk factors (Table 1; Additional file 2: Table S2)

#### Maternal age at birth

Estimates of the effect of maternal age at birth on risk for SSD were available from one birth cohort [58], one population cohort [59], one high-risk cohort [43], and two case-control cohorts [45, 85]. The birth and population cohorts [58, 59], and a case-control cohort [50] also provided data for AP. Among studies showing a significant effect are those demonstrating increased risk for SSD in offspring whose mothers were younger at birth (<30 years [85] and <19 years [59]), while an inconsistent pattern of evidence relates to older mothers, including both increased risk of SSD in offspring of mothers ≥40 years in the population cohort [59], but decreased risk in offspring of mothers >30 years in a case-control cohort [85]. These effects are small to medium in magnitude. Among the three studies also reporting on AP, there is a lack of any effect of maternal age, with the exception of the birth cohort that showed a medium-sized increase in risk for mothers aged >34 years [58]. Thus, for both disorders, the state of evidence concerning this risk factor is inconclusive.

#### Paternal age at birth

Evidence concerning the effect of paternal age at birth on risk of SSD among offspring was available as adjusted effect sizes reported from three birth cohorts [86–88], two population cohorts [73, 89], and one case-control (sibling) cohort [85]. The population and case-control cohorts also provided data relating to AP, and one birth cohort provided data only for AP [90]. A more consistent pattern of findings emerged regarding paternal age at birth relative to that for maternal age, with increased risk for SSD observed when paternal age exceeded 30 [73, 88] or 34 years [89]. These effects were of small to medium magnitude (the latter especially for the more advanced paternal ages – see Additional file 2: Table S2), with large effects for paternal age ≥35 years observed among offspring who also had family history of SSD [87]. Of the two population cohorts providing evidence relating to AP, one showed increased risk associated with older paternal age (>30 years; small effect [73]), which was consistent with evidence from the birth cohort (35–44 years [90]). Thus, a pattern of increased risk for both SSD and AP appears to be conferred by advanced paternal age at birth, with no obvious effect relating to younger ages for either disorder.

#### Parental education

Data on the effects of parental educational attainment on SSD were available from one birth cohort [63, 91], one population cohort [59], one high-risk cohort [43], and two case-control cohorts [45, 49]. The population cohort provided concurrent data relating to AP, with information on AP also available from an additional case-control cohort [67]. With the exception of the birth cohort [91], the data pertained only to maternal education. Evidence for small and medium-sized effects of lower maternal education levels on increased risk for SSD was identified in the population cohort [59], the birth cohort [91] (although note the lack of evidence within a subset of this cohort [63]), and one of the case-control cohorts [45]. The birth cohort demonstrated a similar effect for lower paternal educational attainment [91]. In contrast, the high-risk cohort showed evidence for a large effect of higher maternal education for SSD, but this is in the context of a maternal history of schizophrenia [43]. Neither the population [59] nor case-control cohorts [67] reporting on AP found any effect for maternal education. Thus, the evidence suggests a replicated effect of lower maternal educational attainment on SSD, with non-significant findings in relation to AP.

#### Socio-economic status

Data regarding the effect of socio-economic status (SES; usually indexed by paternal occupation) on risk for SSD was provided by four birth cohorts [29, 91–95], one high-risk cohort [65], and six case-control studies [51, 96–100]. No published studies on SES were available for AP. All birth cohorts reported a significant effect for low SES (family, paternal, maternal, or area SES indicator) on increased risk of SSD. With the exception of one finding of a large effect [29], these effects were of small magnitude [91, 92, 95]. One birth cohort also identified a significant increase, of medium magnitude, in risk of SSD among individuals with the highest SES [94]. No significant effects were observed in the high-risk cohort, where only a small sample was available. Among the case-control cohorts, only one reported a significant effect, of large magnitude, for a low SES index (combining parental SES and area SES indicators). Against these consistent results, a single, case-control study indicated a small effect of decreased risk of SSD for individuals of low SES relative to high [97]. Comparative data for AP are needed.

#### Urbanicity

Effects of urbanicity on both SSD and AP were examined using data from two population cohorts [73, 101], with additional data available for SSD only from one high-risk cohort [66] and a case-control cohort [49]. All except the high-risk cohort reported a significant increase in risk for SSD, of small effect size, conferred by urban birth. In contrast, the evidence available from the population cohorts relating to AP was contradictory, with Laursen et al. [73] demonstrating an elevated risk for both SSD and AP conferred by urban birth, whereas Marcelis et al. [101] reported a decreased risk for AP associated with urban birth. Arguably, more weight might be assigned to findings of the first study, given its adjustment for a more extensive range of potential confounds. Nonetheless, further evidence relating to AP is needed to draw definitive conclusions regarding the relative effects of urban birth on risk for SSD and AP.

#### Migration and ethnicity

Prior meta-analyses support an effect of migrant/ethnic status on risk for SSD (e.g., [102]). Here we sought primary data that permitted calculation of the effects of migrant status on risk for SSD in studies where cases and controls were defined on the basis of psychiatric diagnosis. These data were available from one birth cohort [103], and two further birth cohorts [63, 104] also provided such data, but with maternal ethnicity instead of migrant status. Offspring with African-American (relative to white American) mothers experienced increased risk (medium-sized effect) for SSD in one birth cohort [63], but not the other [104]. More weight might be given to the latter finding due to its adjustment for potential confounds. Second-generation immigrant status was also not observed to confer a significant increase in risk of SSD [103]. For AP, data were provided by only one case-control cohort [67]; in contrast with SSD, risk of AP in this investigation was elevated among offspring of mothers with Caucasian ethnicity (small effect) but not offspring of African-American mothers or other maternal ethnicities. Thus, while rates of SSD appear elevated among migrant and ethnic minority groups when examined at a population level using meta-analysis [102], too few investigations met our study inclusion criteria to support any definitive conclusions concerning migrant and ethnic status on risk for SSD and AP.

#### Family factors

Data regarding the effects of family factors on risk for SSD were provided by one birth cohort [29] and three high-risk cohorts [65, 105–108]. The birth cohort [29] also provided concurrent data for AP. The birth cohort showed increased risk (medium effect) for SSD, but not AP, conferred by atypical mother-child interactions. Five studies, based on the three high-risk cohorts, consistently demonstrated increased risk for SSD in association with poor child-parent relationships (including “unsatisfactory” or “poor” relationships with a parent, family instability, paternal or maternal conflict, communication deviance, and negative affective style), with medium to large effect sizes [65, 105–107]. Further evidence from a high-risk study also indicated that paternal (but not maternal) absence, or institutional care during early childhood, increased risk for SSD (large effect) [108]. Although strong and apparently robust, findings of increased risk for SSD conferred by dysfunctional family factors derive predominantly from high-risk cohorts, and may have limited generalizability to the population. Further studies of these factors in relation to AP are needed.

#### Sibship

Data on the effects of sibship and birth order patterns on SSD were available from one population cohort [109] and four case-control cohorts [49–51, 85]. One of the case-control cohorts also provided data regarding AP [50]; for neither disorder was there evidence of an effect of twin birth on risk, whereas in another case-control cohort a decreased risk of SSD in twins was found [49]. Being first born [51, 85], having greater than three siblings [85], and a short interval between births [109] were each associated with small- or medium-sized increases in risk for SSD.

#### Summary of demographic and familial risk factors

Almost all evidence pertaining to the effect of demographic risk factors was available only for SSD, and limitations included sparse replication and diverse categorisation of variables across studies. The most consistent evidence suggested increased risk for SSD, and possibly AP, conferred by greater paternal age at birth, and increased risk of SSD among offspring who experienced poor parental relationships as children, although evidence for the latter drew predominantly from high-risk cohorts. The need for further investigation of the influence of demographic and familial factors on risk for AP is striking.

### Childhood and adolescent risk factors (Table 1; Additional file 2: Table S3)

#### Childhood illness

Data regarding the effect of childhood illness on SSD were provided by three birth cohorts [93, 110–112] and one population cohort [113], with two of the birth cohorts also providing concurrent data relating to risk for AP [111, 113]. Two of the birth cohorts provided evidence of increased risk of SSD associated with central nervous system infections ([112], medium effect; but see lack of significant effects in [93, 110]), and meningitis and tuberculosis ([111], a large effect); the latter effects were also present for AP (similarly, of large magnitude). Perinatal brain damage was associated with later SSD [93], as was head injury experienced between the ages of 11–15 years [113], but there was no effect of head injury in the comparative data relating to AP. Thus, relatively limited evidence suggests childhood infections and brain damage confer increased risk for SSD, with infections also contributing to risk of AP.

#### Other physical risk factors

One birth cohort [63] and one high-risk cohort [114] provided the only evidence available concerning the effect of physical growth factors during childhood on SSD. No corresponding evidence was available for AP. Shorter height during early childhood [114], and the combination of a low ponderal index at birth with high BMI at age 7 years [63] was associated with an increased risk for SSD (effects of medium and large magnitude, respectively).

#### Substance misuse

Two birth cohorts [115, 116] provide the only evidence regarding the effects of substance misuse on risk for SSD, with both reporting a significant association, of medium effect, between cannabis use during early adolescence and heightened risk for SSD. No comparative evidence was available regarding effects of cannabis or other substance misuse on risk for AP.

#### Summary of child and adolescent risk factors

Relatively limited evidence is available (particularly with respect to AP) concerning child and adolescent risk factors. Nonetheless, the effects of shorter height and cannabis use during early adolescence on risk for SSD were observed in data from at least two cohorts, and the significant effects observed for childhood infectious illnesses on risk for both SSD and AP reinforces the need for further research comparing the effects of these factors on both disorders.

### Childhood and adolescent antecedents (Table 2; Additional file 2: Table S4)

#### Social, emotional, and behavioural functioning and psychosis symptoms

##### Early childhood (0–5 years)

Limited early childhood evidence from three cohorts (two birth [116, 117], one high-risk [118]) supports an effect of patterns of childhood psychological functioning on risk for SSD and AP. The birth cohorts suggest that deviant behaviours [117], and childhood psychopathology in males (but not females, [116]), significantly increases risk for SSD, with small and large effect sizes, respectively. A small high-risk study [118] provided evidence for a large effect of elevated scores on the ‘Attention Problems’, ‘Aggressive Behaviour’, and ‘Anxious/Depressed’ subscales of the Child Behavior Checklist [119] on risk for AP. Conclusions regarding diagnostic specificity are limited because no studies directly compared SSD and AP outcomes. While the limited evidence to date suggests early childhood psychopathology may be implicated in both, further studies utilising a variety of study designs are needed.

##### Middle childhood (6–12 years)

Four birth cohort studies [29, 117, 120–124], and three high-risk studies [22, 23, 125, 126] provide evidence relating to the effect of middle childhood psychopathology on risk for SSD. Two of the birth cohorts [29, 120, 121, 124] provide concurrent information regarding AP as well as SSD, and one high-risk cohort [127] examined AP only. Evidence from the two birth cohorts examining both diagnoses concurrently indicated that both SSD and AP are preceded by social, emotional, and behavioural problems, although there was variability in the relative strength of the effect sizes observed across cohorts, antecedent measurement, and age of antecedent assessment. The effects observed for AP were less consistent than for SSD [29, 120, 121]. One birth cohort demonstrated evidence for medium/large effects of childhood psychosis symptoms on risk for SSD [123, 124], but not for AP [124]. These symptoms remained predictive for SSD up to age 38 years [123]. Further evidence from both birth cohort and high-risk studies examining only SSD implicates deviant behaviour, social maladjustment, emotional instability, thought disorder, and negative symptoms in conferring risk for SSD [22, 23, 117, 122, 125], with most of these effects of medium or large magnitude. From the high-risk cohort examining AP only, evidence of childhood behavioural and attention problems was found among those who later developed AP compared with healthy controls [127].

##### Early adolescence (13–15 years)

Evidence regarding the impact of early adolescent psychopathology on risk for SSD was provided by two birth cohorts [116, 128], one population cohort [129], and three high-risk cohorts [24, 65, 76, 130]. One of the birth cohorts additionally provided evidence regarding AP [29]; and further evidence relating to AP only was provided by one population cohort [131] and one high-risk cohort [132]. Evidence from the birth cohort permitting comparison of both outcomes indicated that increased risk for both SSD and AP was conferred by conduct/oppositional disorder and depression (medium/large effect sizes), while only increased SSD risk, not AP risk, was conferred by anxiety and attention-deficit/hyperactivity disorder (medium effect sizes) [128]. Within the high-risk cohorts examining only SSD, behavioural adjustment problems (poor family functioning, peer relationships, and school behaviour [130]) and deviant behaviour [24] increased risk of SSD with large effects, and disruptive behaviour increased this risk but with a medium effect size [65]. This was consistent with evidence of a reduction in risk for SSD conferred by the presence of behavioural competency (in conduct, orderliness, and motivation) in the population cohort [129], and a medium effect for self-reported overall psychopathology (among males only) in increasing SSD risk in another birth cohort [116]. The latter cohort further showed a medium-to-large effect of increased risk for SSD among male adolescents reporting psychotic-like thought problems, and a high-risk cohort provided evidence of small and medium effects respectively of increased risk for SSD among adolescents displaying paranoid and peculiar/eccentric behaviour [76]. In relation to AP, a small increase in risk was associated with depression in a high-risk cohort [132], with no increased risk associated with irritability in a small population cohort [131].

##### Summary of social, emotional, behavioural and psychosis-related antecedents

A relatively robust evidence base indicates increased risk for SSD in children presenting social, emotional, and behavioural problems and psychosis-related symptoms in childhood or adolescence. Less evidence is available regarding antecedents of AP, with preliminary evidence implicating social, emotional, and behavioural problems, but not psychosis-related symptoms.

#### Cognitive functioning

##### Early childhood (0–5 years)

Evidence available for this developmental period is limited to data from a single birth cohort [133] and relates to SSD only, indicating a medium effect size for poor cognitive functioning as an antecedent of SSD.

##### Middle childhood (6–12 years)

Five birth [29, 122, 133–137], two population [138, 139], three high-risk [23, 130, 140–142], and two nested case-control [96, 143] cohorts provide data pertaining to cognitive functioning in middle childhood as an antecedent of SSD. One of the birth cohorts [29, 135], one population cohort [139], and a case-control cohort [96] additionally provide data relating to AP, while a high-risk cohort provided evidence relating to AP only [140]. From the three cohorts providing both SSD and AP outcome data, the birth cohort provided consistent evidence (medium and large effects) of low IQ as an antecedent of SSD [29, 135], and mixed evidence of low IQ as an antecedent of AP (a medium effect noted for mania assessed at age 26 years [29], but no effect at age 32 years [135]). Conversely, the latter study indicated a large effect of high IQ as an antecedent of AP [135], although this finding should be interpreted with caution due to the unadjusted effect sizes and small size of the mania case group. The population cohort reported an inverse relationship between IQ and risk of SSD and of AP, but this was not significant for either disorder [139], whereas the nested case-control cohort indicated medium to large effects for lower IQ and several other cognitive impairments (particularly attention and working memory) as antecedents of SSD and AP, usually of slightly greater magnitude for SSD [96]. In the latter study, poorer academic achievement and perceptual motor ability showed medium effects as antecedents of SSD, but were not significantly related to AP outcome. The high-risk cohort providing evidence on AP only reported no significant effect of lower IQ on AP risk [140].

Consistent with the conclusions published in recent meta-analyses [11, 12, 144] implicating premorbid intelligence deficits as an antecedent of SSD, robust evidence of medium to large effects is available across birth, high-risk, and case-control cohorts in support of this association [29, 96, 133, 135–138, 141, 142]. Less data is available relating to academic achievement or other specific cognitive functions (e.g., attention, reading, vocabulary, etc.) as antecedents of SSD, with small [138], medium [96, 134], and large [96, 143], as well as non-significant [23], effects reported; the balance of evidence to date suggests a potentially broad range of premorbid cognitive impairments as antecedents of SSD.

##### Early adolescence (13–15 years)

Two birth cohorts [134, 145], two population cohorts [129, 146], and two high-risk cohorts [65, 147] examined cognitive functioning in early adolescence as an antecedent for SSD, with one population cohort also providing the only data relating to AP [146]. The latter study indicated no significant effect for lower verbal, spatial, or inductive abilities for either SSD or AP. For SSD, medium effects were observed for low IQ [147] (but no effect in [65]), for verbal, non-verbal, and arithmetic tests [134], and for below-age/special schooling level [145] (but no effect for academic achievement in [129]). The non-standardised nature of school-based cognitive measures may lack sensitivity to detect effects, while being in a special school or class may index marked impairments. The question of whether premorbid cognitive impairments become less prominent in early adolescence relative to those observed in middle childhood for SSD requires further longitudinal study, as does the question of whether cognitive dysfunctions in early adolescence constitute antecedents of AP.

##### Summary of cognitive functioning antecedents

There is robust evidence implicating poor cognitive functioning in middle childhood as an antecedent of SSD; suggestions that these deficits might emerge in early childhood and continue through early adolescence require further examination. Considerably less evidence is available regarding cognitive dysfunctions as antecedents of AP, with preliminary indications that both the low and high ends of the functional distribution might be associated with later AP.

#### Language functioning

##### Early childhood (0–5 years)

Evidence that developmental language disorder in early childhood confers a significantly increased risk for SSD (medium effect, based on unadjusted effect sizes) was available from a single population cohort [148], that reported no such effect for AP (although the latter was tested using only a small number of AP cases).

##### Middle childhood (6–12 years)

Data examining language functioning in middle childhood as an antecedent of SSD were provided by four birth cohorts [13, 111, 117, 134] and a high-risk cohort [149], with two of the birth cohorts also providing data regarding AP [13, 111]. Expressive language deficits conferred a significant increase in risk (medium effects) for both SSD and AP in one birth cohort [13], while speech problems were antecedent only to SSD and not AP in the other birth cohort [111]. Receptive language deficits had significant effects for SSD only (constituting a large but unadjusted effect size based on data spanning early-middle childhood years) [13]. Among the investigations reporting only on SSD, a large effect (based on adjusted effect sizes) was observed for abnormal speech, and significantly poorer expressive language and word association performance in one birth cohort, but no significant effects were reported for non-structural speech problems in a birth cohort [111] or for verbal associative disturbances in the high-risk cohort [149].

##### Early adolescence (13–15 years)

Our review identified no cohorts providing data on language functioning in early adolescence for either the SSD or AP outcomes.

##### Summary of language antecedents

Although several studies report no significant findings, problems with language expression appear to confer medium to large magnitude effect sizes for increased risk of both SSD and AP, whereas receptive language deficits and speech problems may be antecedent only to SSD. Further data relating to the development of AP are needed.

#### Motor functioning and developmental motor milestones

##### Early childhood (0–5 years)

Data from three birth cohorts [13, 150, 151] and a nested case-control study [78] provided evidence relating to motor dysfunctions or delayed motor milestone attainment during the early childhood years as an antecedent of SSD, with only one birth cohort providing concurrent data regarding AP [29]. In the latter, neurological abnormalities were related to SSD (medium effect) but not AP. A birth cohort and a case-control investigation examining SSD outcome only provided evidence of medium to large effects for delayed attainment of motor milestones (e.g., unsupported sitting, standing, and/or walking [78, 150]), and another birth cohort reported a medium effect for unusual movements/postural abnormalities [151].

##### Middle childhood (6–12 years)

Four birth cohorts [13, 111, 151, 152] and three high-risk cohorts [23, 126, 153–157] provided data to examine motor functioning in middle childhood as an antecedent for SSD, with two of the birth cohorts additionally providing data for AP [13, 111]. These two cohorts suggest that motor coordination and hand control problems may index risk for SSD, but not AP, although these medium-sized effects were estimated and unadjusted for potential confounding factors. Conversely, poor motor development and neurological problems conferred a significant risk (large effect sizes) for both disorders, with unsteadiness potentially also contributing to both outcomes. From a birth cohort and a high-risk cohort examining only SSD, significant increases in risk, of medium effect sizes, were found among children with minor physical anomalies [154], ocular alignment abnormalities [155], several indices of poor motor coordination [157], and unusual movements or postural abnormalities [151]. However, other high-risk cohorts demonstrate non-significant effects for neuromotor deficits as antecedent of SSD, including involuntary and abnormal movements [126], neurological soft signs [23], and gross motor skills [153], while both birth and high-risk cohorts show no significant associations between laterality (left- or mixed-hand preference) with SSD [111, 152, 156].

##### Early adolescence (13–15 years)

Our review identified no cohorts providing data on motor functioning in early adolescence for either SSD or AP.

##### Summary of motor antecedents

Delays in early childhood milestone attainment, and poor motor development and neurological problems in early and middle childhood, confer increased risk for later SSD, with similar dysfunctions in middle childhood also evident in individuals who later develop AP. Findings related to other motor skills or neurological soft signs are equivocal, and little evidence supports laterality disturbances in middle childhood as antecedent of SSD. Limited data, of large effect, suggests that poor motor development and neurological problems may also precede AP.

## Discussion

This systematic review aimed to elucidate common and distinct risk factors and antecedents characterising the developmental profiles of SSD and AP, which are groups of disorders that share both phenotypic and genetic features that suggest some overlap of aetiological mechanisms. The available evidence indicates that individuals who develop SSD experience diverse premorbid developmental deficits and risk exposures, spanning the prenatal period through early adolescence, although the effects are typically subtle. There is relatively less evidence supporting these risk exposures and patterns of premorbid functioning in AP, largely reflecting limited prospective investigation of this outcome. Few studies are available that afford direct comparison between the two groups of disorders within the same cohort. Amongst these studies, an overall trend emerges for larger effect sizes and a greater number of significant associations for SSD than AP. While this suggests some degree of specificity of many factors for SSD, a lack of available prospective data examining AP limits any conclusions regarding such specificity.

### Risk factors and antecedents for SSD

Amongst the risk factors examined in relation to SSD, the greatest amount of evidence was for obstetric complications, maternal infections during pregnancy, and other predominantly maternal physical factors, although for each of these categories almost half of the investigations reported non-significant effects. The positive evidence typically spanned the range of prospective study designs considered, and effect sizes ranged in magnitude from small to large. As the majority of effects were small to medium, the predictive sensitivity of any of these individual factors would be modest at best. All evidence regarding the influence of family factors on SSD was positive, and of medium or large effect, but derived almost exclusively from high-risk cohorts, and might not generalise to the population. For antecedents, the most consistently positive findings observed were premorbid deficits in cognitive functioning (particularly IQ) in the middle childhood period, and social, emotional, and behavioural problems, and psychosis symptoms in both middle childhood and in early adolescence. Effect sizes for these antecedents were predominantly of medium or large magnitude. A number of birth cohort and high-risk investigations also provided evidence of medium to large effects for premorbid motor dysfunctions in middle childhood, although almost half of studies reported no significant effects.

This review focuses on comparison of the risk factors and antecedents implicated in SSD and AP, rather than a detailed treatment of each specific factor and antecedent. For SSD in particular, a number of quality systematic reviews and meta-analyses are available for particular factors. A freely-available online resource, the Schizophrenia Library ([158, 159]; http://www.schizophreniaresearch.org.au/library/) collates the evidence from systematic reviews and meta-analyses (including those dealing with risk factors and antecedents of schizophrenia), grades the quality of the evidence available on each topic, and quantifies the magnitude of observed effects as small, medium, or large.

### Risk factors and antecedents for AP

The majority of evidence pertains to SSD, with no studies investigating AP in relation to maternal psychological factors during pregnancy, SES, childhood/adolescent physical risk factors, substance misuse, early childhood cognitive functioning, or early adolescent language and motor functioning. Many other factor/antecedent categories had a single cohort contributing data and often reported a non-significant effect. It is unclear whether this sparse evidence is partly attributable to publication bias, which would suggest negative findings for the majority of these factors in relation to AP. This possibility cannot be confirmed without studies that provide data for both SSD and AP. Among the relatively scant evidence from prospective investigations of AP, the most consistent and strongest evidence (mostly of medium to large effect) implicated obstetric complications in the development of these disorders, although some non-significant effects were also observed. This evidence derived from all study designs except high-risk cohorts. For all other risk factors, evidence was too sparse to draw definitive conclusions beyond the need for more prospective studies of these factors. Similarly, evidence relating to antecedents of AP was sparse, with the greatest amount and strongest of the limited evidence available for premorbid deficits in cognitive functioning, and in emotional and behavioural psychopathology in middle childhood. Large effects were observed in both of the birth cohorts providing data on motor dysfunctions in middle childhood. Again, data from prospective investigations of the many potential antecedents are needed (Table 2).

### Common or distinct pathways to SSD and AP?

Heterogeneity in the measurement of risk factors and antecedents for SSD and AP was likely to have contributed to a lack of consistently positive findings at an individual risk factor/antecedent level, but this might also suggest that the risks conferred by these factors are diagnostically non-specific in nature, or that existing literature does not provide sufficient direct comparisons of SSD and AP to distinguish their aetiological pathways. It is also important to note that the majority of effect sizes were estimated, with no adjusted effect sizes available for risk associated with several factors. For unadjusted data, reported effects may be attenuated when potential confounding factors, such as SES, are taken into account.

Similarly, for most antecedent categories, evidence from early childhood and early adolescence was sparse, particularly for language and motor functioning. While the strength of evidence relating to premorbid cognitive dysfunction was derived primarily from the middle childhood period, difficulties with social, emotional, and behavioural functioning spanned early childhood through adolescence. This may reflect different temporal trajectories for distinct antecedents, with specific deficits more likely to emerge at certain developmental stages; however, the paucity of longitudinal data on many of these factors makes it difficult to determine the extent to which age and/or developmental stage may moderate these effects.

It is now almost three decades since the publications from the original proponents of the neurodevelopmental hypothesis of schizophrenia [160, 161]. Their proposal, that a disruption of brain development during early life underlies the later emergence of psychosis during later adolescence or early adulthood, has become well established in the field, although the specific aetiological mechanisms operating in development of the illness remain to be determined. The neurodevelopmental origins of AP are relatively less well established in the literature, but in the context of the shared genetic vulnerability for schizophrenia and bipolar disorder [1–5], and the potential similarities in their neuropathology [6, 7], this review offers a timely indication of the need for further primary studies in this area, particularly studies that directly compare developmental risk factors and antecedents for SSD and AP. Multiple factors and antecedents implicated in SSD (and, to a lesser degree, AP) are identified within the present review; the next challenge is to integrate these findings into aetiological models that can stimulate the generation of new prevention and intervention trials. For example, a recent model draws together elements from the neurodevelopmental hypothesis with other major aetiological theories, namely dopamine and cognitive models of schizophrenia, to describe how early life events and the cognitions associated with them may act on an underlying biological vulnerability of dopamine dysregulation [162]. Accordingly, social and psychological interventions that reduce stress and alter cognitive schema are offered as potential means for modifying the mechanisms that dysregulate dopamine function. The present review identifies multiple factors and antecedents that might usefully be integrated into such models of illness aetiology to offer additional social, psychological, and cognitive targets for prevention and early intervention. Further, the review offers information regarding the specific childhood periods (early vs. middle childhood vs. early adolescence) in which such interventions might be targeted, according to risks emerging in these developmental periods.

### Implications of prevalence rates for preventative intervention

Substantial variation across studies was observed in the prevalence of risk factors and antecedents, which likely reflects differences in study design and measures used, methods of case selection and definition, and sample sizes. Prevalence rates drawn from population cohorts are likely to better reflect the true distribution of risk factors and antecedents in the population than those drawn from case-control/high-risk cohorts, where sampling bias may be operating. Greater confidence may be invested where consistency in rates across designs is observed.

Amongst factors for which consistent significant effects were reported, the prevalence of such factors may inform choice of approach for preventative interventions. Relatively high prevalence rates amongst cases were generally associated also with high prevalence in control groups, suggesting potential relevance for universal rather than targeted interventions. For example, poor family relationships (communication deviance, negative affective style, poor relationship with parents) were reported for between 46-80 % of SSD cases, compared with 16–29 % of healthy controls [106, 107]; although note that these rates derived from high-risk rather than birth/population cohorts. Premorbid psychotic symptoms at age 11 years were reported by almost half of those who later developed SSD (48 % of cases) compared with 13 % of healthy controls [124]. Other significant factors/antecedents of SSD for which moderate prevalence rates were reported included behavioural problems (31–38 % of cases versus 9–19 % of healthy controls [24, 116, 117]), low IQ (16–43 % of cases versus 3–26 % of healthy controls [133, 135, 137]), and cannabis use during early adolescence (12–32 % of cases versus 4–12 % of population controls [115, 116]). Less common antecedents, which might merit targeted interventions, include the Child Behavior Checklist-Bipolar Disorder phenotype as an antecedent to AP (present in 56 % of cases compared with 5 % in those who developed other disorders [118]), and motor coordination problems in relation to SSD (present in 11–14 % of those who developed SSD, compared with 3–4 % in healthy controls [111, 151]). Considering that the majority of consistently reported significant effects pertained to factors/antecedents that were relatively common in the general population, the identification of risk profiles based on algorithms combining multiple factors may increase predictive power for the purposes of targeted interventions [163].

### Limitations and future directions

This review had a broad remit, namely, to synthesise the available evidence concerning the variety of developmental risk factors and antecedents of SSD and AP examined to date that may be common or distinct to each disorder group, so as to determine those with the most robust support, and to highlight remaining evidence gaps requiring further research. To achieve this, the literature search and reporting was limited in a number of ways. These included the systematic search of only a single database (Medline) and the restriction to full-text publications written in English. We also restricted our search to include only prospective studies, in an attempt to mitigate against the potential bias of data obtained using retrospective reports. Regarding reporting, we chose to summarise the risk factors and antecedents, respectively, that *increased* risk of SSD and AP only (see Tables 1 and 2). A few studies [49, 52, 59, 81, 97, 101] reported data on various factors/antecedents for which a *decreased* risk of either outcome was identified, but were too isolated to permit any definitive conclusions regarding a potential protective effect conferred by such factors; these factors were therefore not incorporated in the Tables 1 and 2 summaries, but are noted in the text and Supplementary Tables. The mixed reporting of unadjusted and adjusted effect sizes was also necessary because the data from some studies permitted only the estimation of unadjusted effect sizes. Adjusted effect sizes are typically more useful; however, even amongst the adjusted effect sizes, inconsistencies arose due to the variability in confounders adjusted for. Moreover, depending on the confounders included in the model, adequate adjustment is not necessarily achieved. Given these issues, future reviews focused specifically on a particular factor or antecedent may overcome some of these limitations, and could incorporate retrospective studies to examine consistency of findings across the prospective and retrospective designs. Those reviews might delineate both risk and protective factors that may be operating at certain stages of development.

More permissive future reviews might also be able to examine a number of methodological factors that may contribute to heterogeneity of study findings but for which insufficient primary studies were available for consideration in the present review. Examples include diagnostic definitions employed (e.g., International Classification of Diseases [ICD] versus Diagnostic and Statistical Manual of Mental Disorders [DSM] criteria; Kraepelinian versus non-Kraepelinian courses of disease progression), sex differences, and age of onset of disorder. Here, the classification of disorders as SSD and AP was made according to their original designation in the included primary study. Search criteria were inclusive of all diagnoses in the SSD and AP groups, without specification of ICD or DSM criteria, which vary in detail such as the duration of symptoms required. Any diagnostic instability not accounted for in the original publications (e.g., where a non-affective first-episode psychosis presentation later evolves to AP) would likely bias the review toward identifying risk factors and antecedents that were common, rather than unique, to SSD and AP.

With respect to the translation of these epidemiological findings to new clinical or prevention recommendations, it is important to note that any evidence summarised here might be biased by residual confounding, as is inherent to observational studies. The covariates considered in each study are summarised in Additional file 3: Table S5, with variability apparent across studies. Although a number of investigations included parental history of psychoses as a covariate in their analyses, few investigations reported data indicating the effect of family history of psychoses. For instance, the birth cohort investigations reporting on depressed mood in pregnancy [33] and paternal age [87], respectively, indicated higher risk of SSD among individuals with a positive family history. Where findings from high-risk cohorts diverge from those obtained in birth or population cohort studies, this might suggest a potential effect of family history. Further primary studies that report these data are needed to examine how family history of SSD and/or AP may impact the findings and translational utility.

Finally, the capacity to identify common or distinct risk factors and antecedents operating in the aetiological pathways to SSD or AP within the current literature is limited not only by scant availability of prospective data for AP, but also by inconsistency in the effects observed. This is likely primarily due to differences in methodologies. Firstly, measures and thresholds used to define risk factors and antecedents differ greatly between studies. This is especially the case for exposures that are continuous in nature, where the change in risk associated with increasing exposure is unclear. Secondly, there is considerable variability in the sample sizes used across studies, especially for case groups where smaller numbers are likely underpowered to detect effects. This is especially pertinent within the limited literature relating to AP, which may bias findings regarding disorder specificity. Thirdly, inconsistencies or inadequacies of case definition may contribute to the dilution of observed effects, particularly for AP cases where many studies have not confirmed the presence of psychosis. Lastly, variability in age of assessment across studies makes informative comparisons difficult and limits the ability to investigate the effect of age/developmental stage as a moderator of observed effects. Most studies have used either data pooled across different age groups/stages at assessment, or have measured particular factors at one time point only, thus restricting the ability to ascertain developmental effects. Moreover, samples encompassing a broad age range at assessment may not well characterise the typical developmental profile during that period.

## Conclusions

This review highlights a striking gap in the literature regarding risk factors and antecedents for AP, and also highlights risk factors/antecedents of SSD that would benefit from further investigation. Whilst several risks associated with SSD are identified, it remains unclear whether these also characterise AP or are SSD-specific. Further, reliable comparisons from the evidence regarding specificity are hampered by lack of replication. Many of the factors investigated have previously been shown to increase risk for multiple disorders (e.g., [128, 164]), but AP is not often among those reported. This constitutes a significant limitation in the current evidence base in light of data from genetic studies underlining potential similarities in the aetiology of SSD and AP. Future research requires the investigation of AP and SSD concurrently to establish whether these similarities extend to common aetiological pathways for some individuals with these diagnoses. The low prevalence of both disorders calls for population-based approaches to provide the necessary power to detect effects, as well as to capture the full spectrum of premorbid exposures and developmental deviations that may characterise those who later develop either disorder. From an intervention perspective, applying a longitudinal framework to these investigations will further enhance scope to determine how early developmental deviations may be detected, or at which point during development they are most sensitive for indicating risk for later SSD or AP.

## Additional files

Additional file 1: **PRISMA.** (DOCX 24 kb)
Additional file 2: Table S1. Conception, pregnancy, and birth risk factors. **Table S2.** Demographic or familial risk factors. **Table S3.** Childhood and adolescent risk factors. **Table S4.** Childhood and adolescent antecedents. (DOCX 545 kb)
Additional file 3: Table S5. Study details (cohort/sample; study design; outcome measure; exposure measure; variables of adjustment). (DOCX 187 kb)

## References

[1] Craddock N, O'Donovan MC, Owen MJ (2006). Genes for schizophrenia and bipolar disorder? Implications for psychiatric nosology. Schizophr Bull.

[2] Lichtenstein P, Yip BH, Bjork C, Pawitan Y, Cannon TD, Sullivan PF, Hultman CM (2009). Common genetic determinants of schizophrenia and bipolar disorder in Swedish families: a population-based study. Lancet.

[3] Moskvina V, Craddock N, Holmans P, Nikolov I, Pahwa JS, Green E, Owen MJ, O'Donovan MC (2009). Gene-wide analyses of genome-wide association data sets: evidence for multiple common risk alleles for schizophrenia and bipolar disorder and for overlap in genetic risk. Mol Psychiatry.

[4] O'Donovan MC, Craddock N, Norton N, Williams H, Peirce T, Moskvina V, Nikolov I, Hamshere M, Carroll L, Georgieva L (2008). Identification of loci associated with schizophrenia by genome-wide association and follow-up. Nat Genet.

[5] Rasic D, Hajek T, Alda M, Uher R (2014). Risk of mental illness in offspring of parents with schizophrenia, bipolar disorder, and major depressive disorder: a meta-analysis of family high-risk studies. Schizophr Bull.

[6] Demjaha A, MacCabe JH, Murray RM (2012). How genes and environmental factors determine the different neurodevelopmental trajectories of schizophrenia and bipolar disorder. Schizophr Bull.

[7] Murray RM, Sham P, Van Os J, Zanelli J, Cannon M, McDonald C (2004). A developmental model for similarities and dissimilarities between schizophrenia and bipolar disorder. Schizophr Res.

[8] Matheson SL, Shepherd AM, Laurens KR, Carr VJ (2011). A systematic meta-review grading the evidence for non-genetic risk factors and putative antecedents of schizophrenia. Schizophr Res.

[9] Welham J, Isohanni M, Jones P, McGrath J (2009). The Antecedents of Schizophrenia: A Review of Birth Cohort Studies. Schizophr Bull.

[10] Tarbox SI, Pogue-Geile MF (2008). Development of social functioning in preschizophrenia children and adolescents: a systematic review. Psychol Bull.

[11] Dickson H, Laurens KR, Cullen AE, Hodgins S (2012). Meta-analyses of cognitive and motor function in youth aged 16 years and younger who subsequently develop schizophrenia. Psychol Med.

[12] Khandaker GM, Barnett JH, White IR, Jones PB (2011). A quantitative meta-analysis of population-based studies of premorbid intelligence and schizophrenia. Schizophr Res.

[13] Cannon M, Jones PB, Murray RM (2002). Obstetric complications and schizophrenia: Historical and meta-analytic review. Am J Psychiatr.

[14] Scott J, McNeill Y, Cavanagh J, Cannon M, Murray R (2006). Exposure to obstetric complications and subsequent development of bipolar disorder: Systematic review. Br J Psychiatry.

[15] Mortensen PB, Pedersen CB, Melbye M, Mors O, Ewald H (2003). Individual and familial risk factors for bipolar affective disorders in Denmark. Arch Gen Psychiatry.

[16] Maccabe JH (2008). Population-based cohort studies on premorbid cognitive function in schizophrenia. Epidemiol Rev.

[17] Austin PC, Anderson GM, Cigsar C, Gruneir A (2012). Comparing the cohort design and the nested case-control design in the presence of both time-invariant and time-dependent treatment and competing risks: bias and precision. Pharmacoepidemiol Drug Saf.

[18] Thompson L, Kemp J, Wilson P, Pritchett R, Minnis H, Toms-Whittle L, Puckering C, Law J, Gillberg C (2010). What have birth cohort studies asked about genetic, pre- and perinatal exposures and child and adolescent onset mental health outcomes? A systematic review. Eur Child Adolesc Psychiatry.

[19] Allebeck P (2009). The use of population based registers in psychiatric research. Acta Psychiatr Scand.

[20] Morgan VA, Jablensky AV (2010). From inventory to benchmark: quality of psychiatric case registers in research. Br J Psychiatry.

[21] Perera G, Soremekun M, Breen G, Stewart R (2009). The psychiatric case register: noble past, challenging present, but exciting future. Br J Psychiatr.

[22] Amminger GP, Pape S, Rock D, Roberts SA, Ott SL, Squires-Wheeler E, Kestenbaum C, Erlenmeyer-Kimling L (1999). Relationship between childhood behavioral disturbance and later schizophrenia in the New York High-Risk Project. Am J Psychiatr.

[23] Niemi LT, Suvisaari JM, Haukka JK, Lonnqvist JK (2005). Childhood predictors of future psychiatric morbidity in offspring of mothers with psychotic disorder: results from the Helsinki High-Risk Study. Br J Psychiatr.

[24] Olin SS, John RS, Mednick SA (1998). Assessing the predictive value of teacher reports in a high risk sample for schizophrenia: a ROC analysis. Schizophr Res.

[25] Gottesman II, Erlenmeyer-Kimling L (2001). Family and twin strategies as a head start in defining prodromes and endophenotypes for hypothetical early-interventions in schizophrenia. Schizophr Res.

[26] Joyce PR, Doughty CJ, Wells JE, Walsh AE, Admiraal A, Lill M, Olds RJ (2004). Affective disorders in the first-degree relatives of bipolar probands: results from the South Island Bipolar Study. Compr Psychiatry.

[27] Moher D, Liberati A, Tetzlaff J, Altman DG, Group P (2009). Preferred reporting items for systematic reviews and meta-analyses: the PRISMA statement. PLoS Med.

[28] Ruhrmann S, Schultze-Lutter F, Klosterkotter J (2010). Probably at-risk, but certainly ill--advocating the introduction of a psychosis spectrum disorder in DSM-V. Schizophr Res.

[29] Cannon M, Caspi A, Moffitt TE, Harrington H, Taylor A, Murray RM, Poulton R (2002). Evidence for early-childhood, pan-developmental impairment specific to schizophreniform disorder: Results from a longitudinal birth cohort. Arch Gen Psychiatry.

[30] Rosenthal JA (1996). Qualitative descriptors of strength of association and effect size. J Soc Serv Res.

[31] Brozek J, Oxman A, Schünemann H (2014). GRADEpro 2008. Version 3.2 for Windows.

[32] Herman DB, Brown AS, Opler MG, Desai M, Malaspina D, Bresnahan M, Schaefer CA, Susser ES (2006). Does unwantedness of pregnancy predict schizophrenia in the offspring? Findings from a prospective birth cohort study. Soc Psychiatry Psychiatr Epidemiol.

[33] Maki P, Riekki T, Miettunen J, Isohanni M, Jones PB, Murray GK, Veijola J (2010). Schizophrenia in the offspring of antenatally depressed mothers in the northern Finland 1966 birth cohort: relationship to family history of psychosis. Am J Psychiatr.

[34] Khashan AS, Abel KM, McNamee R (2008). Higher risk of offspring schizophrenia following antenatal maternal exposure to severe adverse life events. Arch Gen Psychiatry.

[35] Niemi LT, Suvisaari JM, Haukka JK, Lonnqvist JK (2004). Do maternal psychotic symptoms predict offspring's psychotic disorder? Findings from the Helsinki High-Risk Study. Psychiatry Res.

[36] Babulas V, Factor-Litvak P, Goetz R, Schaefer CA, Brown AS (2006). Prenatal exposure to maternal genital and reproductive infections and adult schizophrenia. Am J Psychiatr.

[37] Brown AS, Schaefer CA, Wyatt RJ, Goetz R, Begg MD, Gorman JM, Susser ES (2000). Maternal exposure to respiratory infections and adult schizophrenia spectrum disorders: a prospective birth cohort study. Schizophr Bull.

[38] Jones PB, Rantakallio P, Hartikainen AL, Isohanni M, Sipila P (1998). Schizophrenia as a long-term outcome of pregnancy, delivery, and perinatal complications: a 28-year follow-up of the 1966 north Finland general population birth cohort. Am J Psychiatr.

[39] Sørensen HJ, Mortensen EL, Reinisch JM, Mednick SA (2009). Association Between Prenatal Exposure to Bacterial Infection and Risk of Schizophrenia. Schizophr Bull.

[40] Clarke MC, Tanskanen A, Huttunen M, Whittaker JC, Cannon M (2009). Evidence for an Interaction Between Familial Liability and Prenatal Exposure to Infection in the Causation of Schizophrenia. Am J Psychiatr.

[41] Nielsen PR, Laursen TM, Mortensen PB (2013). Association Between Parental Hospital-Treated Infection and the Risk of Schizophrenia in Adolescence and Early Adulthood. Schizophr Bull.

[42] Suvisaari JM, Taxell-Lassas V, Pankakoski M, Haukka JK, Lönnqvist JK, Häkkinen LT (2012). Obstetric Complications as Risk Factors for Schizophrenia Spectrum Psychoses in Offspring of Mothers With Psychotic Disorder. Schizophr Bull.

[43] Talovic SA, Mednick SA, Schulsinger F, Falloon IR (1980). Schizophrenia in high-risk subjects: prognostic maternal characteristics. J Abnorm Psychol.

[44] Brown AS, Begg MD, Gravenstein S, Schaefer CA, Wyatt RJ, Bresnahan M, Babulas VP, Susser ES (2004). Serologic evidence of prenatal influenza in the etiology of schizophrenia. Arch Gen Psychiatry.

[45] Brown AS, Hooton J, Schaefer CA, Zhang H, Petkova E, Babulas V, Perrin M, Gorman JM, Susser ES (2004). Elevated maternal interleukin-8 levels and risk of schizophrenia in adult offspring. Am J Psychiatr.

[46] Brown AS, Schaefer CA, Quesenberry CP, Liu L, Babulas VP, Susser ES (2005). Maternal exposure to toxoplasmosis and risk of schizophrenia in adult offspring. Am J Psychiatr.

[47] Brown AS, Schaefer CA, Quesenberry CP, Shen L, Susser ES (2006). No evidence of relation between maternal exposure to herpes simplex virus type 2 and risk of schizophrenia?. Am J Psychiatr.

[48] Buka SL, Cannon TD, Torrey EF, Yolken RH (2008). Maternal Exposure to Herpes Simplex Virus and Risk of Psychosis Among Adult Offspring. Biol Psychiatry.

[49] Canetta S, Sourander A, Surcel HM, Hinkka-Yli-Salomaki S, Leiviska J, Kellendonk C, et al. Elevated Maternal C-Reactive Protein and Increased Risk of Schizophrenia in a National Birth Cohort. Am J Psychiatr. In press. 10.1176/appi.ajp.2014.13121579 PMC4159178 24969261

[50] Hultman CM, Sparen P, Takei N, Murray RM, Cnattingius S (1999). Prenatal and perinatal risk factors for schizophrenia, affective psychosis, and reactive psychosis of early onset: case-control study. Br Med J.

[51] Kawai M, Minabe Y, Takagai S, Ogai M, Matsumoto H, Mori N, Takei N (2004). Poor maternal care and high maternal body mass index in pregnancy as a risk factor for schizophrenia in offspring. Acta Psychiatr Scand.

[52] Kendell RE, McInneny K, Juszczak E, Bain M (2000). Obstetric complications and schizophrenia. Two case-control studies based on structured obstetric records. Br J Psychiatr.

[53] Mortensen PB, Pedersen CB, Hougaard DM, Nørgaard-Petersen B, Mors O, Børglum AD, Yolken RH (2010). A Danish National Birth Cohort study of maternal HSV-2 antibodies as a risk factor for schizophrenia in their offspring. Schizophr Res.

[54] Bain M, Juszczak E, McInneny K, Kendell RE (2000). Obstetric complications and affective psychoses. Two case-control studies based on structured obstetric records. BrJ Psychiatr.

[55] Canetta SE, Bao Y, Co MDT, Ennis FA, Cruz J, Terajima M, Shen L, Kellendonk C, Schaefer CA, Brown AS (2014). Serological documentation of maternal influenza exposure and bipolar disorder in adult offspring. Am J Psychiatr.

[56] Parboosing R, Bao Y, Shen L, Schaefer CA, Brown AS (2013). Gestational influenza and bipolar disorder in adult offspring. JAMA Psychiatry.

[57] Kemppainen L, Makikyro T, Jokelainen J, Nieminen P, Jarvelin MR, Isohanni M (2000). Is grand multiparity associated with offsprings' hospital-treated mental disorders? A 28-year follow-up of the North Finland 1966 birth cohort. Soc Psychiatry Psychiatr Epidemiol.

[58] Sacker A, Done DJ, Crow TJ, Golding J (1995). Antecedents of schizophrenia and affective illness. Obstetric complications. Br J Psychiatr.

[59] Nosarti C, Reichenberg A, Murray RM, Cnattingius S, Lambe MP, Yin L, MacCabe J, Rifkin L, Hultman CM (2012). Preterm birth and psychiatric disorders in young adult life. Arch Gen Psychiatry.

[60] Bao Y, Ibram G, Blaner WS, Quesenberry CP, Shen L, McKeague IW, Schaefer CA, Susser ES, Brown AS (2012). Low maternal retinol as a risk factor for schizophrenia in adult offspring. Schizophr Res.

[61] Harper KN, Hibbeln JR, Deckelbaum R, Quesenberry CP, Schaefer CA, Brown AS (2011). Maternal serum docosahexaenoic acid and schizophrenia spectrum disorders in adult offspring. Schizophr Res.

[62] McGrath J, Saari K, Hakko H, Jokelainen J, Jones P, Jarvelin M-R, Chant D, Isohanni M (2004). Vitamin D supplementation during the first year of life and risk of schizophrenia: a Finnish birth cohort study. Schizophr Res.

[63] Perrin MA, Chen H, Sandberg DE, Malaspina D, Brown AS (2007). Growth trajectory during early life and risk of adult schizophrenia. Br J Psychiatr.

[64] Schaefer CA, Brown AS, Wyatt RJ, Kline J, Begg MD, Bresnahan MA, Susser ES (2000). Maternal Prepregnant Body Mass and Risk of Schizophrenia in Adult Offspring. Schizophr Bull.

[65] Carter JW, Schulsinger F, Parnas J, Cannon T, Mednick SA (2003). A multivariate prediction model of schizophrenia. Schizophr Bull.

[66] Machon RA, Mednick SA, Schulsinger F (1987). Seasonality, birth complications and schizophrenia in a high risk sample. Br J Psychiatr.

[67] Talati A, Bao Y, Kaufman J, Shen L, Schaefer CA, Brown AS (2013). Maternal smoking during pregnancy and bipolar disorder in offspring. Am J Psychiatr.

[68] McGrath JJ, Eyles DW, Pedersen CB, Anderson C, Ko P, Burne TH, Norgaard-Pedersen B, Hougaard DM, Mortensen PB (2010). Neonatal vitamin D status and risk of schizophrenia: a population-based case-control study. Arch Gen Psychiatry.

[69] Hollister J, Laing P, Mednick SA (1996). Rhesus incompatibility as a risk factor for schizophrenia in male adults. Arch Gen Psychiatry.

[70] Moilanen K, Jokelainen J, Jones PB, Hartikainen A-L, Jarvelin M-R, Isohanni M (2010). Deviant intrauterine growth and risk of schizophrenia: a 34-year follow-up of the Northern Finland 1966 Birth Cohort. Schizophr Res.

[71] Tuovinen S, Räikkönen K, Pesonen A-K, Lahti M, Heinonen K, Wahlbeck K, Kajantie E, Osmond C, Barker DJP, Eriksson JG (2012). Hypertensive disorders in pregnancy and risk of severe mental disorders in the offspring in adulthood: The Helsinki Birth Cohort Study. J Psychiatr Res.

[72] Zornberg GL, Buka SL, Tsuang MT (2000). Hypoxic-ischemia-related fetal/neonatal complications and risk of schizophrenia and other nonaffective psychoses: a 19-year longitudinal study. Am J Psychiatr.

[73] Laursen TM, Munk-Olsen T, Nordentoft M, Bo Mortensen P (2007). A comparison of selected risk factors for unipolar depressive disorder, bipolar affective disorder, schizoaffective disorder, and schizophrenia from a danish population-based cohort. J Clin Psychiatr.

[74] Mathiasen R, Hansen BM, Forman JL, Kessing LV, Greisen G (2011). The risk of psychiatric disorders in individuals born prematurely in Denmark from 1974 to 1996. Acta Paediatr.

[75] Monfils GW, Josefsson A, Ekholm Selling K, Sydsjö G (2009). Preterm birth or foetal growth impairment and psychiatric hospitalization in adolescence and early adulthood in a Swedish population-based birth cohort. Acta Psychiatr Scand.

[76] Parnas J, Jorgensen A (1989). Pre-morbid psychopathology in schizophrenia spectrum. Br J Psychiatr.

[77] Cantor-Graae E, McNeil TF, Sjöström K, Nordström LG, Rosenlund T (1997). Maternal demographic correlates of increased history of obstetric complications in schizophrenia. J Psychiatr Res.

[78] Clarke MC, Tanskanen A, Huttunen M, Leon DA, Murray RM, Jones PB, Cannon M (2011). Increased risk of schizophrenia from additive interaction between infant motor developmental delay and obstetric complications: evidence from a population-based longitudinal study.[Erratum appears in Am J Psychiatry. 2011, 168(12):1345]. Am J Psychiatr.

[79] Freedman D, Bao Y, Kremen WS, Vinogradov S, McKeague IW, Brown AS (2013). Birth weight and neurocognition in schizophrenia spectrum disorders. Schizophr Bull.

[80] Gunther-Genta F, Bovet P, Hohlfeld P (1994). Obstetric complications and schizophrenia. A case-control study. Br J Psychiatr.

[81] Hultman CM, Ohman A, Cnattingius S, Wieselgren IM, Lindström LH (1997). Prenatal and neonatal risk factors for schizophrenia. Br J Psychiatr.

[82] Preti A, Cardascia L, Zen T, Marchetti M, Favaretto G, Miotto P (2000). Risk for obstetric complications and schizophrenia. Psychiatry Res.

[83] Rosso IM, Cannon TD, Huttunen T, Huttunen MO, Lonnqvist J, Gasperoni TL (2000). Obstetric risk factors for early-onset schizophrenia in a Finnish birth cohort. Am J Psychiatr.

[84] Parnas J, Schulsinger F, Teasdale TW, Schulsinger H, Feldman PM, Mednick SA (1982). Perinatal complications and clinical outcome within the schizophrenia spectrum. Br J Psychiatr.

[85] Haukka JK, Suvisaari J, Lonnqvist J (2004). Family structure and risk factors for schizophrenia: case-sibling study. BMC Psychiatry.

[86] Brown AS, Schaefer CA, Wyatt RJ, Begg MD, Goetz R, Bresnahan MA, Harkavy-Friedman J, Gorman JM, Malaspina D, Susser ES (2006). Paternal age and risk of schizophrenia in adult offspring. Am J Psychiatr.

[87] Perrin M, Harlap S, Kleinhaus K, Lichtenberg P, Manor O, Draiman B, Fennig S, Malaspina D (2010). Older paternal age strongly increases the morbidity for schizophrenia in sisters of affected females. Am J Med Genet B Neuropsychiatr Genet.

[88] Sipos A, Rasmussen F, Harrison G, Tynelius P, Lewis G, Leon DA, Gunnell D (2004). Paternal Age And Schizophrenia: A Population Based Cohort Study. Br Med J.

[89] Buizer-Voskamp JE, Laan W, Staal WG, Hennekam EAM, Aukes MF, Termorshuizen F, Kahn RS, Boks MPM, Ophoff RA (2011). Paternal age and psychiatric disorders: Findings from a Dutch population registry. Schizophr Res.

[90] Menezes PR, Lewis G, Rasmussen F, Zammit S, Sipos A, Harrison GL, Tynelius P, Gunnell D (2010). Paternal and maternal ages at conception and risk of bipolar affective disorder in their offspring. Psychol Med.

[91] Werner S, Malaspina D, Rabinowitz J (2007). Socioeconomic status at birth is associated with risk of schizophrenia: population-based multilevel study. Schizophr Bull.

[92] Corcoran C, Perrin M, Harlap S, Deutsch L, Fennig S, Manor O, Nahon D, Kimhy D, Malaspina D, Susser E (2009). Effect of socioeconomic status and parents’ education at birth on risk of schizophrenia in offspring. Soc Psychiatry Psychiatr Epidemiol.

[93] Koponen H, Rantakallio P, Veijola J, Jones P, Jokelainen J, Isohanni M (2004). Childhood central nervous system infections and risk for schizophrenia. Eur Arch Psychiatry Clin Neurosci.

[94] Makikyro T, Isohanni M, Moring J, Oja H, Hakko H, Jones P, Rantakallio P (1997). Is a child's risk of early onset schizophrenia increased in the highest social class?. Schizophr Res.

[95] Wicks S, Hjern A, Gunnell D, Lewis G, Dalman C (2005). Social adversity in childhood and the risk of developing psychosis: a national cohort study. Am J Psychiatry.

[96] Seidman LJ, Cherkerzian S, Goldstein JM, Agnew-Blais J, Tsuang MT, Buka SL (2013). Neuropsychological performance and family history in children at age 7 who develop adult schizophrenia or bipolar psychosis in the New England Family Studies. Psychol Med.

[97] Mulvany F, O'Callaghan E, Takei N, Byrne M, Fearon P, Larkin C (2001). Effect of social class at birth on risk and presentation of schizophrenia: case-control study. BMJ.

[98] Castle DJ, Scott K, Wessely S, Murray RM (1993). Does social deprivation during gestation and early life predispose to later schizophrenia?. Soc Psychiatry Psychiatr Epidemiol.

[99] Hare EH, Price JS, Slater E (1972). Parental social class in psychiatric patients. Br J Psychiatry.

[100] Harrison G, Gunnell D, Glazebrook C, Page K, Kwiecinski R (2001). Association between schizophrenia and social inequality at birth: case-control study. Br J Psychiatry.

[101] Marcelis M, Navarro-Mateu F, Murray R, Selten J-P, Van Os J (1998). Urbanization and psychosis: a study of 1942–1978 birth cohorts in The Netherlands. Psychol Med.

[102] Selten JP, Cantor-Graae E, Kahn RS (2007). Migration and schizophrenia. Curr Opin Psychiatry.

[103] Corcoran C, Perrin M, Harlap S, Deutsch L, Fennig S, Manor O, Nahon D, Kimhy D, Malaspina D, Susser E (2009). Incidence of schizophrenia among second-generation immigrants in the jerusalem perinatal cohort. Schizophr Bull.

[104] Bresnahan M, Begg MD, Brown A, Schaefer C, Sohler N, Insel B, Vella L, Susser E (2007). Race and risk of schizophrenia in a US birth cohort: another example of health disparity?. Int J Epidemiol.

[105] Burman B, Mednick SA, Machon RA, Parnas J, Schulsinger F (1987). Children at high risk for schizophrenia: parent and offspring perceptions of family relationships. J Abnorm Psychol.

[106] Goldstein MJ (1987). The UCLA High-Risk Project. Schizophr Bull.

[107] Schiffman J, LaBrie J, Carter J, Cannon T, Schulsinger F, Parnas J, Mednick S (2002). Perception of parent–child relationships in high-risk families, and adult schizophrenia outcome of offspring. J Psychiatr Res.

[108] Walker E, Hoppes E, Emory E, Mednick S, Schulsinger F (1981). Environmental factors related to schizophrenia in psychophysiologically labile high-risk males. J Abnorm Psychol.

[109] Riordan DV, Morris C, Hattie J, Stark C (2012). Interbirth spacing and offspring mental health outcomes. Psychol Med.

[110] Dalman C, Allebeck P, Gunnell D, Harrison G, Kristensson K, Lewis G, Lofving S, Rasmussen F, Wicks S, Karlsson H (2008). Infections in the CNS during childhood and the risk of subsequent psychotic illness: a cohort study of more than one million Swedish subjects. Am J Psychiatr.

[111] Leask SJ, Done DJ, Crow TJ (2002). Adult psychosis, common childhood infections and neurological soft signs in a national birth cohort. Br J Psychiatr.

[112] Rantakallio P, Jones P, Moring J, Von Wendt L (1997). Association between central nervous system infections during childhood and adult onset schizophrenia and other psychoses: a 28-year follow-up. Int J Epidemiol.

[113] Orlovska S, Pedersen MS, Benros ME, Mortensen PB, Agerbo E, Nordentoft M (2014). Head injury as risk factor for psychiatric disorders: a nationwide register-based follow-up study of 113,906 persons with head injury. Am J Psychiatr.

[114] Niemi LT, Suvisaari JM, Haukka JK, Lonnqvist JK (2005). Childhood growth and future development of psychotic disorder among Helsinki high-risk children. Schizophr Res.

[115] Arseneault L, Cannon M, Poulton R, Murray R, Caspi A, Moffitt TE (2002). Cannabis use in adolescence and risk for adult psychosis: longitudinal prospective study. Br Med J.

[116] Welham J, Scott J, Williams G, Najman J, Bor W, O'Callaghan M, McGrath J (2009). Emotional and behavioural antecedents of young adults who screen positive for non-affective psychosis: a 21-year birth cohort study. Psychol Med.

[117] Bearden CE, Rosso IM, Hollister JM, Sanchez LE, Hadley T, Cannon TD (2000). A prospective cohort study of childhood behavioral deviance and language abnormalities as predictors of adult schizophrenia. Schizophr Bull.

[118] Meyer SE, Carlson GA, Youngstrom E, Ronsaville DS, Martinez PE, Gold PW, Hakak R, Radke-Yarrow M (2009). Long-term outcomes of youth who manifested the CBCL-Pediatric Bipolar Disorder phenotype during childhood and/or adolescence. J Affect Disord.

[119] Achenbach TM, Rescorla LA (2001). Manual for the ASEBA School-Age Forms and Profiles.

[120] Crow TJ, Done DJ, Sacker A (1995). Chidhood precursors of psychiosis as clues to its evolutionary orgins. Eur Arch Psychiatry Clin Neurosci.

[121] Done DJ, Crow TJ, Johnstone EC, Sacker A (1994). Childhood antecedents of schizophrenia and affective illness: Social adjustment at ages 7 and 11. Br Med J.

[122] Ekstrom M, Sorensen H, Mednick SA (2006). Premorbid personality in schizophrenia spectrum: A prospective study. Nord J Psychiatry.

[123] Fisher HL, Caspi A, Poulton R, Meier MH, Houts R, Harrington H, Arseneault L, Moffitt TE (2013). Specificity of childhood psychotic symptoms for predicting schizophrenia by 38 years of age: a birth cohort study. Psychol Med.

[124] Poulton R, Caspi A, Moffitt TE, Cannon M, Murray R, Harrington H (2000). Children's self-reported psychotic symptoms and adult schizophreniform disorder: A 15-year longitudinal study. Arch Gen Psychiatry.

[125] Ott SL, Roberts S, Rock D, Allen J, Erlenmeyer-Kimling L (2002). Positive and negative thought disorder and psychopathology in childhood among subjects with adulthood schizophrenia. Schizophr Res.

[126] Schiffman J, Walker E, Ekstrom M, Schulsinger F, Sorensen H, Mednick S (2004). Childhood videotaped social and neuromotor precursors of schizophrenia: a prospective investigation. Am J Psychiatr.

[127] Carlson GA, Weintraub S (1993). Childhood behavior problems and bipolar disorder - relationship or coincidence?. J Affect Disord.

[128] Kim-Cohen J, Caspi A, Moffitt TE, Harrington H, Milne BJ, Poulton R (2003). Prior juvenile diagnoses in adults with mental disorder: Developmental follow-back of a prospective-longitudinal cohort. Arch Gen Psychiatry.

[129] Ullman VZ, Levine SZ, Reichenberg A, Rabinowitz J (2012). Real-world premorbid functioning in schizophrenia and affective disorders during the early teenage years: A population-based study of school grades and teacher ratings. Schizophr Res.

[130] Cornblatt BA, Obuchowski M, Roberts S, Pollack S, Erlenmeyer–Kimling L (1999). Cognitive and behavioral precursors of schizophrenia. Dev Psychopathol.

[131] Stringaris A, Cohen P, Pine DS, Leibenluft E (2009). Adult Outcomes of Youth Irritability: A 20-Year Prospective Community-Based Study. Am J Psychiatr.

[132] Reichart CG, van der Ende J, Wals M, Hillegers MHJ, Nolen WA, Ormel J, Verhulst FC (2005). The use of the GBI as predictor of bipolar disorder in a population of adolescent offspring of parents with a bipolar disorder. J Affect Disord.

[133] Cannon TD, Bearden CE, Hollister JM, Rosso IM, Sanchez LE, Hadley T (2000). Childhood Cognitive Functioning in Schizophrenia Patients and Their Unaffected Siblings: A Prospective Cohort Study. Schizophr Bull.

[134] Jones P, Murray R, Rodgers B, Marmot M (1994). Child developmental risk factors for adult schizophrenia in the British 1946 birth cohort. Lancet.

[135] Koenen KC, Moffitt TE, Roberts AL, Martin LT, Kubzansky L, Harrington H, Poulton R, Caspi A (2009). Childhood IQ and adult mental disorders: a test of the cognitive reserve hypothesis. Am J Psychiatr.

[136] Niendam TA, Bearden CE, Rosso IM, Sanchez LE, Hadley T, Nuechterlein KH, Cannon TD (2003). A prospective study of childhood neurocognitive functioning in schizophrenic patients and their siblings. Am J Psychiatr.

[137] Schulz J, Sundin J, Leask S, Done DJ (2014). Risk of adult schizophrenia and its relationship to childhood IQ in the 1958 British birth cohort. Schizophr Bull.

[138] Chong S, Subramaniam M, Lee IM, Pek E, Cheok C, Verma S, Wong J (2009). Academic attainment: a predictor of psychiatric disorders?. Soc Psychiatry Psychiatr Epidemiol.

[139] Osler M, Lawlor DA, Nordentoft M (2007). Cognitive function in childhood and early adulthood and hospital admission for schizophrenia and bipolar disorders in Danish men born in 1953. Schizophr Res.

[140] Meyer SE, Carlson GA, Wiggs EA, Martinez PE, Ronsaville DS, Klimes-dougan B, Gold PW, Radke-yarrow M (2004). A prospective study of the association among impaired executive functioning, childhood attentional problems, and the development of bipolar disorder. Dev Psychopathol.

[141] Ott SL, Spinelli S, Rock D, Roberts S, Amminger GP, Erlenmeyer-Kimling L (1998). The New York High-Risk Project: social and general intelligence in children at risk for schizophrenia. Schizophr Res.

[142] Sørensen HJ, Mortensen EL, Schiffman J, Ekstrøm M, Denenney D, Mednick SA (2010). Premorbid IQ and adult schizophrenia spectrum disorder: Verbal Performance subtests. Psychiatry Res.

[143] Kremen WS, Vinogradov S, Poole JH, Schaefer CA, Deicken RF, Factor-Litvak P, Brown AS (2010). Cognitive decline in schizophrenia from childhood to midlife: a 33-year longitudinal birth cohort study. Schizophr Res.

[144] Woodberry KA, Giuliano AJ, Seidman LJ (2008). Premorbid IQ in schizophrenia: a meta-analytic review. Am J Psychiatry.

[145] Isohanni I, Jarvelin M-R, Nieminen P, Jones P, Rantakallio P, Jokelainen J, Isohanni M (1998). School performance as a predictor of psychiatric hospitalization in adult life. A 28-year follow-up in the Northern Finland 1966 Birth Cohort. Psychol Med.

[146] MacCabe JH, Wicks S, Lofving S, David AS, Berndtsson A, Gustafsson JE, Allebeck P, Dalman C (2013). Decline in cognitive performance between ages 13 and 18 years and the risk for psychosis in adulthood: a Swedish longitudinal cohort study in males. JAMA Psychiatry.

[147] Sorensen HJ, Mortensen EL, Parnas J, Mednick SA (2006). Premorbid neurocognitive functioning in schizophrenia spectrum disorder. Schizophr Bull.

[148] Mouridsen SE, Hauschild K-M (2008). A longitudinal study of schizophrenia- and affective spectrum disorders in individuals diagnosed with a developmental language disorder as children. J Neural Transm.

[149] Griffith JJ, Mednick SA, Schulsinger F, Diderichsen B (1980). Verbal associative disturbances in children at high risk for schizophrenia. J Abnorm Psychol.

[150] Isohanni M, Jones PB, Moilanen K, Rantakallio P, Veijola J, Oja H, Koiranen M, Jokelainen J, Croudace T, Järvelin MR (2001). Early developmental milestones in adult schizophrenia and other psychoses. A 31-year follow-up of the Northern Finland 1966 Birth Cohort. Schizophrenia Res.

[151] Rosso IM, Bearden CE, Hollister JM, Gasperoni TL, Sanchez LE, Hadley T, Cannon TD (2000). Childhood neuromotor dysfunction in schizophrenia patients and their unaffected siblings: a prospective cohort study. Schizophr Bull.

[152] Cannon M, Jones P, Murray RM, Wadsworth ME (1997). Childhood laterality and later risk of schizophrenia in the 1946 British birth cohort. Schizophr Res.

[153] Erlenmeyer-Kimling L, Rock D, Roberts SA, Janal M, Kestenbaum C, Cornblatt B, Adamo UH, Gottesman II (2000). Attention, memory, and motor skills as childhood predictors of schizophrenia-related psychoses: the New York High-Risk Project. Am J Psychiatr.

[154] Schiffman J, Ekstrom M, LaBrie J, Schulsinger F, Sorensen H, Mednick S (2002). Minor physical anomalies and schizophrenia spectrum disorders: a prospective investigation. Am J Psychiatr.

[155] Schiffman J, Maeda JA, Hayashi K, Michelsen N, Sorensen HJ, Ekstrom M, Abe KA, Chronicle EP, Mednick SA (2006). Premorbid childhood ocular alignment abnormalities and adult schizophrenia-spectrum disorder. Schizophr Res.

[156] Schiffman J, Pestle S, Mednick S, Ekstrom M, Sorensen H, Mednick S (2005). Childhood laterality and adult schizophrenia spectrum disorders: a prospective investigation. Schizophr Res.

[157] Schiffman J, Sorensen HJ, Maeda J, Mortensen EL, Victoroff J, Hayashi K, Michelsen NM, Ekstrom M, Mednick S (2009). Childhood motor coordination and adult schizophrenia spectrum disorders. Am J Psychiatr.

[158] Matheson SL, Shepherd AM, Carr VJ (2014). How much do we know about schizophrenia and how well do we know it? Evidence from the Schizophrenia Library. Psychol Med.

[159] Matheson SL, Shepherd AM, Draganic D, Carr VJ (2011). A new web-based Schizophrenia Library--evidence compiled and graded systematically. Schizophr Res.

[160] Murray RM, Lewis SW (1987). Is schizophrenia a neurodevelopmental disorder?. Br Med J (Clin Res Ed).

[161] Weinberger DR (1987). Implications of normal brain development for the pathogenesis of schizophrenia. Arch Gen Psychiatry.

[162] Howes OD, Murray RM (2014). Schizophrenia: an integrated sociodevelopmental-cognitive model. Lancet.

[163] Laurens KR, Hodgins S, Maughan B, Murray RM, Rutter ML, Taylor EA (2007). Community screening for psychotic-like experiences and other putative antecedents of schizophrenia in children aged 9–12 years. Schizophr Res.

[164] Werbeloff N, Drukker M, Dohrenwend BP, Levav I, Yoffe R, van Os J, Davidson M, Weiser M (2012). Self-reported attenuated psychotic symptoms as forerunners of severe mental disorders later in life. Arch Gen Psychiatry.

