# Supplementary material for: Common or distinct pathways to psychosis? A systematic review of evidence from prospective studies for developmental risk factors and antecedents of the schizophrenia spectrum disorders and affective psychoses
Source: BMC Psychiatry. 2015 Aug 25;15:205. doi: 10.1186/s12888-015-0562-2 (PMC4548447; doi:10.1186/s12888-015-0562-2)
Supplement: Additional file 1: — PRISMA. (DOCX 24 kb) [file 12888_2015_562_MOESM1_ESM.docx]

**Index to Supplementary Tables 1-4: Page**

**Supplementary Table 1: Conception, pregnancy, and birth risk factors 2**

Maternal psychological factors 2

Maternal illness during pregnancy 2

Previous pregnancies 5

Other physical factors 5

Obstetric complications 7

**Supplementary Table 2: Demographic or familial risk factors 13**

Maternal age at birth 13

Paternal age at birth 13

Parental education 15

Socio-economic status 16

Urbanicity 17

Migration and ethnicity 17

Family factors (relationships, affective style, etc.) 18

Sibship 19

**Supplementary Table 3: Childhood and adolescent risk factors 21**

Childhood illness 21

Other physical factors 21

Substance misuse 22

**Supplementary Table 4: Childhood and adolescent antecedents 23**

Social, emotional, and behavioural functioning and psychosis symptoms 23

Cognitive functioning 26

Language functioning 29

Motor functioning, and developmental motor milestones 29

**Supplementary Table 1: Conception, pregnancy, and birth risk factors**

| **Study citation** | **Sample size (n)** | | **Outcome (Diagnosis)** | **Age at assessment**  **(years)** | | **Exposure (Risk factor)** | **Outcome: % with risk factor** | | | | | **Effect size (CI)** | |
| --- | --- | --- | --- | --- | --- | --- | --- | --- | --- | --- | --- | --- | --- |
|  | **Cases** | **Controls** |  | **Outcome** | **Exposure** | **Measure** | **SSD** | **not-SSD** | | **AP** | **not-AP** | **SSD (vs. not-SSD)** | **AP (vs. not-AP)** |
| **Maternal psychological factors** | | | | | | | | | | | | | |
| *[*[*1*](#_ENREF_1)*] Herman et al (2006)* | 70 | 7725 | SSD | 31-38 | Pregnancy | Unwantedness of pregnancy (ambivalent/negative attitude) | 22.9 | | 19.4 |  |  | HR 1.79 (0.91-3.50)‡ |  |
| *[*[*2*](#_ENREF_2)*] Khashan et al (2008)* | 7331 | 1 372 669 | SZ | 12-35 | Pregnancy | Death or severe illness of a close relative:  - Before pregnancy |  | |  |  |  | RR 0.96 (0.74-1.26)‡ |  |
|  |  |  |  |  |  | - 1st trimester |  |  |  |  |  | RR 1.44 (0.96-2.16)‡ |  |
|  |  |  |  |  |  | - 2nd trimester |  |  |  |  |  | RR 1.35 (0.87-2.11)‡ |  |
|  |  |  |  |  |  | - 3rd trimester |  |  |  |  |  | RR 1.12 (0.74-1.71)‡ |  |
| *[*[*3*](#_ENREF_3)*] Maki et al (2010)* | 151 | 10 507 | SZ and other psychoses | Mean 35 | (24^th^-28^th^ gestational week) | Maternal depressed mood during pregnancy | 17.2 | | 13.9 |  |  | OR 1.30 (0.90-2.00)‡ |  |
|  |  |  |  |  |  | Mother with psychosis | 2.6 | | 0.5 |  |  | OR 4.60 (2.60-7.90)*‡ |  |
|  |  |  |  |  |  | Father with psychosis | 4.0 | | 0.3 |  |  | OR 3.60 (1.80-7.20)*‡ |  |
| *[*[*4*](#_ENREF_4)*] Niemi et al (2004)* | 10 | 164 | SSD | 35-39 |  | Maternal psychotic symptoms (composite score) |  | |  |  |  | OR 0.58 (0.18-1.84) |  |
| **Maternal illness during pregnancy** | | | | | | | | | | | | | |
| *[*[*5*](#_ENREF_5)*] Babulus et al (2006)* | 71 | 7723 | SSD | 30-38 | Pregnancy | Maternal genital and reproductive infections:  - Periconception |  | |  |  |  | RR 5.03 (2.00 -12.64)**‡ |  |
|  |  |  |  |  |  | - 1st trimester |  | |  |  |  | RR 0.91 (0.22 -3.75)‡ |  |
|  |  |  |  |  |  | - 2nd trimester |  | |  |  |  | RR 0.33 (0.05 -2.37)‡ |  |
|  |  |  |  |  |  | - 3rd trimester |  | |  |  |  | RR 1.37 (0.33 – 5.63)‡ |  |
| *[*[*6*](#_ENREF_6)*] Bain et al (2000);*  *[*[*7*](#_ENREF_7)*] Kendell et al (2000)* | 296; 217 | 296;  217 | SZ (Kendall); AP (Bain) | 22-26 | Pre-pregnancy, pregnancy | Pre-existing maternal physical illness | 3.0 | | 3.0 | 1.4 | 2.3 | OR 1.00 (0.39-2.57) | OR 0.60 (0.14-2.64) |
| *[*[*8*](#_ENREF_8)*] Brown et al (2000)* | 58 | 7725 | SSD | 31-38 | Pregnancy | Maternal respiratory infection:  - 1st trimester |  | |  |  |  | RR 0.86 (0.28-2.84)‡ |  |
|  |  |  |  |  |  | - 2nd trimester | 15.5 | | 0.7 |  |  | RR 2.13 (1.05-4.35)*‡ |  |
|  |  |  |  |  |  | - 3rd trimester |  | |  |  |  | RR 0.68 (0.25-1.87)‡ |  |

| **Study citation** | **Sample size (n)** | | **Outcome (Diagnosis)** | **Age at assessment**  **(years)** | | **Exposure (Risk factor)** | **Outcome: % with risk factor** | | | | | **Effect size (CI)** | |
| --- | --- | --- | --- | --- | --- | --- | --- | --- | --- | --- | --- | --- | --- |
|  | **Cases** | **Controls** |  | **Outcome** | **Exposure** | **Measure** | **SSD** | **not-SSD** | | **AP** | **not-AP** | **SSD (vs. not-SSD)** | **AP (vs. not-AP)** |
| *[*[*9*](#_ENREF_9)*] Brown et al (2004)* | 64 | 125 | SSD | 30-38 | Pregnancy | Prenatal influenza:  - 1st trimester | 25.0 | | 11.0 |  |  | OR 2.70 (1.22-5.98)* |  |
|  |  |  |  |  |  | - 2nd trimester | 15.0 | | 13.0 |  |  | OR 1.18 (0.50-2.80) |  |
|  |  |  |  |  |  | - 3rd trimester | 19.0 | | 17.0 |  |  | OR 1.15 (0.53-2.50) |  |
|  |  |  |  |  |  | - 1st half of pregnancy | 21.9 | | 9.9 |  |  | OR 2.69 (1.14-6.34)* |  |
|  |  |  |  |  |  | - 2nd half of pregnancy | 20.9 | | 24.9 |  |  | OR 0.79 (0.38-1.65) |  |
| *[*[*10*](#_ENREF_10)*] Brown et al (2004)* | 59 | 105 | SSD | 30-38 | Pregnancy | Elevated maternal interleukin-8 levels |  | |  |  |  | OR 1.96 (1.10-3.52)* |  |
| *[*[*11*](#_ENREF_11)*] Brown et al (2005)* | 63 | 123 | SSD | 31-38 | Pregnancy | Maternal antibody toxoplasmosis (high) | 20.6 | | 10.6 |  |  | OR 2.19 (0.95-5.06) |  |
| *[*[*12*](#_ENREF_12)*] Brown et al (2006)* | 60 | 110 | SSD | 30-38 | Pregnancy | Herpes Virus:  - Simplex virus type 2 | 26.7 | | 22.2 |  |  | OR 1.13 (0.51–2.55)‡ |  |
|  |  |  |  |  |  | - Simplex virus type 1 | 68.3 | | 64.2 |  |  | OR 0.98 (0.47–2.08)‡ |  |
|  |  |  |  |  |  | Cytomegalovirus | 71.7 | | 67.6 |  |  | OR 1.08 (0.49–2.35)‡ |  |
| *[*[*13*](#_ENREF_13)*] Buka et al (2008)* | 108; 85 | 544 | SSD; AP | 18-37 | Pregnancy | Herpes simplex virus type 2 | 36.1 | | 24.8 | 24.7 | 20.4 | OR 1.80 (1.10- 3.00)*‡ | OR 1.30 (0.70-2.40)‡ |
| *[*[*14*](#_ENREF_14)*] Canetta et al (in press)* | 777 | 777 | SZ; SZAFF |  | Pregnancy | Higher maternal C-reactive protein levels |  | |  |  |  | OR 1.28 (1.07-1.54)**‡ |  |
| *[*[*15*](#_ENREF_15)*] Canetta et al (2014)* | 36 | 72 | BD |  | Pregnancy | Maternal influenza (serum):  - Any trimester |  | |  | 38.9 | 18.1 |  | OR 5.03 (1.38-18.38)*‡ |
|  |  |  |  |  |  | - First trimester |  | |  | 33.3 | 14.3 |  | OR 3.36 (0.83-13.55)‡ |
|  |  |  |  |  |  | - Second trimester |  | |  | 20.0 | 8.3 |  | OR 4.00 (0.77-20.87)‡ |
|  |  |  |  |  |  | - Third trimester |  | |  | 4.2 | 4.2 |  | OR 1.00 (0.05-18.92)‡ |
| *[*[*16*](#_ENREF_16)*] Clarke et al (2009)* | 71 | 23 333 | SZ |  |  | Infection (with family history of psychosis) | 58.3 | | 42.6 |  |  | OR 1.88 (0.60-5.96) |  |
|  |  |  |  |  |  | Infection (no family history of psychosis) | 49.1 | | 41.5 |  |  | OR 1.36 (0.82-2.27) |  |
| *[*[*17*](#_ENREF_17)*] Hultman et al (1999)* | 167; 198 | 835; 990 | SZ; AP | 15-21 | Pregnancy | Hypertensive diseases | 5.4 | | 3.2 | 4.5 | 3.0 | OR 1.3 (0.6-3.1)‡ | OR 1.6 (0.7-3.5)‡ |
|  |  |  |  |  |  | Diabetes | 1.2 | | 0.2 | 0.5 | 0.6 | OR 7.8 (0.9-67.6)‡ | OR 0.7 (0.1-6.8)‡ |

| **Study citation** | **Sample size (n)** | | **Outcome (Diagnosis)** | **Age at assessment**  **(years)** | | **Exposure (Risk factor)** | **Outcome: % with risk factor** | | | | | **Effect size (CI)** | |
| --- | --- | --- | --- | --- | --- | --- | --- | --- | --- | --- | --- | --- | --- |
|  | **Cases** | **Controls** |  | **Outcome** | **Exposure** | **Measure** | **SSD** | **not-SSD** | | **AP** | **not-AP** | **SSD (vs. not-SSD)** | **AP (vs. not-AP)** |
| *[*[*18*](#_ENREF_18)*] Jones et al (1998)* | 70 | 1074^ | SZ | 27-28 | Pregnancy | Maternal diabetes | 1.4 | | 0.1 |  |  | OR 7.70 (0.40 - 135.00)‡ |  |
|  |  |  |  |  |  | Maternal heart disease | 0 | | 0.4 |  |  | OR 1.58 (0.09-29.42) |  |
|  |  |  |  |  |  | Maternal chronic pulmonary disorder | 2.9 | | 1.2 |  |  | OR 2.60 (0.60–12.20)‡ |  |
|  |  |  |  |  |  | Maternal anaemia in 2nd trimester | 12.9 | | 7.7 |  |  | OR 1.70 (0.80-3.70)‡ |  |
|  |  |  |  |  |  | Maternal hypertension | 0.0 | | 0.3 |  |  | OR 2.04 (0.11-39.47) |  |
|  |  |  |  |  |  | Preeclampsia | 1.4 | | 3.0 |  |  | OR 0.50 (0.10-3.40)‡ |  |
| *[*[*19*](#_ENREF_19)*] Kawai et al (2004)* | 52 | 284 | SZ | Mean ~20 | Pregnancy | Physical illness in mother | 3.8 | | 4.6 |  |  | OR 0.82 (0.18-3.77) |  |
| *[*[*20*](#_ENREF_20)*] Mortensen et al (2010)* | 602 | 602 | SSD | ≤24 | Pregnancy | Herpes simplex virus type 2 | 16.1 | | 11.1 |  |  | OR 1.54 (1.10-2.15)*  IRR 1.51 (1.08–2.10)*‡ |  |
| *[*[*21*](#_ENREF_21)*] Nielsen et al (2013)* | 3722 | 1 112 030 | SZ | 21-31 | Pregnancy | Maternal treated infection |  | |  |  |  | IRR 1.23 (1.04-1.44)*‡ |  |
|  |  |  |  |  |  | Paternal treated infection |  | |  |  |  | IRR 1.31 (0.87-1.88)‡ |  |
| *[*[*22*](#_ENREF_22)*] Parboosing et al (2013)* | 92 | 722 | BD |  |  | Maternal influenza:  - Any trimester |  | |  | 8.7 | 2.6 |  | OR 4.21 (1.60-11.05)**‡ |
|  |  |  |  |  |  | - Periconception |  | |  | 1.1 | 0.6 |  | OR 4.20 (0.38-47.06)‡ |
|  |  |  |  |  |  | - 1st trimester |  | |  | 2.2 | 1.2 |  | OR 2.21 (0.41-11.79)‡ |
|  |  |  |  |  |  | - 2nd trimester |  | |  | 2.2 | 0.4 |  | OR 7.74 (0.65-92.70)‡ |
|  |  |  |  |  |  | - 3rd trimester |  | |  | 3.3 | 0.7 |  | OR 5.68 (1.07-30.10)*‡ |
| *[*[*23*](#_ENREF_23)*] Sorensen et al (2009)* | 153 | 7788 | SZ | 45-47 | Pregnancy | Prenatal bacterial infection:  - Upper respiratory (except pneumonia) | 7.8 | | 2.6 |  |  | OR 3.17 (1.73-5.82)** |  |
|  |  |  |  |  |  | - Pneumonia | 0 | | 0.2 |  |  | OR 1.84 (0.11-31.12) |  |
|  |  |  |  |  |  | - Urinary tract infections (mainly cystitis) | 8.5 | | 8.3 |  |  | OR 1.03 (0.58-1.82) |  |
|  |  |  |  |  |  | - Gonococcal infection | 3.3 | | 1.1 |  |  | OR 3.07 (1.23-7.64)* |  |
|  |  |  |  |  |  | - Any other bacterial infection | 0.9 | | 1.2 |  |  | OR 0.75 (0.14-4.05) |  |
|  |  |  |  |  |  | Any prenatal bacterial infection:  - 1st trimester |  | |  |  |  | OR 2.14 (1.06-4.31)*‡ |  |
|  |  |  |  |  |  | - 2nd trimester |  | |  |  |  | OR 1.60 (0.91-2.80)‡ |  |
|  |  |  |  |  |  | - 3rd trimester |  | |  |  |  | OR 1.14 (0.59-2.22)‡ |  |

| **Study citation** | **Sample size (n)** | | **Outcome (Diagnosis)** | **Age at assessment**  **(years)** | | **Exposure (Risk factor)** | **Outcome: % with risk factor** | | | | | **Effect size (CI)** | |
| --- | --- | --- | --- | --- | --- | --- | --- | --- | --- | --- | --- | --- | --- |
|  | **Cases** | **Controls** |  | **Outcome** | **Exposure** | **Measure** | **SSD** | **not-SSD** | | **AP** | **not-AP** | **SSD (vs. not-SSD)** | **AP (vs. not-AP)** |
| *[*[*24*](#_ENREF_24)*] Suvisaari et al (2012)* | 29 | 208^ | SSD | 37-41 | Pregnancy | Maternal treated illness | 6.9 | | 7.9 |  |  | OR 0.86 (0.19-3.96) |  |
| *[*[*25*](#_ENREF_25)*] Talovic et al (1980)* | 15 | 102 | SZ | Mean 25.1 | Pregnancy | Physical illness during pregnancy | 21.0 | | 4.0 |  |  | OR 6.38 (1.30-31.25)* |  |
| ***Previous pregnancies*** | | | | | | | | | | | | | |
| *[*[*14*](#_ENREF_14)*] Canetta et al (in press)* | 777 | 777 | SZ; SZAFF |  |  | Previous pregnancies ≥1 (vs. 0) |  | |  |  |  | OR 1.06 (0.86-1.29) |  |
| *[*[*17*](#_ENREF_17)*] Hultman et al (1999)* | 167; 198 | 835; 990 | SZ; AP | 15-21 |  | Parity 1 (vs. 2-3) | 47.9 | | 44.5 | 48.0 | 48.8 | OR 1.3 (0.6-1.9)‡ | OR 1.0 (0.7-1.4)‡ |
|  |  |  |  |  |  | Parity 4 (vs. 2-3) | 9.6 | | 5.3 | 5.6 | 5.0 | OR 2.0 (1.0-3.8)*‡ | OR 1.0 (0.5-2.2)‡ |
| *[*[*26*](#_ENREF_26)*] Kemppainen et al (2000)* | 89 | 10630^ | SZ | Mean 28 | Birth | Grand multiparity (6+) | 15.7 | | 11.9 |  |  | OR 1.38 (0.78-2.45) |  |
| *[*[*27*](#_ENREF_27)*] Nosarti et al (2012)* | 669; 217 | 1 300 853;  1 301 305 | Non-AP (SZ); BD | Mean 23 (17-29) | Birth | Parity 1 (vs. all other) | 13.3 | | 7.2 | 6.9 | 7.2 | OR 1.98 (1.58-2.47)** | OR 0.96 (0.57-1.61) |
|  |  |  |  |  |  | Parity 2-3 (vs. all other) | 64.3 | | 74.7 | 40.0 | 74.7 | OR 0.61 (0.52-0.71)** | OR 0.23 (0.17-0.30)** |
|  |  |  |  |  |  | Parity ≥4 (vs. all other) | 22.4 | | 18.1 | 11.1 | 18.1 | OR 1.31 (1.09-1.57)** | OR 0.56 (0.37-0.86)* |
| *[*[*28*](#_ENREF_28)*] Sacker et al (1995)* | 35; 32 | 16812 | SZ; AP | Mean 28 | Birth | Mother >2 previous babies | 32.4 | | 16.6 | 25.0 | 16.6 | OR 2.41 (1.18-4.89)* | OR 1.67 (0.75-3.73) |
|  |  |  |  |  |  | Mother had previous babies >4000g | 8.8 | | 9.3 | 18.8 | 9.3 | OR 0.94 (0.29-3.03) | OR 2.26 (0.93-5.49) |
| *[*[*24*](#_ENREF_24)*] Suvisaari et al (2012)* | 29 | 208^ | SSD | 37-41 | Birth | Maternal miscarriages | 17.2 | | 27.9 |  |  | OR 0.54 (0.20-1.48) |  |
|  |  |  |  |  |  | Maternal abortions | 3.5 | | 3.4 |  |  | OR 1.03 (0.12-8.56) |  |
| ***Other physical factors*** | | | | | | | | | | | | | |
| *[*[*29*](#_ENREF_29)*] Bao et al (2012)* | 55 | 106 | SSD | 31-38 | Pregnancy | Low maternal retinol (lowest tertile) during:  - 2nd trimester | 45.5 | | 32.9 |  |  | OR 3.06 (1.06-8.79)*‡ |  |
|  |  |  |  |  |  | - 3rd trimester | 38.3 | | 33.3 |  |  | OR 1.59 (0.47-5.38)‡ |  |
| *[*[*10*](#_ENREF_10)*] Brown et al (2004)* | 59 | 105 | SSD | 30-38 |  | Maternal smoking | 43.0 | | 45.0 |  |  | OR 0.92 (0.48-1.75) |  |
| *[*[*30*](#_ENREF_30)*] Carter et al (2003)* | 33 | 70^ | SZ | 39-42 | 0-14 | Winter birth |  | |  |  |  | OR 1.16 (0.54-2.48)# |  |
| *[*[*31*](#_ENREF_31)*] Harper et al (2011)* | 57 | 95 | SSD | 31-38 | Pregnancy | Maternal serum docosahexaenoic acid (highest tertile) | 56.1 | | 33.7 |  |  | OR 2.51 (1.28-4.93)* |  |
| *[*[*17*](#_ENREF_17)*] Hultman et al (1999)* | 167; 198 | 835; 990 | SZ; AP | 15-21 |  | Late winter birth (January-April) | 43.7 | | 36.3 | 43.9 | 36.2 | OR 1.4 (1.0-2.0)*‡ | OR 1.5 (1.1-2.0)*‡ |

| **Study citation** | | **Sample size (n)** | | | **Outcome (Diagnosis)** | **Age at assessment**  **(years)** | | **Exposure (Risk factor)** | **Outcome: % with risk factor** | | | | | **Effect size (CI)** | |
| --- | --- | --- | --- | --- | --- | --- | --- | --- | --- | --- | --- | --- | --- | --- | --- |
|  | | **Cases** | **Controls** | |  | **Outcome** | **Exposure** | **Measure** | **SSD** | **not-SSD** | | **AP** | **not-AP** | **SSD (vs. not-SSD)** | **AP (vs. not-AP)** |
| *[*[*18*](#_ENREF_18)*] Jones et al (1998)* | | 70 | 1074^ | | SZ | 27-28 | Pregnancy | Smoking in 2nd trimester | 16.2 | | 14.8 |  |  | OR 1.10 (0.50-2.10)‡ |  |
| *[*[*19*](#_ENREF_19)*] Kawai et al (2004)* | | 52 | 284^ | | SZ | Mean  18.9 | Pregnancy | Fewer antenatal care visits |  | |  |  |  | OR 0.88 (0.77, 1.00)‡ |  |
|  |  |  |  |  |  |  |  | High BMI first antenatal visit |  | |  |  |  | OR 1.24 (1.02, 1.50)*‡ |  |
|  |  |  |  |  |  |  |  | High BMI last antenatal visit |  | |  |  |  | OR 1.19 (1.00, 1.41)*‡ |  |
| *[*[*32*](#_ENREF_32)*] Machon et al (1987)* | | 17 | 188 | | SZ | Mean 24  (18-30) | Birth | Winter birth (HR group only) | 43.75 | | 24.0 |  |  | OR 2.46 (0.89-6.80) |  |
|  |  |  |  |  |  |  |  | Urban birth (HR group only) | 93.8 | | 76.7 |  |  | OR 4.60 (0.62-33.96) |  |
| *[*[*33*](#_ENREF_33)*] McGrath et al (2004)* | | 79 | 8744^ | | SZ | 31 | Pregnancy | Dosage of vitamin D :  - ≥2000 IU/day (♂) |  | |  |  |  | RR 0.23 (0.06-0.95)‡* |  |
|  |  |  |  |  |  |  |  | - ≥2000 IU/day (♀) |  | |  |  |  | RR 0.99 (0.14-7.19)‡ |  |
| *[*[*34*](#_ENREF_34)*] McGrath et al (2010)* | | 430 | 430 | | SZ |  | Birth | Neonatal Vitamin D  - first quintile (vs. fourth) |  | |  |  |  | RR 2.1 (1.3-3.5)* |  |
|  |  |  |  |  |  |  |  | - second quintile (vs. fourth) |  | |  |  |  | RR 2.0 (1.3-3.2)* |  |
|  |  |  |  |  |  |  |  | - third quintile (vs. fourth) |  | |  |  |  | RR 2.1 (1.3-3.4)* |  |
|  |  |  |  |  |  |  |  | - fifth quintile (vs. fourth) |  | |  |  |  | RR 1.71 (1.04-2.8)* |  |
| *[*[*35*](#_ENREF_35)*] Perrin (2007)* | | 70 | 7710 | | SSD | 30-38 | Pre-pregnancy | Maternal BMI:  - Low (<19.9 kg/m2) | 23.0 | | 23.0 |  |  | OR 1.00 (0.57-1.75) |  |
|  |  |  |  |  |  |  |  | - Average (20-26.9 kg/m^2^) | 60.0 | | 68.0 |  |  | OR 0.71 (0.44-1.14) |  |
|  |  |  |  |  |  |  |  | - Above average (27-29.9 kg/m^2^) | 8.0 | | 8.0 |  |  | OR 1.36 (0.57-3.25) |  |
|  |  |  |  |  |  |  |  | - High (>30kg/m^2^) | 10.0 | | 3.0 |  |  | OR 3.59 (1.63-7.93)** |  |
| *[*[*36*](#_ENREF_36)*] Schaefer et al (2000)* | | 63 | 6570 | | SSD | 30-38 | Pre-pregnancy | High maternal pre-pregnant BMI (≥30kg/m^2^) | 11.1 | | 4.0 |  |  | OR 3.00 (1.35-6.64)* |  |
| *[*[*37*](#_ENREF_37)*] Talati et al (2013)* | | 79 | 654 | | BD |  | Pregnancy | Maternal smoking |  | |  |  |  |  | OR 2.01 (1.48-2.53)*‡ |
|  |  |  |  |  |  |  |  | Maternal alcohol use |  | |  | 42.0 | 33.0 |  | OR 1.57 (0.97-2.56) |
|  |  |  |  |  |  |  |  | Maternal daily caffeine use |  | |  | 66.0 | 58.0 |  | OR 1.38 (0.84-2.25) |
|  |  |  |  |  |  |  |  | - Urinary infection | 3.7 | | 2.4 | 3.2 | 2.8 | OR 1.56 (0.60-4.07) | OR 1.15 (0.38-3.47) |
|  |  |  |  |  |  |  |  | - Haemorrhage | 3.7 | | 1.4 | 4.1 | 3.7 | OR 2.71 (0.86-8.48) | OR 1.11 (0.42-2.94) |
|  |  |  |  |  |  |  |  | - Rhesus antibodies | 2.0 | | 0.34 | 1.8 | 3.7 | OR 5.98 (0.72-49.81) | OR 0.48 (0.14-1.62) |
|  |  |  |  |  |  |  |  | - Abdominal/pelvic X-ray | 7.1 | | 12.2 | 12.0 | 14.3 | OR 0.55 (0.31-0.97)* | OR 0.82 (0.47-1.43) |
|  |  |  |  |  |  |  |  | - Blood transfusion | 3.0 | | 3.0 | 3.2 | 3.7 | OR 1.00 (0.39-2.57) | OR 0.86 (0.31-2.42) |
|  |  |  |  |  |  |  |  | - Pre-eclampsia | 3.4 | | 9.1 | 9.2 | 9.2 | OR 0.35 (0.17-0.74)* | OR 1.00 (0.52-1.92) |
|  |  |  |  |  |  |  |  | - Admission to hospital | 15.5 | | 22.0 | 16.1 | 17.5 | OR 0.65 (0.43-0.99)* | OR 0.90 (0.55-1.50) |
|  |  |  |  |  |  |  |  | - Any pregnancy complication | 24.0 | | 21.6 | 25.3 | 24.9 | OR 1.15 (0.78-1.68) | OR 1.02 (0.66-1.58) |
| **Study citation** | **Sample size (n)** | | | | **Outcome (Diagnosis)** | **Age at assessment**  **(years)** | | **Exposure (Risk factor)** | **Outcome: % with risk factor** | | | | | **Effect size (CI)** | |
|  | **Cases** | | | **Controls** |  | **Outcome** | **Exposure** | **Measure** | **SSD** | **not-SSD** | | **AP** | **not-AP** | **SSD (vs. not-SSD)** | **AP (vs. not-AP)** |
| ***Obstetric complications*** | | | | | | | | | | | | | | | |
| *[*[*6*](#_ENREF_6)*] Bain et al (2000);*  *[*[*7*](#_ENREF_7)*] Kendell et al (2000)* | *296; 217* | | | *296;*  *217* | SZ; AP | 22-26 | Pregnancy | Pregnancy complications:  - Maternal anaemia | 2.0 | | 3.7 | 2.8 | 3.7 | OR 0.53 (0.19-1.46) | OR 0.75 (0.26-2.19) |
| *[*[*6*](#_ENREF_6)*] Bain et al (2000);*  *[*[*7*](#_ENREF_7)*] Kendell et al (2000) continued* | *296; 217* | | | *296; 217* | SZ; AP | 22-26 | Birth | Delivery complications:  - Oxytocics | 22.6 | | 23.6 | 30.0 | 32.7 | OR 0.95 (0.64-1.39) | OR 0.88 (0.59-1.32) |
|  |  |  |  |  |  |  |  | - Artificial rupture of membranes | 40.9 | | 37.5 | 36.9 | 45.2 | OR 1.15 (0.83-1.60) | OR 0.71 (0.48-1.04) |
|  |  |  |  |  |  |  |  | - Abnormal presentation of foetus | 2.0 | | 4.7 | 7.8 | 3.2 | OR 0.41 (0.16-1.10) | OR 2.56 (1.04-6.32)* |
|  |  |  |  |  |  |  |  | - Forceps delivery | 8.4 | | 11.5 | 10.6 | 12.4 | OR 0.71 (0.41-1.22) | OR 0.84 (0.46-1.51) |
|  |  |  |  |  |  |  |  | - Caesarean section | 6.1 | | 6.8 | 10.1 | 6.9 | OR 0.89 (0.46-1.72) | OR 1.52 (0.76-3.01) |
|  |  |  |  |  |  |  |  | - Baby detained in hospital | 9.5 | | 12.8 | 16.1 | 11.5 | OR 0.72 (0.43-1.20) | OR 1.48 (0.85-2.57) |
|  |  |  |  |  |  |  |  | - Non-spontaneous delivery | 14.9 | | 19.6 | 24.0 | 21.7 | OR 0.72 (0.47-1.10) | OR 1.14 (0.73-1.78) |
|  |  |  |  |  |  |  |  | - Small for gestational age | 7.4 | | 8.4 | 5.5 | 6.9 | OR 0.87 (0.48-1.59) | OR 0.79 (0.36-1.72) |
|  |  |  |  |  |  |  |  | - Birthweight <2500g, gestation <37 weeks | 2.7 | | 1.7 | 5.1 | 2.8 | OR 1.60 (0.52-4.95) | OR 1.87 (0.68-5.11) |
|  |  |  |  |  |  |  |  | - Any delivery complication | 22.6 | | 20.6 | 28.1 | 23.5 | OR 1.13 (0.76-1.67) | OR 1.27 (0.83-1.96) |
|  |  |  |  |  |  |  |  | Any puerperium complication | 7.1 | | 7.4 | 5.5 | 6.9 | OR 0.96 (0.51-1.78) | OR 0.79 (0.36-1.72) |
| *[*[*38*](#_ENREF_38)*] Cannon et al (2002)* | 36; 20 | | | 642 | SCHF; Mania | 26 | Birth | High obstetric complications index score |  | |  |  |  | OR 27.83 (14.76-52.46)** | OR 1.37 (0.61-3.08) |
|  |  |  |  |  |  |  |  | Low Apgar score at birth |  | |  |  |  | OR 5.90 (1.1-32.0)*‡ |  |
|  |  |  |  |  |  |  |  | Hypoxia at birth |  | |  |  |  | OR 5.00 (1.5-16.4)*‡ |  |
|  |  |  |  |  |  |  |  | Small for gestational age |  | |  |  |  | OR 2.80 (1.1-6.9)*‡ |  |
| *[*[*39*](#_ENREF_39)*] Cantor-Graae et al (1997)* | 70 | | | 70 | SZ |  | Pregnancy-  Birth | Higher number of obstetric complications |  | |  |  |  | OR 1.77 (0.97-3.25)# |  |
|  |  |  |  |  |  |  | Birth | Smaller head circumference |  | |  |  |  | OR 2.01 (1.09-2.69)*# |  |
| *[*[*40*](#_ENREF_40)*] Clarke et al (2011)* | 189 | | | 189 | SSD | 31-38 | Birth | Low Apgar score (≤8) | 19.2 | | 14.5 |  |  | OR 1.40 (0.81-2.41) |  |
| *[*[*41*](#_ENREF_41)*] Freedman et al (2013)* | 26 | | | 25 | SSD |  | Birth | Birth weight |  | |  |  |  | OR 1.97 (0.72-5.39)# |  |

| **Study citation** | **Sample size (n)** | | | **Outcome (Diagnosis)** | | **Age at assessment**  **(years)** | | | | **Exposure (Risk factor)** | | | **Outcome: % with risk factor** | | | | | | | | **Effect size (CI)** | | | | |
| --- | --- | --- | --- | --- | --- | --- | --- | --- | --- | --- | --- | --- | --- | --- | --- | --- | --- | --- | --- | --- | --- | --- | --- | --- | --- |
|  | **Cases** | **Controls** | |  | | **Outcome** | | **Exposure** | | **Measure** | | | **SSD** | | **not-SSD** | | **AP** | | **not-AP** | | **SSD (vs. not-SSD)** | | **AP (vs. not-AP)** | | |
| *[*[*42*](#_ENREF_42)*] Gunther-Genta et al (1994)* | 42 | 174 | | SZ | |  | | Birth | | Umbilical cord encircling/knotting | | | 38.1 | | 21.8 | |  | |  | | OR 2.21 (1.08-4.53)* | |  | | |
|  |  |  |  |  |  |  |  |  |  | Atypical foetal presentation | | | 11.9 | | 3.4 | |  | |  | | OR 3.84 (1.08-4.53)* | |  | | |
| *[*[*43*](#_ENREF_43)*] Hollister et al (1996)* | 26 | 3492 | | SZ | | 33-35 | | Birth | | Rhesus incompatibility | | | 46.2 | | 30.0 | |  | |  | | OR 2.00 (0.92-4.35) | |  | | |
| *[*[*44*](#_ENREF_44)*] Hultman et al (1997)* | 82 | 164 | | SSD | | Mean 32  (18-45) | | Birth | | Non-optimal score on obstetric factors index:  - High (≥7) | | | 11.0 | | 4.3 | |  | |  | | OR 2.75 (0.99-7.66) | |  | | |
|  |  |  |  |  |  |  |  |  |  | - Medium | | | 51.2 | | 57.5 | |  | |  | | OR 0.78 (0.38-1.60) | |  | | |
|  |  |  |  |  |  |  |  |  |  | - Low | | | 32.9 | | 47.0 | |  | |  | | OR 0.55 (0.32-0.96)* | |  | | |
| *[*[*17*](#_ENREF_17)*] Hultman et al (1999)* | 167; 198 | 835; 990 | | SZ; AP | | 15-21 | | Pregnancy | | Bleeding during pregnancy | | | 3.6 | | 1.6 | | 3.0 | | 2.3 | | OR 3.5 (1.2-10.3)*‡ | | OR 1.1 (0.4-2.9)‡ | | |
|  |  |  |  |  |  |  |  |  |  | Uterine atony | | | 9.0 | | 6.1 | | 8.6 | | 4.1 | | OR 1.4 (0.7-2.6)‡ | | OR 2.2 (1.2-4.1)*‡ | | |
|  |  |  |  |  |  |  |  |  |  | Preterm rupture of membranes | | | 1.2 | | 0.8 | | 1.0 | | 1.2 | | OR 1.51 (0.31-7.37) | | OR 0.83 (0.18-3.77) | | |
|  |  |  |  |  |  |  |  |  |  | Fetopelvic disproportion | | | 1.8 | | 2.8 | | 2.0 | | 2.6 | | OR 0.64 (0.19-2.14) | | OR 0.76 (0.26-2.23) | | |
|  |  |  |  |  |  |  |  | Birth | | Vacuum extraction | | | 9.0 | | 6.8 | | 7.6 | | 5.6 | | OR 1.36 (0.75-2.46) | | OR 1.39 (0.77-2.50) | | |
|  |  |  |  |  |  |  |  |  |  | Caesarean section | | | 5.4 | | 6.1 | | 7.1 | | 7.6 | | OR 0.88 (0.42-1.82) | | OR 0.93 (0.51-1.68) | | |
|  |  |  |  |  |  |  |  |  |  | Traumatic delivery | | | 4.2 | | 3.7 | | 4.6 | | 4.0 | | OR 1.14 (0.49-2.64) | | OR 1.16 (0.55-2.42) | | |
|  |  |  |  |  |  |  |  |  |  | Gestational age:  - 36 weeks | | | 7.2 | | 4.8 | | 8.2 | | 6.7 | | OR 1.54 (0.79-3.00) | | OR 1.24 (0.71-2.19) | | |
|  |  |  |  |  |  |  |  |  |  | - 37-41 weeks | | | 79.5 | | 79.2 | | 81.6 | | 78.7 | | OR 1.02 (0.68-1.54) | | OR 1.20 (0.81-1.77) | | |
|  |  |  |  |  |  |  |  |  |  | - 42 weeks | | | 13.3 | | 16.0 | | 10.2 | | 14.6 | | OR 0.81 (0.50-1.31) | | OR 0.66 (0.41-1.09) | | |
|  |  |  |  |  |  |  |  |  |  | Birth weight:  - <2500g | | | 6.6 | | 3.7 | | 5.6 | | 3.7 | | OR 1.84 (0.91-3.74) | | OR 1.54 (0.77-3.08) | | |
|  |  |  |  |  |  |  |  |  |  | - 2500-4499g | | | 91.6 | | 93.3 | | 92.4 | | 94.0 | | OR 0.78 (0.43-1.44) | | OR 0.78 (0.43-1.40) | | |
|  |  |  |  |  |  |  |  |  |  | - ≥4500g | | | 1.8 | | 2.9 | | 2.0 | | 2.3 | | OR 0.61 (0.18-2.06) | | OR 0.87 (0.29-2.55) | | |
|  |  |  |  |  |  |  |  |  |  | Birth length:  - <49cm | | | 15.1 | | 14.2 | | 16.8 | | 18.1 | | OR 1.07 (0.67-1.71) | | OR 0.91 (0.61-1.37) | | |
|  |  |  |  |  |  |  |  |  |  | - 49-51cm | | | 52.4 | | 50.4 | | 56.6 | | 51.8 | | OR 1.08 (0.78-1.51) | | OR 1.21 (0.89-1.65) | | |
|  |  |  |  |  |  |  |  |  |  | - 52-54cm | | | 28.3 | | 31.2 | | 24.5 | | 27.6 | | OR 0.87 (0.60-1.26) | | OR 0.85 (0.60-1.21) | | |
|  |  |  |  |  |  |  |  |  |  | - >54cm | | | 4.2 | | 4.2 | | 2.0 | | 2.5 | | OR 1.00 (0.44-2.29) | | OR 0.80 (0.27-2.32) | | |
|  |  |  |  |  |  |  |  |  |  | Head circumference:  - ≤31cm | | | 3.7 | | 2.9 | | 5.6 | | 4.7 | | OR 1.29 (0.52-3.16) | | OR 1.20 (0.61-2.36) | | |
|  |  |  |  |  |  |  |  |  |  | - 32-33cm | | | 17.1 | | 18.3 | | 22.2 | | 24.5 | | OR 0.92 (0.59-1.43) | | OR 0.88 (0.61-1.27) | | |
|  |  |  |  |  |  |  |  |  |  | - 34-35cm | | | 45.1 | | 49.3 | | 51.5 | | 46.7 | | OR 0.84 (0.61-1.18) | | OR 1.21 (0.89-1.64) | | |
|  |  |  |  |  |  |  |  |  |  | - ≥36cm | | | 34.2 | | 29.5 | | 20.7 | | 24.1 | | OR 1.24 (0.87-1.77) | | OR 0.82 (0.57-1.19) | | |
| **Study citation** | **Sample size (n)** | | | | **Outcome (Diagnosis)** | | **Age at assessment**  **(years)** | | | | **Exposure (Risk factor)** | **Outcome: % with risk factor** | | | | | | | | **Effect size (CI)** | | | |  |  |
|  | **Cases** | | **Controls** | |  | | **Outcome** | | **Exposure** | | **Measure** | **SSD** | | **not-SSD** | | **AP** | | **not-AP** | | **SSD (vs. not-SSD)** | | **AP (vs. not-AP)** | |  |  |
| *[*[*17*](#_ENREF_17)*] Hultman et al (1999) continued* | 167; 198 | | 835; 990 | | SZ; AP | | 15-21 | | Birth | | Birth weight for gestational age:  - Small (≥2SD below mean) | 7.2 | | 4.9 | | 5.6 | | 4.6 | | OR 1.5 (0.7-3.1)‡ | | OR 1.3 (0.7-2.7)‡ | | |  |
|  |  |  |  |  |  |  |  |  |  |  | - Large (≥2SD above mean) | 2.4 | | 4.1 | | 3.6 | | 3.3 | | OR 0.5 (0.2-1.6)‡ | | OR 1.2 (0.5-2.9)‡ | | |  |
|  |  |  |  |  |  |  |  |  |  |  | Ponderal index: (vs. 6-94 centiles)  - ≤5 centiles | 7.2 | | 4.2 | | 3.5 | | 5.0 | | OR 2.1 (0.8-4.3)‡ | | OR 0.6 (0.3-1.4)‡ | | |  |
|  |  |  |  |  |  |  |  |  |  |  | - ≥95 centiles | 4.8 | | 5.3 | | 5.1 | | 5.1 | | OR 1.0 (0.4-2.2)‡ | | OR 1.0 (0.5-2.2)‡ | | |  |
|  |  |  |  |  |  |  |  |  |  |  | Apgar score at 1 minute  - 0-6 (vs. 7-10) | 6.1 | | 4.9 | | 6.2 | | 3.4 | | OR 0.97 (0.4-2.3)‡ | | OR 1.3 (0.6-2.7)‡ | | |  |
|  |  |  |  |  |  |  |  |  |  |  | Apgar score at 5 minutes  - 0-6 (vs. 7-10) | 2.4 | | 1.1 | | 0 | | 1.4 | | OR 2.21 (0.68-7.23) | | OR 0.17 (0.01-2.88) | | |  |
|  |  |  |  |  |  |  |  |  |  |  | Asphyxia | 12.6 | | 10.9 | | 14.1 | | 9.2 | | OR 1.0 (0.6-1.8)‡ | | OR 1.40 (0.9-2.3)‡ | | |  |
|  |  |  |  |  |  |  |  |  |  |  | Neonatal jaundice | 0.6 | | 1.1 | | 0 | | 0.8 | | OR 0.54 (0.07-4.30) | | OR 0.29 (0.02-5.12) | | |  |
| *[*[*18*](#_ENREF_18)*] Jones et al (1998)* | 70 | | 1074^ | | SZ | | 27-28 | | Pregnancy | | Fever >38°C during pregnancy | 2.9 | | 0.7 | |  | |  | | OR 3.70 (0.70-18.10)‡ | |  | |  |  |
|  |  |  |  |  |  |  |  |  |  |  | Polyhydramnion | 0 | | 0.2 | |  | |  | | OR 2.87 (0.14-59.55) | |  | |  |  |
|  |  |  |  |  |  |  |  |  |  |  | Albuminuria | 8.6 | | 11.6 | |  | |  | | OR 0.80 (0.3-6.6)‡ | |  | |  |  |
|  |  |  |  |  |  |  |  |  |  |  | Oedema and albuminuria | 2.9 | | 6.6 | |  | |  | | OR 0.4 (0.1-1.8)‡ | |  | |  |  |
|  |  |  |  |  |  |  |  |  |  |  | Preeclampsia | 1.4 | | 3.0 | |  | |  | | OR 0.5 (0.1-4.0)‡ | |  | |  |  |
|  |  |  |  |  |  |  |  |  |  |  | Treated threatened abortion | 2.9 | | 2.0 | |  | |  | | OR 1.5 (0.3-6.6)‡ | |  | |  |  |
|  |  |  |  |  |  |  |  |  |  |  | Bleeding during pregnancy | 4.3 | | 4.5 | |  | |  | | OR 0.9 (0.3-3.0)‡ | |  | |  |  |
|  |  |  |  |  |  |  |  |  |  |  | Placenta previa | 0 | | 0.5 | |  | |  | | OR 1.29 (0.07-23.42) | |  | |  |  |
|  |  |  |  |  |  |  |  |  |  |  | Placental abruption | 2.9 | | 0.2 | |  | |  | | OR 11.4 (1.5-85.9)*‡ | |  | |  |  |
|  |  |  |  |  |  |  |  |  | Birth | | Prolapse of umbilical cord | 0 | | 0.3 | |  | |  | | OR 2.04 (0.11-39.47) | |  | |  |  |
|  |  |  |  |  |  |  |  |  |  |  | Induction of labour | 10.0 | | 5.7 | |  | |  | | OR 0.7 (0.2-2.4)‡ | |  | |  |  |
|  |  |  |  |  |  |  |  |  |  |  | Caesarean section | 2.9 | | 4.2 | |  | |  | | OR 0.7 (0.2-2.9)‡ | |  | |  |  |
|  |  |  |  |  |  |  |  |  |  |  | Vacuum or forceps extraction | 2.9 | | 1.7 | |  | |  | | OR 1.8 (0.4-8.1)‡ | |  | |  |  |
|  |  |  |  |  |  |  |  |  |  |  | Cephalopelvic disproportion | 0 | | 1.7 | |  | |  | | OR 0.40 (0.02-6.69) | |  | |  |  |
|  |  |  |  |  |  |  |  |  |  |  | Labour >24 hours | 1.4 | | 6.5 | |  | |  | | OR 0.2 (0.0-1.6)‡ | |  | |  |  |
|  |  |  |  |  |  |  |  |  |  |  | Frontal, breech, or transverse presentation | 5.7 | | 4.5 | |  | |  | | OR 1.3 (0.4-3.8)‡ | |  | |  |  |
|  |  |  |  |  |  |  |  |  |  |  | Foetal heart rate <120 or >160 BPM during delivery | 10.0 | | 5.2 | |  | |  | | OR 2.0 (0.9-4.7)‡ | |  | |  |  |
|  |  |  |  |  |  |  |  |  |  |  | Congenital malformation | 1.5 | | 0.4 | |  | |  | | OR 3.5 (0.4-32.8)‡ | |  | |  |  |

| **Study citation** | **Sample size (n)** | | **Outcome (Diagnosis)** | **Age at assessment**  **(years)** | | **Exposure (Risk factor)** | **Outcome: % with risk factor** | | | | | **Effect size (CI)** | |
| --- | --- | --- | --- | --- | --- | --- | --- | --- | --- | --- | --- | --- | --- |
|  | **Cases** | **Controls** |  | **Outcome** | **Exposure** | **Measure** | **SSD** | **not-SSD** | | **AP** | **not-AP** | **SSD (vs. not-SSD)** | **AP (vs. not-AP)** |
| *[*[*18*](#_ENREF_18)*] Jones et al (1998) continued* | 76 | 10498^ |  |  | Birth | Apgar score <8:  - at 1 minute | 27.1 | | 14.7 |  |  | OR 2.0 (1.1-3.5)*‡ |  |
|  |  |  |  |  |  | - at 15 minutes | 8.6 | | 3.4 |  |  | OR 2.1 (0.8-5.7)‡ |  |
|  |  |  |  |  |  | Low birth weight:  <2500g | 7.9 | | 3.4 |  |  | OR 2.6 (1.1-5.9)*‡ |  |
|  |  |  |  |  |  | <2000g | 3.9 | | 0.7 |  |  | OR 6.2 (1.9-20.3)**‡ |  |
|  |  |  |  |  |  | Low birth weight (<2500g) and gestational age <37 weeks | 5.3 | | 1.6 |  |  | OR 3.4 (1.2-9.4)*‡ |  |
|  |  |  |  |  |  | Placental weight <10^th^ percentile | 16.4 | | 11.1 |  |  | OR 1.7 (0.9-3.2)‡ |  |
|  |  |  |  |  |  | Gestational age <37 weeks | 6.8 | | 4.9 |  |  | OR 1.4 (0.6-3.4)‡ |  |
|  |  |  |  |  |  | Birth weight <10^th^ percentile for gestational age | 9.6 | | 9.3 |  |  | OR 1.0 (0.5-2.3)‡ |  |
| *[*[*19*](#_ENREF_19)*] Kawai et al (2004)* | 52 | 284 | SZ | Mean 18.9 | Birth | Definitive obstetric complications | 21.1 | | 9.3 |  |  | OR 2.82 (0.70-10.41)‡ |  |
| *[*[*45*](#_ENREF_45)*] Laursen et al (2007)* | 13297; 4490 | 36 242^^; 45 049^^ | SZ; BD | 18-32 | Birth | Small for gestational age (lower 10% before week 37; vs. upper 90% before week 37) | 0.1 | | 0.2 | 0.2 | 0.2 | RR 1.22 (0.63-2.35)‡ | RR 5.32 (2.75-10.29)*‡ |
| *[*[*46*](#_ENREF_46)*] Mathiasen et al (2011)* | 4946; 1431 | 1 328 345;  1 324 830 | SZ; Manic episode/BD | 11-33 | Birth | Gestational age:  24-27 weeks (vs. 39-45) | 0.2 | | 0.1 | 0.1 | 0.1 | RR 2.40 (1.08-5.35)*‡ | RR 1.71 (0.24-12.13)‡ |
|  |  |  |  |  |  | 24-36 weeks (vs. 39-45) | 16.4 | | 17.0 | 14.9 | 16.9 | RR 0.99 (0.99-1.28)‡ | RR 1.36 (1.08-1.72)*‡ |
| *[*[*47*](#_ENREF_47)*] Moilanen et al (2010)* | 111 | 10 823 | SZ | 33-35 | Birth | Low birth weight (<2500g; vs. 2500-4499g) | 7.0 | | 3.0 |  |  | OR 2.5 (1.2-5.1)*‡ |  |
|  |  |  |  |  |  | High birth weight (≥4500g; vs. 2500-4499g) | 7.0 | | 3.0 |  |  | OR 2.4 (1.1-4.9)*‡ |  |
|  | 110 | 10 823 |  |  |  | Short birth length (≤46cm; vs. 46-53cm) | 11.0 | | 5.0 |  |  | OR 2.6 (1.1-5.9)*‡ |  |
|  |  |  |  |  |  | Long birth length (≥54cm; vs. 47-53cm) | 10 .0 | | 5.0 |  |  | OR 1.8 (1.0-3.5)‡ |  |

| **Study citation** | **Sample size (n)** | | | | | **Outcome (Diagnosis)** | | **Age at assessment**  **(years)** | | | | **Exposure (Risk factor)** | **Outcome: % with risk factor** | | | | | **Effect size (CI)** | |
| --- | --- | --- | --- | --- | --- | --- | --- | --- | --- | --- | --- | --- | --- | --- | --- | --- | --- | --- | --- |
|  | **Cases** | | | **Controls** | |  | | **Outcome** | | **Exposure** | | **Measure** | **SSD** | **not-SSD** | | **AP** | **not-AP** | **SSD (vs. not-SSD)** | **AP (vs. not-AP)** |
| *[*[*48*](#_ENREF_48)*] Monfils et al (2009)* | 589 | | 303 686 | | | Non-AP (SZ) | | 12-23 | | Birth | | Preterm birth (vs. term birth)  - Boys | 6.0 | | 4.8 |  |  | OR 1.15 (0.71-1.87)‡ |  |
|  |  |  |  |  |  |  |  |  |  |  |  | - Girls | 5.1 | | 4.1 |  |  | OR 1.12 (0.62-2.03)‡ |  |
|  |  |  |  |  |  |  |  |  |  |  |  | Small for gestational age (vs. at gestational age):  - Boys | 7.8 | | 4.8 |  |  | OR 1.35 (0.85-2.14)‡ |  |
|  |  |  |  |  |  |  |  |  |  |  |  | - Girls | 4.7 | | 5.4 |  |  | OR 0.91 (0.51-1.64)‡ |  |
| *[*[*27*](#_ENREF_27)*] Nosarti et al (2012)* | 669; 217 | | | 1 300 853;  1 301 305 | | Non-AP (SZ);  BD | | Mean 23  (17-29) | | Birth | | Gestational age (vs. 37-41 weeks):  - <32 weeks | 0.9 | | 0.3 | 1.8 | 0.4 | HR 2.5 (1.0-6.0)‡ | HR 7.4 (2.7-20.6)*‡ |
|  |  |  |  |  |  |  |  |  |  |  |  | - 32-36 weeks | 5.6 | | 3.7 | 9.2 | 3.7 | HR 1.6 (1.1-2.3)*‡ | HR 2.7 (1.6-4.5)*‡ |
|  |  |  |  |  |  |  |  |  |  |  |  | - ≥42 weeks | 19.0 | | 17.0 | 18.4 | 17.0 | HR 1.0 (1.0-1.3)‡ | HR 1.0 (0.7-1.5)‡ |
|  |  |  |  |  |  |  |  |  |  |  |  | Small for gestational age (< -2 SDs) | 3.9 | | 3.4 | 4.6 | 3.4 | HR 1.0 (0.7-1.5)‡ | HR 1.0 (0.5-2.0)‡ |
|  |  |  |  |  |  |  |  |  |  |  |  | Apgar score (0-3; vs. 7-10) | 0.2 | | 0.2 | 0.1 | 0.2 | HR 0.7 (0.1-4.8)‡ | HR 3.8 (0.9-15.5)‡ |
|  |  |  |  |  |  |  |  |  |  |  |  | Apgar score (4-6; vs. 7-10) | 1.1 | | 0.7 | 0.5 | 0.7 | HR 1.3 (0.6-2.8)‡ | HR 0.5 (0.1-3.6)‡ |
| *[*[*49*](#_ENREF_49)*] Parnas et al (1982)* | 12 | | | 39^ | | SZ | | Mean 24  (18-30) | | Birth | | Premature rupture of membranes | 8.3 | | 23 |  |  | OR 0.30 (0.03-2.69) |  |
|  |  |  |  |  |  |  |  |  |  |  |  | Abnormal foetal position | 25.0 | | 0 |  |  | OR 29.11 (1.38-612.51)* |  |
|  |  |  |  |  |  |  |  |  |  |  |  | Pelvic contractions during delivery | 8.3 | | 2.5 |  |  | OR 3.53 (0.20-62.37) |  |
|  |  |  |  |  |  |  |  |  |  |  |  | Bleeding after delivery | 8.3 | | 0 |  |  | OR 10.27 (0.39-269.85) |  |
|  |  |  |  |  |  |  |  |  |  |  |  | Asphyxia | 8.3 | | 5.1 |  |  | OR 1.68 (0.14-20.48) |  |
|  |  |  |  |  |  |  |  |  |  |  |  | Umbilical cord complications | 16.6 | | 2.5 |  |  | OR 7.76 (0.62-96.53) |  |
|  |  |  |  |  |  |  |  |  |  |  |  | Secondary uterine inertia | 8.3 | | 2.5 |  |  | OR 3.53 (0.20-62.37) |  |
|  |  |  |  |  |  |  |  |  |  |  |  | Forceps used | 16.6 | | 2.5 |  |  | OR 7.76 (0.62-96.53) |  |
|  |  |  |  |  |  |  |  |  |  |  |  | Birth weight >2500g | 16.6 | | 5.1 |  |  | OR 3.70 (0.46-29.80) |  |
|  |  |  |  |  |  |  |  |  |  |  |  | Placental abnormalities | 8.3 | | 2.5 |  |  | OR 3.53 (0.20-62.37) |  |
|  |  |  |  |  |  |  |  |  |  |  |  | Labour >24 hours | 16.6 | | 7.7 |  |  | OR 2.39 (0.35-16.32) |  |
|  |  |  |  |  |  |  |  |  |  |  |  | Labour >48 hours | 8.3 | | 7.7 |  |  | OR 1.08 (0.10-11.55) |  |
| *[*[*50*](#_ENREF_50)*] Preti et al (2000)* | 44 | | | 44 | | SSD | | 18-28 | | Birth | | Small for gestational age | 27.0 | | 25.0 |  |  | OR 1.12 (0.43-2.91) |  |
|  |  |  |  |  |  |  |  |  |  |  |  | Pre-term birth | 13.0 | | 4.0 |  |  | OR 3.32 (0.63-17.43) |  |
|  |  |  |  |  |  |  |  |  |  |  |  | Apgar score <7 at 5 min | 15.0 | | 0.0 |  |  | OR 17.80 (0.98-322) |  |
|  |  |  |  |  |  |  |  |  |  |  |  | Obstetric complications:  - Any definite | 75.0 | | 59.0 |  |  | OR 2.07 (0.83-5.15) |  |
|  |  |  |  |  |  |  |  |  |  |  |  | - ≥1 with clear damaging potential | 34.0 | | 9.0 |  |  | OR 5.17 (1.55-17.21)** |  |
| *[*[*35*](#_ENREF_35)*] Perrin et al (2007)* | 70 | | | 7710 | | SSD | | 31-38 | | Birth | | Birth length |  | |  |  |  | OR 1.04 (0.69-1.61)# |  |
|  |  |  |  |  |  |  |  |  |  |  |  | Birth weight |  | |  |  |  | OR 1.08 (0.70-1.65)# |  |
|  |  |  |  |  |  |  |  |  |  |  |  | Gestational age |  |  |  |  |  | OR 1.41 (0.92-2.14)# |  |
| **Study citation** | | **Sample size (n)** | | | **Outcome (Diagnosis)** | | **Age at assessment**  **(years)** | | | | **Exposure (Risk factor)** | | **Outcome: % with risk factor** | | | | | **Effect size (CI)** | |
|  | | **Cases** | | **Controls** |  | | **Outcome** | | **Exposure** | | **Measure** | | **SSD** | **not-SSD** | | **AP** | **not-AP** | **SSD (vs. not-SSD)** | **AP (vs. not-AP)** |
| *[*[*51*](#_ENREF_51)*] Rosso et al (2000)* | | 80 | | 56 | SZ/SZAff | | 30-37 | | Pregnancy- Birth | | Hypoxia-associated obstetric complications:  - Foetal growth retardation | | 18.8 | | 12.5 |  |  | OR 1.62 (0.61-4.28) |  |
|  |  |  |  |  |  |  |  |  |  |  | - Foetal infection | | 7.5 | | 5.4 |  |  | OR 1.42 (0.34-5.91) |  |
|  |  |  |  |  |  |  |  |  |  |  | - Birth asphyxia | | 13.8 | | 5.4 |  |  | OR 2.80 (0.75-10.52) |  |
| *[*[*28*](#_ENREF_28)*] Sacker et al (1995)* | | 35; 32 | | 16 812 | SZ; AP | | 28 | | Pregnancy | | Bleeding during pregnancy | | 14.7 | | 4.2 | 18.8 | 4.2 | OR 3.01 (1.41-6.42)*‡ | OR 5.28 (2.17-12.86)** |
|  |  |  |  |  |  |  |  |  |  |  | Low maternal weight during pregnancy | | 14.7 | | 5.7 | 6.3 | 5.4 | OR 2.4 (1.31-4.41)‡ | OR 1.18 (0.28-4.91) |
|  |  |  |  |  |  |  |  |  |  |  | <10 antenatal visits | | 31.3 | | 15.9 | 20.0 | 16.0 | OR 2.58 (1.47-4.53)*‡ | OR 1.31 (0.55-3.12) |
|  |  |  |  |  |  |  |  |  | Birth | | Low birth weight (<2500g) | | 5.9 | | 8.9 | 18.8 | 8.9 | OR 0.64 (0.16-2.62) | OR 2.37 (0.97-5.76) |
|  |  |  |  |  |  |  |  |  |  |  | Non-spontaneous delivery | | 4.3 | | 4.3 | 18.5 | 4.3 | OR 1.00 (0.19-5.13) | OR 5.05 (2.06-12.37)** |
|  |  |  |  |  |  |  |  |  |  |  | Gestation <37 weeks | | 56.3 | | 33.4 | 37.5 | 33.3 | OR 2.57 (1.32-5.01)* | OR 1.20 (0.59-2.46) |
| *[*[*24*](#_ENREF_24)*] Suvisaari et al (2012)* | | 29 | | 208^ | SSD | | 37-41 | | Pregnancy | | Oedema | | 46.4 | | 47.5 |  |  | OR 0.96 (0.44-2.08) |  |
|  |  |  |  |  |  |  |  |  |  |  | Medication | | 67.8 | | 54.3 |  |  | OR 1.77 (0.78-4.05) |  |
|  |  |  |  |  |  |  |  |  |  |  | Infection | | 24.1 | | 10.9 |  |  | HR 3.73 (1,27-11.01)*‡ |  |
|  |  |  |  |  |  |  |  |  |  |  | Hypertension | | 17.2 | | 5.4 |  |  | HR 4.10 (1.15-14.59)*‡ |  |
|  |  |  |  |  |  |  |  |  |  |  | Proteinuria | | 10.3 | | 4.5 |  |  | OR 2.44 (0.62-9.54) |  |
|  |  |  |  |  |  |  |  |  |  |  | Bleeding or threatening premature delivery | | 6.9 | | 6.9 |  |  | OR 1.00 (0.22-4.63) |  |
|  |  |  |  |  |  |  |  |  |  |  | Preeclampsia | | 3.7 | | 3.0 |  |  | OR 1.24 (0.15-10.01) |  |
|  |  |  |  |  |  |  |  |  | Birth | | Proteinuria | | 17.2 | | 10.3 |  |  | OR 1.81 (0.62-5.24) |  |
|  |  |  |  |  |  |  |  |  |  |  | Asphyxia | | 3.6 | | 1.5 |  |  | OR 2.45 (0.26-23.29) |  |
|  |  |  |  |  |  |  |  |  |  |  | Placental abnormality | | 75.0 | | 42.4 |  |  | HR 4.09 (1.59-10.05)**‡ |  |
|  |  |  |  |  |  |  |  |  |  |  | Placental abruption | | 0 | | 2.0 |  |  | OR 0.74 (0.04-14.09) |  |
|  |  |  |  |  |  |  |  |  |  |  | Nuchal cord | | 20.7 | | 29.7 |  |  | OR 0.62 (0.24-1.59) |  |
|  |  |  |  |  |  |  |  |  |  |  | Amniotic fluid | | 33.3 | | 23.8 |  |  | OR 1.60 (0.69-3.69) |  |
|  |  |  |  |  |  |  |  |  |  |  | Instrumental delivery | | 13.8 | | 6.3 |  |  | OR 2.38 (0.72-7.86) |  |
|  |  |  |  |  |  |  |  |  |  |  | Congenital malformations | | 3.5 | | 2.0 |  |  | OR 1.78 (0.20-16.13) |  |
|  |  |  |  |  |  |  |  |  |  |  | Neonatal distress | | 6.9 | | 3.9 |  |  | OR 1.83 (0.37-9.03) |  |
|  |  |  |  |  |  |  |  |  |  |  | Neurological status (discharge) | | 6.3 | | 2.9 |  |  | OR 2.25 (0.41-12.36) |  |
| *[*[*37*](#_ENREF_37)*] Talati et al (2013)* | | 79 | | 654 | BD | |  | | Birth | | Premature delivery (<37 weeks) | |  | |  | 5.0 | 6.0 |  | OR 0.84 (0.29-2.42) |
|  |  |  |  |  |  |  |  |  |  |  | Low birth weight (<2500g) | |  | |  | 7.0 | 6.0 |  | OR 1.30 (0.53-3.17) |
| *[*[*52*](#_ENREF_52)*] Tuovinen et al (2012)* | | 142 | | 5264^ | SSD | | ≥25 | | Pregnancy | | Maternal hypertension | | 39.1 | | 28.8 |  |  | HR 1.38 (0.96-1.99) |  |
| *[*[*53*](#_ENREF_53)*] Zornberg et al (2000)* | | 12; 10 | | 671 | SSD; BD | | 18-27 | | Pregnancy- Birth | | Hypoxic-ischemia-related foetal/neonatal complications | | 83.3 | | 24.1 | 40.0 | 24.9 | OR 15.71 (3.41-72.35)** | OR 2.01 (0.56-7.21) |

**Supplementary Table 2: Demographic risk factors**

| **Study citation** | **Sample size (n)** | | **Outcome (Diagnosis)** | **Age at assessment**  **(years)** | | **Exposure (Risk factor)** | **Outcome: % with risk factor** | | | | **Effect size (CI)** | |  |
| --- | --- | --- | --- | --- | --- | --- | --- | --- | --- | --- | --- | --- | --- |
|  | **Cases** | **Controls** |  | **Outcome** | **Exposure** | **Measure** | **SSD** | **not-SSD** | **AP** | **not-AP** | **SSD (vs. not-SSD)** | **AP (vs. not-AP)** | |
| ***Maternal age at birth*** | | | | | | | | | | | | |  |
| *[*[*10*](#_ENREF_10)*] Brown et al (2004)* | 59 | 105 | SSD | 30-38 | Birth | Older maternal age (≥35 years) | 17.0 | 15.0 |  |  | OR 1.16 (0.49-2.76) |  | |
| *[*[*54*](#_ENREF_54)*] Haukka et al (2004)* | 9013 | 12 046 | SZ | 20-46 | Birth | Maternal age (years):  20 | 6.1 | 3.7 |  |  | OR 1.69 (1.49-1.92)** |  | |
|  |  |  |  |  |  | 20-25 | 27.3 | 20.8 |  |  | OR 1.43 (1.34-1.52)** |  | |
|  |  |  |  |  |  | 26-30 | 30.0 | 28.4 |  |  | OR 1.08 (1.02-1.15)* |  | |
|  |  |  |  |  |  | 31-35 | 19.7 | 24.0 |  |  | OR 0.78 (0.73-0.83)** |  | |
|  |  |  |  |  |  | >35 | 17.0 | 23.1 |  |  | OR 0.68 (0.64-0.73)** |  | |
| *[*[*17*](#_ENREF_17)*] Hultman et al (1999)* | 167; 198 | 835; 990 | SZ; AP | 15-21 | Birth | Maternal age:  ≤ 19 years (vs. 20-29) | 6.0 | 7.1 | 5.6 | 6.1 | OR 0.8 (0.4-1.7)‡ | OR 1.0 (0.5-2.0)‡ | |
|  |  |  |  |  |  | ≥30 years (vs. 20-29) | 26.9 | 21.7 | 30.8 | 24.6 | OR 1.2 (0.8-1.8)‡ | OR 1.4 (0.9-2.0)‡ | |
| *[*[*27*](#_ENREF_27)*] Nosarti et al (2012)* | 669; 217 | 130 085;  1 301 305 | Non-AP;  BD | Mean 23 (17-29) | Birth | Maternal age (years):  <17 | 1.3 | 0.3 | 0.0 | 0.3 | OR 4.38 (2.24-8.55)** | OR 0.76 (0.05-12.25) | |
|  |  |  |  |  |  | <19 | 8.4 | 5.1 | 5.1 | 5.1 | OR 1.71 (1.30-2.24)** | OR 1.00 (0.55-1.83) | |
|  |  |  |  |  |  | >30 | 31.5 | 30.1 | 35.0 | 30.1 | OR 1.07 (0.91-1.26) | OR 1.25 (0.95-1.65) | |
|  |  |  |  |  |  | ≥40 | 2.1 | 1.2 | 1.0 | 1.2 | OR 1.77 (1.04-3.00)* | OR 0.83 (0.22-3.17) | |
| *[*[*28*](#_ENREF_28)*] Sacker et al (1995)* | 35;32 | 16 812 | SZ; AP | 28 | Birth | Maternal age >34 years | 14.7 | 12.7 | 25.0 | 12.7 | OR 1.18 (0.46-3.02) | OR 2.29 (1.03-5.11)* | |
| *[*[*25*](#_ENREF_25)*] Talovic et al (1980)* | 15 | 102 | SZ | Mean 25.1 | Birth | Maternal age <26 years | 67.0 | 43.0 |  |  | OR 2.69 (0.86-8.46) |  | |
| ***Paternal age at birth*** | | | | | | | | | | | | |  |
| *[*[*55*](#_ENREF_55)*] Brown et al (2006)* | 68 | 7641 | SSD | 31-38 | Birth | Paternal age (vs. 15-24 years):  25-34 | 50.0 | 51.0 |  |  | RR 1.6 (0.6-4.1)‡ |  |  |
|  |  |  |  |  |  | 35-44 | 30.9 | 25.8 |  |  | RR 2.2 (0.7-7.1)‡ |  |  |
|  |  |  |  |  |  | 45-68 | 7.4 | 4.6 |  |  | RR 2.7 (0.6-13.4)‡ |  |  |
| *[*[*56*](#_ENREF_56)*] Buizer-Voskamp et al (2011)* | 2564; 1121 | 10 256; 4484 | SZ; BD | Mean 36.66-40.71 | Birth | Paternal age (vs. 25-29 years):  <20 | 0.4 | 0.8 | 1.5 | 1.0 | OR 1.47 (0.98-2.21)‡ | OR 1.68 (0.94-3.01)‡ |  |
|  |  |  |  |  |  | 20-24 | 12.6 | 12.4 | 10.4 | 13.0 | OR 1.05 (0.91-1.22)‡ | OR 0.82 (0.65-1.03)‡ |  |
|  |  |  |  |  |  | 30-34 | 29.3 | 30.7 | 32.5 | 29.6 | OR 1.04 (0.94-1.17)‡ | OR 1.12 (0.96-1.32)‡ |  |
|  |  |  |  |  |  | 35-39 | 14.8 | 12.6 | 13.3 | 14.0 | OR 1.24 (1.07-1.42)*‡ | OR 0.99 (0.80-1.22)‡ |  |
|  |  |  |  |  |  | ≥40 | 7.8 | 6.0 | 6.1 | 5.7 | OR 1.27 (1.05-1.53)*‡ | OR 1.14 (0.84-1.55)‡ |  |

| **Study citation** | **Sample size (n)** | | **Outcome (Diagnosis)** | **Age at assessment**  **(years)** | | | | **Exposure (Risk factor)** | | **Outcome: % with risk factor** | | | | | **Effect size (CI)** | | |  |
| --- | --- | --- | --- | --- | --- | --- | --- | --- | --- | --- | --- | --- | --- | --- | --- | --- | --- | --- |
|  | **Cases** | **Controls** |  | **Outcome** | | **Exposure** | | | **Measure** | **SSD** | | **not-SSD** | **AP** | **not-AP** | **SSD (vs. not-SSD)** | **AP (vs. not-AP)** | | |
| *[*[*54*](#_ENREF_54)*] Haukka et al (2004)* | 8160 | 11 033 | SZ | 20-46 | Birth | | Paternal age (vs. <20 years):  20-25 | | | 16.6 | 11.6 | |  |  | OR 1.38 (0.99-1.93)‡ |  |  |  |
|  |  |  |  |  |  |  | 26-30 | | | 29.6 | 24.7 | |  |  | OR 1.43 (1.00-2.04)‡ |  |  |  |
|  |  |  |  |  |  |  | 31-35 | | | 23.0 | 25.1 | |  |  | OR 1.34 (0.92-1.96)‡ |  |  |  |
|  |  |  |  |  |  |  | >35 | | | 29.5 | 37.7 | |  |  | OR 1.33 (0.88-2.02)‡ |  |  |  |
| *[*[*45*](#_ENREF_45)*] Laursen et al (2007)* | 13 297; 4490 | 36 242;  45 049 | SZ; BD | 18-32 | Birth | | Paternal age (vs. 21-25 years):  ≤ 20 years | | | 4.5 | 4.7 | | 3.4 | 4.7 | RR 1.06 (0.96-1.16)‡ | RR 0.89 (0.75-1.07)‡ |  |  |
|  |  |  |  |  |  |  | 26-30 years | | | 30.2 | 32.7 | | 30.2 | 32.2 | RR 1.04 (0.99-1.10)‡ | RR 1.08 (0.98-1.18)‡ |  |  |
|  |  |  |  |  |  |  | 31-35 years | | | 20.0 | 21.6 | | 22.0 | 21.0 | RR 1.12 (1.05-1.19)*‡ | RR 1.21 (1.09-1.34)*‡ |  |  |
|  |  |  |  |  |  |  | 36-40 years | | | 10.3 | 10.7 | | 11.6 | 10.5 | RR 1.17 (1.09-1.27)*‡ | RR 1.25 (1.09-1.42)*‡ |  |  |
|  |  |  |  |  |  |  | 41-45 years | | | 4.8 | 4.5 | | 4.7 | 4.6 | RR 1.27 (1.14-1.40)*‡ | RR 1.14 (0.96-1.36)‡ |  |  |
|  |  |  |  |  |  |  | 46-50 years | | | 1.9 | 1.7 | | 1.9 | 1.7 | RR 1.42 (1.23-1.63)*‡ | RR 1.26 (0.99-1.61)‡ |  |  |
|  |  |  |  |  |  |  | 51-55 years | | | 0.6 | 0.5 | | 0.8 | 0.5 | RR 1.36 (1.08-1.71)*‡ | RR 1.71 (1.21-2.41)*‡ |  |  |
|  |  |  |  |  |  |  | ≥56 years | | | 0.4 | 0.2 | | 0.2 | 0.3 | RR 1.86 (1.39-2.50)*‡ | RR 1.03 (0.53-2.01)‡ |  |  |
| *[*[*57*](#_ENREF_57)*] Menezes et al (2010)* | 493 | 711 496 | BD | 22-29 | Birth | | Paternal age (vs. 21-24 years):  <21 | | |  |  | | 1.8 | 1.7 |  | HR 1.35 (0.67-2.75)‡ |  |  |
|  |  |  |  |  |  |  | 25-29 | | |  |  | | 32.9 | 37.6 |  | HR 1.15 (0.83-1.59)‡ |  |  |
|  |  |  |  |  |  |  | 30-34 | | |  |  | | 33.1 | 30.6 |  | HR 1.41 (0.99-2.00)‡ |  |  |
|  |  |  |  |  |  |  | 35-39 | | |  |  | | 15.0 | 11.5 |  | HR 1.68 (1.09-2.61)*‡ |  |  |
|  |  |  |  |  |  |  | 40-44 | | |  |  | | 5.5 | 3.6 |  | HR 1.85 (1.04-3.30)*‡ |  |  |
|  |  |  |  |  |  |  | 45-49 | | |  |  | | 1.0 | 1.2 |  | HR 1.06 (0.39-2.83)‡ |  |  |
|  |  |  |  |  |  |  | ≥ 50 | | |  |  | | 0.6 | 0.5 |  | HR 1.43 (0.43-4.76)‡ |  |  |
| *[*[*58*](#_ENREF_58)*] Perrin et al (2010)* | 250 (♀);  405 (♂) | 70 124 | SSD | 28-40 |  | | Paternal age (≥ 35):  - mother with SZ (♀) | | | 2.4 | 0.4 | |  |  | RR 4.7 (2.0-10.9)**‡ |  |  |  |
|  |  |  |  |  |  |  | - father with SZ (♀) | | | 1.2 | 0.3 | |  |  | RR 2.7 (0.9-8.6)‡ |  |  |  |
|  |  |  |  |  |  |  | - brother with SZ (♀) | | | 1.6 | 0.4 | |  |  | RR 2.6 (1.0-7.2)‡ |  |  |  |
|  |  |  |  |  |  |  | - sister with SZ (♀) | | | 3.2 | 0.3 | |  |  | RR 8.2 (3.9-17.0)**‡ |  |  |  |
|  |  |  |  |  |  |  | - mother with SZ (♂) | | | 3.5 | 0.4 | |  |  | RR 5.9 (3.3-10.3)**‡ |  |  |  |
|  |  |  |  |  |  |  | - father with SZ (♂) | | | 1.0 | 0.3 | |  |  | RR 2.1 (0.8-5.6)‡ |  |  |  |
|  |  |  |  |  |  |  | - brother with SZ (♂) | | | 2.2 | 0.4 | |  |  | RR 3.3 (1.7-6.6)**‡ |  |  |  |
|  |  |  |  |  |  |  | - sister with SZ (♂) | | | 1.5 | 0.3 | |  |  | RR 3.0 (1.3-6.9)**‡ |  |  |  |

| **Study citation** | **Sample size (n)** | | | **Outcome (Diagnosis)** | | **Age at assessment**  **(years)** | | | | **Exposure (Risk factor)** | | **Outcome: % with risk factor** | | | | **Effect size (CI)** | |  |
| --- | --- | --- | --- | --- | --- | --- | --- | --- | --- | --- | --- | --- | --- | --- | --- | --- | --- | --- |
|  | **Cases** | **Controls** | |  | | **Outcome** | | **Exposure** | | **Measure** | | **SSD** | **not-SSD** | **AP** | **not-AP** | **SSD (vs. not-SSD)** | **AP (vs. not-AP)** |  |
| *[*[*59*](#_ENREF_59)*] Sipos et al (2004)* | 639 | 711 375 | | SZ | | ~25 | | Birth | | Paternal age (vs. 21-24 years):  <21 | |  |  |  |  | HR 1.31 (0.73-2.36)‡ |  |  |
|  |  |  |  |  |  |  |  |  |  | 25-29 | |  |  |  |  | HR 1.26 (0.93-1.69)‡ |  |  |
|  |  |  |  |  |  |  |  |  |  | 30-34 | |  |  |  |  | HR 1.66 (1.18-2.34)*‡ |  |  |
|  |  |  |  |  |  |  |  |  |  | 35-39 | |  |  |  |  | HR 2.32 (1.56-3.44)*‡ |  |  |
|  |  |  |  |  |  |  |  |  |  | 40-44 | |  |  |  |  | HR 2.08 (1.25-3.46)*‡ |  |  |
|  |  |  |  |  |  |  |  |  |  | 45-49 | |  |  |  |  | HR 1.30 (0.56-3.06)‡ |  |  |
|  |  |  |  |  |  |  |  |  |  | ≥50 | |  |  |  |  | HR 4.62 (2.28-9.36)*‡ |  |  |
| ***Parental education*** | | | | | | | | | | | | | | | | | | |
| *[*[*10*](#_ENREF_10)*] Brown et al (2004)* | 59 | | 105 | | SSD | | 30-38 | | Birth | | Maternal education less than high school | 26.0 | 10.0 |  |  | OR 3.16 (1.33-7.50)* |  | |
| *[*[*14*](#_ENREF_14)*] Canetta et al (in press)* | 773 | | 771 | | SZ; SZAFF | |  | |  | | Maternal education (vs. high school graduate:  - <High school | 25.5 | 22.5 |  |  | OR 1.08 (0.85-1.38) |  | |
|  |  |  |  |  |  |  |  |  |  |  | - Bachelor degree | 10.1 | 14.2 |  |  | OR 0.70 (0.51-0.96) |  | |
|  |  |  |  |  |  |  |  |  |  |  | - Master / PhD degree | 5.7 | 6.5 |  |  | OR 0.86 (0.56-1.32) |  | |
| *[*[*27*](#_ENREF_27)*] Nosarti et al (2012)* | 669; 217 | | 1 300 853;  1 301 305 | | Non-AP; BD | | 17-29 (mean 23) | | Birth | | ‘Lower’ maternal education (<2 years of post-compulsory education) | 25.9 | 19.3 | 19.5 | 19.3 | OR 1.46 (1.23-1.74)** | OR 1.01 (0.72-1.42) | |
|  |  |  |  |  |  |  |  |  |  |  | ‘Higher’ maternal education (>4 years of post-compulsory education) | 29.5 | 31.0 | 32.4 | 31.0 | OR 0.93 (0.79-1.10) | OR 1.07 (0.80-1.42) | |
| *[*[*35*](#_ENREF_35)*] Perrin et al (2007)* | 70 | | 7710 | | SSD | | 30-38 | | Birth | | Maternal education:  - <High school | 25.0 | 18.0 |  |  | OR 1.52 (0.88-2.62) |  | |
|  |  |  |  |  |  |  |  |  |  |  | - High school, trade school | 41.0 | 40.0 |  |  | OR 1.04 (0.65-1.68) |  | |
|  |  |  |  |  |  |  |  |  |  |  | - High school, some college | 20.0 | 25.0 |  |  | OR 0.75 (0.42-1.35) |  | |
|  |  |  |  |  |  |  |  |  |  |  | - College graduate | 15.0 | 17.0 |  |  | OR 0.86 (0.45-1.66) |  | |
| *[*[*37*](#_ENREF_37)*] Talati et al (2013)* | 79 | | 654 | | BD | |  | | Pregnancy | | Maternal education:  - <High school |  |  | 22.0 | 18.0 |  | OR 1.27 (0.72-2.25) | |
|  |  |  |  |  |  |  |  |  |  |  | - High school graduate |  |  | 37.0 | 40.0 |  | OR 0.87 (0.54-1.41) | |
|  |  |  |  |  |  |  |  |  |  |  | - Some college or college graduate |  |  | 42.0 | 42.0 |  | OR 0.98 (0.61-1.58) | |
| *[*[*25*](#_ENREF_25)*] Talovic et al (1980)* | 15 | | 102 | | SZ | | Mean 25.1 | | Birth | | Maternal education beyond 8 years | 36.0 | 9.0 |  |  | OR 5.69 (1.62-19.92)* |  | |
| *[*[*60*](#_ENREF_60)*] Werner et al (2007)* | 381 | | 64 616 | | SZ | | 21-33 | | Birth | | Low paternal education (0-8 years vs. 13+) | 34.9 | 27.2 |  |  | OR 1.17 (1.04-1.32)**‡ |  | |
|  |  |  |  |  |  |  |  |  |  |  | Low maternal education (0-8 years vs. 13+) | 43.8 | 33.8 |  |  | OR 1.14 (1.01-1.28)*‡ |  | |

| **Study citation** | **Sample size (n)** | | **Outcome (Diagnosis)** | **Age at assessment**  **(years)** | | **Exposure (Risk factor)** | **Outcome: % with risk factor** | | | | **Effect size (CI)** | |  |
| --- | --- | --- | --- | --- | --- | --- | --- | --- | --- | --- | --- | --- | --- |
|  | **Cases** | **Controls** |  | **Outcome** | **Exposure** | **Measure** | **SSD** | **not-SSD** | **AP** | **not-AP** | **SSD (vs. not-SSD)** | **AP (vs. not-AP)** |  |
| ***Socio-economic status (SES)*** | | | | | | | | | | | | | |
| *[*[*38*](#_ENREF_38)*] Cannon et al (2002)* | 36 | 703^ | SCHF | 26 | 0-15 | Low family SES (lowest 2 of 6 categories; parental occupation) | 47.2 | 9.2 |  |  | OR 8.82 (4.37-17.81)** |  | |
| *[*[*30*](#_ENREF_30)*] Carter et al (2003)* | 33 | 70^ | SZ | 39-42 | 0-14 | Low SES (paternal occupation) |  |  |  |  | OR 1.90 (0.89-4.08)# |  | |
| *[*[*61*](#_ENREF_61)*] Corcoran et al (2009)* | 637 | 88 192 | SSD | 21-33 | Birth | Low SES (lowest of 6 categories vs. first 4 categories; paternal occupation) | 18.4 | 13.2 |  |  | RR 1.4 (1.1-1.7)**‡ |  | |
| *[*[*62*](#_ENREF_62)*] Castle et al (1993)* | 117 | 117 | SSD |  | Birth | Low SES (paternal occupation) |  |  |  |  | OR 1.7 (0.9-3.2) |  | |
| *[*[*63*](#_ENREF_63)*] Hare et al (1972)* | 143 | 437^^ | SZ |  | Birth | Paternal social class I (highest) | 5.6 | 4.6 |  |  | OR 1.2 (0.5-2.8) |  | |
|  |  |  |  |  |  | Paternal social class II | 11.2 | 13.3 |  |  | OR 0.8 (0.5-1.5) |  | |
|  |  |  |  |  |  | Paternal social class III | 58.0 | 51.2 |  |  | OR 1.3 (0.9-1.9) |  | |
|  |  |  |  |  |  | Paternal social class IV | 11.9 | 12.6 |  |  | OR 0.9 (0.5-1.7) |  | |
|  |  |  |  |  |  | Paternal social class V (lowest) | 13.3 | 18.3 |  |  | OR 0.7 (0.4-1.2) |  | |
| *[*[*64*](#_ENREF_64)*] Harrison et al (2001)* | 58 | 58 | SSD |  | Birth | Low paternal occupation (lowest 2 of 6 categories vs. first 2 categories) |  |  |  |  | OR 1.8 (0.6-5.8) |  | |
|  |  |  |  |  |  | Low SES area |  |  |  |  | OR 2.7 (1.2-6.5)* |  | |
|  |  |  |  |  |  | Low combined deprivation score (combination of the factors above) |  |  |  |  | OR 8.1 (2.7-23.9)** |  | |
| *[*[*19*](#_ENREF_19)*] Kawai et al (2004)* | 52 | 284 | SZ | Mean 18.9 | Birth | Low SES (paternal occupation) | 10.9 | 15.9 |  |  | OR 0.65(0.26-1.64) |  | |
| *[*[*65*](#_ENREF_65)*] Koponen et al (2004)* | 100 | 10 791 | SZ | ≤31 | ≤14 | Paternal social class I & II |  |  |  |  | OR 1.12 (0.06-1.90)‡ |  | |
|  |  |  |  |  |  | Paternal social class IV |  |  |  |  | OR 1.18 (0.71-1.97)‡ |  | |
|  |  |  |  |  |  | Paternal social class – farmers |  |  |  |  | OR 0.79 (0.42-1.49)‡ |  | |
| *[*[*66*](#_ENREF_66)*] Makikyro et al (1997)* | 57 | 10 960 | SZ | 27 |  | High SES (social class I; paternal occupation) | 11.8 | 7.2 |  |  | OR 1.72 (0.77-3.87) |  | |
|  |  |  |  | 23 |  |  |  |  |  |  | OR 2.42 (1.19-4.90)* |  | |
| *[*[*67*](#_ENREF_67)*] Mulvany (2001)* | 352 | 352 | SZ |  | Birth | Low paternal social class (vs. high) |  |  |  |  | OR 0.59 (0.40-0.85)** |  | |
| *[*[*68*](#_ENREF_68)*] Seidman et al (2013)* | 45 | 101^ | SZ | ~37 | 7 | Low SES (lowest quartile; head of household education & occupation; income) | 32.6 | 21.7 |  |  | OR 1.75 (0.80-3.82) |  | |
| *[*[*60*](#_ENREF_60)*] Werner et al (2007)* | 520 | 68 794 | SZ |  |  | Low-status paternal occupation | 16.9 | 11.5 |  |  | OR 1.29 (1.02-1.63)*‡ |  | |
|  |  |  |  |  |  | Low-status maternal occupation | 14.2 | 7.1 |  |  | OR 1.99 (1.29-3.05)**‡ |  |  |
|  | 520 | 69 384 |  |  |  | Low SES area | 36.5 | 29.7 |  |  | OR 1.26 (1.05-1.52)*‡ |  |  |

| **Study citation** | **Sample size (n)** | | **Outcome (Diagnosis)** | **Age at assessment**  **(years)** | | **Exposure (Risk factor)** | **Outcome: % with risk factor** | | | | | | **Effect size (CI)** | | | |  |
| --- | --- | --- | --- | --- | --- | --- | --- | --- | --- | --- | --- | --- | --- | --- | --- | --- | --- |
|  | **Cases** | **Controls** |  | **Outcome** | **Exposure** | **Measure** | **SSD** | **not-SSD** | **AP** | **not-AP** | | | **SSD (vs. not-SSD)** | | **AP (vs. not-AP)** | |  |
| *[*[*69*](#_ENREF_69)*] Wicks et al. (2005)* | 4109 | 2 126 267 | SZ |  | 2-11 | Paternal occupation  - unclassified (vs. white collar) |  |  |  |  | | | HR 1.2 (1.0-1.3)*‡ | |  | | |
|  |  |  |  |  |  | - blue collar (vs. white collar) |  |  |  |  | | | HR 1.0 (0.9-1.0) ‡ | |  | | |
|  |  |  |  |  |  | - self-employed (vs. white collar) |  |  |  |  | | | HR 0.9 (0.8-1.0) ‡ | |  | | |
|  |  |  |  |  |  | Rent apartment (vs. own house) |  |  |  |  | | | HR 1.2 (1.2-1.3)*‡ | |  | | |
|  |  |  |  |  |  | Own apartment (vs. own house) |  |  |  |  | | | HR 1.2 (1.1-1.3)*‡ | |  | | |
|  |  |  |  |  |  | Single-parent household |  |  |  |  | | | HR 1.5 (1.4-1.6)*‡ | |  | | |
|  |  |  |  |  |  | Unemployed |  |  |  |  | | | HR 1.4 (1.2-1.5)*‡ | |  | | |
|  |  |  |  |  |  | Household receiving social welfare benefits |  |  |  |  | | | HR 1.5 (1.2-1.9)*‡ | |  | | |
| ***Urbanicity*** | | | | | | | | | | | | | | | | | |
| *[*[*14*](#_ENREF_14)*] Canetta et al (in press)* | 777 | 777 | SZ; SZAFF |  | Birth | Urban birth (vs. rural) | 63.1 | 56.4 |  | |  | | OR 1.39 (1.10-1.75)* | | |  | |
|  |  |  |  |  |  | Semiurban birth (vs. rural) | 12.0 | 12.9 |  | |  | | OR 1.15 (0.82-1.61) | | |  | |
| *[*[*45*](#_ENREF_45)*] Laursen et al (2007)* | 13297; 4490 | 36 242^^; 45 049^^ | SZ; BD | 18-32 | Birth | Urban birth (vs. rural birth) | 28.4 | 21.2 | 20.8 | | 23.4 | | RR 1.94 (1.84-2.04)*‡ | | | RR 1.20 (1.09-1.31)*‡ | |
| *[*[*32*](#_ENREF_32)*] Machon et al (1987)* | 17 | 188 | SZ | Mean 24 (18-30) | Birth | Urban birth (HR group only) | 93.8 | 76.7 |  | |  | | OR 4.60 (0.62-33.96) | | |  | |
| *[*[*70*](#_ENREF_70)*] Marcelis et al (1998)* | 5606 | 36 509^^ | SZ; AP | 25-50 | Birth | Urbanicity (high level of urban exposure) | 55.2 | 51.0 | 48.3 | | 52.7 | | OR 1.18 (1.12-1.25)** | | | OR 0.84 (0.80-0.88)** | |
| ***Migration and ethnicity*** | | | | | | | | | | | | | | | | | |
| *[*[*71*](#_ENREF_71)*] Bresnahan et al (2007)* | 43 | 7723 | SZ | 30-38 | Birth | Maternal ethnicity (African American vs. white) | 60.5 | 31.9 |  | |  | | RR 1.92 (0.86-4.28)‡ |  | | | |
| *[*[*72*](#_ENREF_72)*] Corcoran et al (2009)* | 637 | 88 192 | SSD | 21-33 | Birth | Second-generation immigrant:  - Father only | 13.3 | 14.3 |  | | |  | RR 0.9 (0.7-1.2)‡ |  | | | |
|  |  |  |  |  |  | - Mother only | 12.7 | 12.3 |  |  |  |  | RR 1.0 (0.8-1.3)‡ |  |  |  |  |
|  |  |  |  |  |  | - Both parents | 43.8 | 42.1 |  |  |  |  | RR 0.9 (0.7-1.1)‡ |  |  |  |  |
| *[*[*37*](#_ENREF_37)*] Talati et al (2013)* | 79 | 654 | BD |  | Pregnancy | Maternal ethnicity:  - Caucasian |  |  | 71.0 | | | 59.0 |  | OR 1.70 (1.02-2.83)* | | | |
|  |  |  |  |  |  | - African American |  |  | 23.0 | | | 30.0 |  | OR 0.72 (0.41-1.25) | | | |
|  |  |  |  |  |  | - Other |  |  | 6.0 | | | 12.0 |  | OR 0.49 (0.19-1.25) | | | |

| **Study citation** | **Sample size (n)** | | **Outcome (Diagnosis)** | **Age at assessment**  **(years)** | | **Exposure (Risk factor)** | **Outcome: % with risk factor** | | | | **Effect size (CI)** | |  |
| --- | --- | --- | --- | --- | --- | --- | --- | --- | --- | --- | --- | --- | --- |
|  | **Cases** | **Controls** |  | **Outcome** | **Exposure** | **Measure** | **SSD** | **not-SSD** | **AP** | **not-AP** | **SSD (vs. not-SSD)** | **AP (vs. not-AP)** |  |
| *[*[*35*](#_ENREF_35)*] Perrin et al (2007)* | 70 | 7710 | SSD | 30-38 | Birth | Maternal ethnicity:  - Black | 45.0 | 27.0 |  |  | OR 2.21 (1.38-3.55)** |  |  |
|  |  |  |  |  |  | - White | 49.0 | 65.0 |  |  | OR 0.52 (0.32-0.83)* |  |  |
|  |  |  |  |  |  | - Other | 6.0 | 8.0 |  |  | OR 0.72 (0.27-1.98) |  |  |
| ***Family factors*** | | | | | | | | | | | | |  |
| *[*[*73*](#_ENREF_73)*] Burman et al (1987)* | 17 | 126^ | SZ | 19-30 | Mean 15.1 (9-20) | Unsatisfactory relationship with mother |  |  |  |  | OR 5.56 (2.17-14.22)**# |  | |
|  |  |  |  |  |  | Unsatisfactory relationship with father |  |  |  |  | OR 5.88 (2.29-15.07)**# |  | |
| *[*[*38*](#_ENREF_38)*] Cannon et al (2002)* | 36; 20 | 642^ | SCHF; Mania | 26 | 3 | Atypical mother-child interactions |  |  |  |  | OR 2.65 (1.20-5.60)*‡ | OR 1.70 (0.60-4.90)‡ | |
| *[*[*30*](#_ENREF_30)*] Carter et al (2003)* | 33 | 70^ | SZ | 39-42 | 0-14 | Family instability |  |  |  |  | OR 2.36 (1.10-5.09)*# |  | |
|  |  |  |  |  |  | Paternal conflict |  |  |  |  | OR 2.41 (1.12-5.20)*# |  |  |
|  |  |  |  |  |  | Maternal conflict |  |  |  |  | OR 1.74 (0.81-3.72)# |  |  |
| *[*[*74*](#_ENREF_74)*] Goldstein (1987)* | 16 | 35 | SSD | Mean 30 | Mean 15 | Communication deviance (Parent):  - Low | 6.3 | 31.4 |  |  | OR 0.15 (0.02-1.25) |  | |
|  |  |  |  |  |  | - High | 62.6 | 28.6 |  |  | OR 4.18 (1.20-14.59)* |  |  |
|  | 15 | 37 |  |  |  | Affective Style (Family):  - Benign | 6.7 | 61.1 |  |  | OR 0.05 (0.01-0.38)** |  |  |
|  |  |  |  |  |  | - Negative | 80.0 | 22.2 |  |  | OR 14.02 (3.18-61.82)** |  |  |
| *[*[*75*](#_ENREF_75)*] Schiffman et al (2002)* | 24 | 61^ | SZ | 33-47 | Mean 15.1 (9-20) | Parental relationship (HR group):  - Good | 16.7 | 44.3 |  |  | OR 0.25 (0.08-0.82)* |  |  |
|  |  |  |  |  |  | - Poor | 45.8 | 16.4 |  |  | OR 4.31 (1.51-12.32)* |  |  |

| **Study citation** | **Sample size (n)** | | **Outcome (Diagnosis)** | | **Age at assessment**  **(years)** | | | | **Exposure (Risk factor)** | **Outcome: % with risk factor** | | | | | | **Effect size (CI)** | |
| --- | --- | --- | --- | --- | --- | --- | --- | --- | --- | --- | --- | --- | --- | --- | --- | --- | --- |
|  | **Cases** | **Controls** |  | | **Outcome** | **Exposure** | | | **Measure** | **SSD** | **not-SSD** | **AP** | | **not-AP** | | **SSD (vs. not-SSD)** | **AP (vs. not-AP)** |
| *[*[*76*](#_ENREF_76)*] Walker et al (1981)* | 9 | 9^ | SZ | 19-30 | | 9-20 | | Family factors (HR ♂only):  *Maternal absence:*  - at 1 year of age | |  |  |  |  | | OR 3.77 (0.67-21.29)# | |  |
|  |  |  |  |  |  |  |  | - at 2 years | |  |  |  |  | | OR 5.38 (0.92-31.38)# | |  |
|  |  |  |  |  |  |  |  | - at 3-5 years | |  |  |  |  | | OR 3.34 (0.60-18.68)# | |  |
|  |  |  |  |  |  |  |  | - at 6-10 years | |  |  |  |  | | OR 4.89 (0.85-28.27)# | |  |
|  |  |  |  |  |  |  |  | - at 11-14 years | |  |  |  |  | | OR 1.00 (0.19-5.34)# | |  |
|  |  |  |  |  |  |  |  | *Paternal absence:*  - at 1 year of age | |  |  |  |  | | OR 6.47 (1.09-38.47)*# | |  |
|  |  |  |  |  |  |  |  | - at 2 years | |  |  |  |  | | OR 8.12 (1.33-49.65)*# | |  |
|  |  |  |  |  |  |  |  | - at 3-5 years | |  |  |  |  | | OR 6.92 (1.15-41.51)*# | |  |
|  |  |  |  |  |  |  |  | - at 6-10 years | |  |  |  |  | | OR 8.17 (1.34-49.95)*# | |  |
|  |  |  |  |  |  |  |  | - at 11-14 years | |  |  |  |  | | OR 4.76 (0.83-27.41)# | |  |
|  |  |  |  |  |  |  |  | *Institutional child care:*  - at 1 year of age | |  |  |  |  | | OR 7.21 (1.20-43.43)*# | |  |
|  |  |  |  |  |  |  |  | - at 2 years | |  |  |  |  |  | OR 5.58 (0.95-32.65)# | |  |
|  |  |  |  |  |  |  |  | - at 3-5 years | |  |  |  |  |  | OR 4.42 (0.77-25.75)# | |  |
|  |  |  |  |  |  |  |  | - at 6-10 years | |  |  |  |  |  | OR 4.09 (0.72-23.25)# | |  |
|  |  |  |  |  |  |  |  | - at 11-14 years | |  |  |  |  |  | OR 2.03 (0.37-10.99)# | |  |
|  |  |  |  |  |  |  |  | Poorer home quality | |  |  |  |  |  | OR 0.46 (0.09-2.68)# | |  |
|  |  |  |  |  |  |  |  | Poorer neighbourhood quality | |  |  |  |  |  | OR 1.69 (0.31-9.10)# | |  |
| ***Sibship*** | | | | | | | | | | | | | | | | | |
| *[*[*14*](#_ENREF_14)*] Canetta et al (in press)* | 773 | 773 | SZ; SZAFF | |  | |  | Twinning | | 1.9 | 4.8 |  | |  | | OR 0.31 (0.21-0.72)** |  |
| *[*[*54*](#_ENREF_54)*] Haukka et al (2004)* | 9013 | 12 046 | SZ | | 20-46 | | Birth | Family size (vs. 2):  3 | | 28.3 | 30.1 |  | |  | | OR 1.13 (0.94-1.35)‡ |  |
|  |  |  |  |  |  |  |  | 4 | | 14.2 | 19.7 |  | |  | | OR 1.28 (1.04-1.58)*‡ |  |
|  |  |  |  |  |  |  |  | 5+ | | 10.5 | 20.1 |  | |  | | OR 1.29 (1.02-1.62)*‡ |  |
|  |  |  |  |  |  |  |  | Birth order (vs. first born):  - second born | | 36.2 | 27.1 |  | |  | | OR 0.62 (0.54-0.72)*‡ |  |
|  |  |  |  |  |  |  |  | - third born | | 14.0 | 21.8 |  | |  | | OR 0.54 (0.44-0.67)*‡ |  |
|  |  |  |  |  |  |  |  | - fourth born or higher | | 7.9 | 18.1 |  | |  | | OR 0.50 (0.38-0.67)*‡ |  |

| **Study citation** | **Sample size (n)** | | **Outcome (Diagnosis)** | **Age at assessment**  **(years)** | | | | **Exposure (Risk factor)** | **Outcome: % with risk factor** | | | | **Effect size (CI)** | |
| --- | --- | --- | --- | --- | --- | --- | --- | --- | --- | --- | --- | --- | --- | --- |
|  | **Cases** | **Controls** |  | **Outcome** | **Exposure** | | | **Measure** | **SSD** | **not-SSD** | **AP** | **not-AP** | **SSD (vs. not-SSD)** | **AP (vs. not-AP)** |
| *[*[*17*](#_ENREF_17)*] Hultman et al (1999)* | 167; 198 | 835; 990 | SZ; AP | 15-21 | | Birth | Twin birth | | 1.8 | 0.7 | 2.0 | 2.3 | OR 2.60 (0.64-10.56) | OR 0.87 (0.29-2.55) |
| *[*[*19*](#_ENREF_19)*] Kawai et al (2004)* | 52 | 284 | SZ | Mean  18.9 | | Birth | Birth order:  - first | | 53.2 | 31.5 |  |  | OR 2.48 (1.36-4.52)** |  |
|  |  |  |  |  |  |  | - other | | 46.7 | 68.5 |  |  | OR 0.40 (0.22-0.73)** |  |
| *[*[*77*](#_ENREF_77)*] Riordan et al (2012)* | 3118 | 894 567 | Non-AP | 19-32 | | Birth | Short retrograde birth interval (<18 month) | | 6.1 | 5.1 |  |  | OR 1.21 (1.04-1.40)* |  |
|  |  |  |  |  |  |  | Short anterograde birth interval (<18 month) | | 6.6 | 4.4 |  |  | OR 1.54 (1.33-1.77)** |  |

**Supplementary Table 3: Childhood and adolescent risk factors**

| **Study citation** | **Sample size (n)** | | **Outcome (Diagnosis)** | **Age at assessment**  **(years)** | | **Exposure (Risk factor)** | **Outcome: % with risk factor** | | | | **Effect size (CI)** | |
| --- | --- | --- | --- | --- | --- | --- | --- | --- | --- | --- | --- | --- |
|  | **Cases** | **Controls** |  | **Outcome** | **Exposure** | **Measure** | **SSD** | **not-SSD** | **AP** | **not-AP** | **SSD (vs. not-SSD)** | **AP (vs. not-AP)** |
| ***Childhood illness*** | | | | | | | | | | | | |
| *[*[*78*](#_ENREF_78)*] Dalman et al (2008)* | 2269 | 1 185 284 | Non-AP | Up to 29 | ≤ 12 | Bacterial CNS | 1.0 | 0.8 |  |  | RR 0.9 (0.3-2.4)‡ |  |
|  |  |  |  |  |  | Viral CNS | 0.8 | 0.6 |  |  | RR 1.3 (0.8-2.0)‡ |  |
| *[*[*65*](#_ENREF_65)*] Koponen et al (2004)*  *(update of [*[*79*](#_ENREF_79)*] Rantakallio et al, 1997)* | 100 | 10 691 | SZ | Up to 31 | ≤ 14 | Viral CNS infections | 4.0 | 0.7 |  |  | OR 2.48 (0.88-7.0)‡ |  |
|  |  |  |  |  |  | Perinatal brain damage |  |  |  |  | OR 3.84 (1.52-9.72)*‡ |  |
|  |  |  |  |  |  | Cerebral palsy |  |  |  |  | OR 0.74 (0.12-4.12)‡ |  |
|  |  |  |  |  |  | Childhood epilepsy |  |  |  |  | OR 1.05 (0.33-3.38)‡ |  |
| *[*[*80*](#_ENREF_80)*] Leask et al (2002)* | 23;29 | 12 180 | SZ, AP | 28 | ≤ 11 | Childhood infections:  - Meningitis | 4.0 | 0.6 | 3.0 | 0.6 | OR 7.8 (1.0-59)*‡ | OR 7.7 (1.0-58)*‡ |
|  |  |  |  |  |  | - Tuberculosis | 4.0 | 0.3 | 3.0 | 0.3 | OR 15 (2.0-120)*‡ | OR 12 (1.6-91)*‡ |
|  |  |  |  |  |  | - Measles | 96.0 | 93.0 | 90.0 | 93.0 | OR 1.37 (0.2-10)‡ | OR 1.84 (0.3-14)‡ |
|  |  |  |  |  |  | - Chicken pox | 75.0 | 76.0 | 52.0 | 76.0 | OR 0.61 (0.2-1.6)‡ | OR 0.33 (0.2-0.7)‡ |
|  |  |  |  |  |  | - Mumps | 42.0 | 53.0 | 52.0 | 53.0 | OR 0.65 (0.3-1.5)‡ | OR 1.13 (0.5-2.5)‡ |
|  |  |  |  |  |  | - German measles | 54.0 | 51.0 | 45.0 | 51.0 | OR 0.85 (0.4-2.1)‡ | OR 0.89 (0.4-2.0)‡ |
|  |  |  |  |  |  | - Whooping cough | 29.0 | 19.0 | 10.0 | 19.0 | OR 1.62 (0.6-4.5)‡ | OR 0.60 (0.2-2.0)‡ |
|  |  |  |  |  |  | - Scarlet fever | 4.0 | 6.3 | 10.0 | 6.3 | OR 0.64 (0.009-4.8)‡ | OR 1.27 (0.3-5.4)‡ |
|  |  |  |  |  |  | - Infectious hepatitis | 0.0 | 3.0 | 0 | 3.0 | OR 0.008 (0->1000)‡ | OR 0.006 (0->1000)‡ |
|  |  |  |  |  |  | - Rheumatic fever | 0.0 | 0.4 | 0 | 0.4 | OR 0.06 (0->1000)‡ | OR 0.05 (0->1000)‡ |
| *[*[*81*](#_ENREF_81)*] Orlovska (2014)* | 10 607; 1859 | 27 663;  36 411^^ | SSD; BD | 10-33 |  | Hospital contact for head injury:  - age 0-5 years | 2.1 | 2.1 | 1.5 | 2.2 | OR 0.99 (0.85-1.16) | OR 0.66 (0.45-0.98) |
|  |  |  |  |  |  | - age 6-10 years | 2.3 | 2.2 | 1.5 | 2.2 | OR 1.05 (0.90-1.22) | OR 0.64 (0.44-0.95) |
|  |  |  |  |  |  | - age 11-15 years | 3.1 | 2.6 | 2.2 | 2.8 | OR 1.20 (1.05-1.36)* | OR 0.76 (0.55-1.05) |
| *[*[*79*](#_ENREF_79)*] Rantakallio et al (1997)* | 76 | 10 941 | SZ | 27-28 | ≤14 | Childhood central nervous system infections | 5.3 | 1.3 |  |  | OR 4.8 (1.6-14.0)*‡ |  |
| ***Other physical risk factors*** | | | | | | | | | | | | |
| *[*[*82*](#_ENREF_82)*] Niemi et al (2005)* | 11 | 101 | SSD | 35-39 | 7, 10, 14 | Low ponderal index at birth and high BMI at 7 years |  |  |  |  | OR 22.8 (2->100)*‡ |  |
| *[*[*35*](#_ENREF_35)*] Perrin et al (2007)* | 70 | 7710 | SSD | 30-38 | 2.5 | Shorter height |  |  |  |  | OR 2.32 (1.51-3.56)**# |  |
|  |  |  |  |  | 9 |  |  |  |  |  | OR 3.47 (2.26-5.32)**# |  |
|  |  |  |  |  | 2.5 | Higher BMI |  |  |  |  | OR 1.21 (0.79-1.86)# |  |
|  |  |  |  |  | 9 |  |  |  |  |  | OR 1.42 (0.92-2.17)# |  |

| **Study citation** | **Sample size (n)** | | | **Outcome (Diagnosis)** | **Age at assessment**  **(years)** | | **Exposure (Risk factor)** | **Outcome: % with risk factor** | | | | **Effect size (CI)** | |
| --- | --- | --- | --- | --- | --- | --- | --- | --- | --- | --- | --- | --- | --- |
|  | **Cases** | **Controls** | |  | **Outcome** | **Exposure** | **Measure** | **SSD** | **not-SSD** | **AP** | **not-AP** | **SSD (vs. not-SSD)** | **AP (vs. not-AP)** |
| ***Substance misuse*** | | | | | | | | | | | | | |
| *[*[*83*](#_ENREF_83)*] Arseneault et al (2002)* | 25 | | 734 | SCHF | 26 | 15 | Cannabis use by age 15 | 12.0 | 3.5 |  |  | OR 4.50 (1.11-18.21)*‡ |  |
| *[*[*84*](#_ENREF_84)*] Welham et al (2009)* | 56 | | 3517 | Non-AP | Mean 21 | Mean 14 | Cannabis use by age 14 | 32.0 | 12.0 |  |  | OR 3.45 (1.95-6.11)** |  |

**Supplementary Table 4: Childhood antecedents**

| **Study citation** | **Sample size (n)** | | | | **Outcome (Diagnosis)** | | **Age at assessment**  **(years)** | | | | **Exposure (Antecedent factor)** | | **Outcome: % with antecedent** | | | | | | | **Effect size (CI)** | | | |
| --- | --- | --- | --- | --- | --- | --- | --- | --- | --- | --- | --- | --- | --- | --- | --- | --- | --- | --- | --- | --- | --- | --- | --- |
|  | **Cases** | | **Controls** | |  | | **Outcome** | | **Exposure** | | **Measure** | | **SSD** | **not-SSD** | | **AP** | | **not-AP** | | **SSD (vs. not-SSD)** | | **AP (vs. not-AP)** | |
| ***Social, emotional, and behavioural functioning, and psychosis symptoms*** | | | | | | | | | | | | | | | | | | | | | | | |
| ***Early childhood (0-5 years)*** | | | | | | | | | | | | | | | | | | | | | | | |
| *[*[*85*](#_ENREF_85)*] Bearden et al (2000)* | 49 | | 5541^ | | | SZ / SZAff | | 30-37 | | 4 | | Deviant behaviour | 33.0 | | 19.0 | |  | |  | | OR 1.68 (1.14-2.46)**‡ | |  |
|  |  |  |  |  |  |  |  |  |  |  |  | Social maladjustment | 18.4 | | 11.0 | |  | |  | | OR 1.65 (0.76-3.58)‡ | |  |
| *[*[*86*](#_ENREF_86)*] Meyer et al (2009)* | 9 | | 88^^ | | | BD | | 18-28 | | ~4 | | CBCL paediatric bipolar disorder profile (attention problems, aggressive behaviour, anxious/depressed) |  | |  | | 55.6 | | 4.5 | |  | | OR 8.75 (1.89-40.58)**‡ |
| *[*[*84*](#_ENREF_84)*] Welham et al (2009)* | 56 | | 3517 | | | Non-AP | | 21 | | 5 | | Overall psychopathology (CBCL)  - total score (highest quartile; ♂) | 38.0 | | 26.0 | |  | |  | | OR 8.80 (1.11-70.00)*‡ | |  |
|  |  |  |  |  |  |  |  |  |  |  |  | - total score (highest quartile; ♀) | 17.0 | | 24.0 | |  | |  | | OR 0.88 (0.25-3.09)‡ | |  |
| ***Middle childhood (6-12 years)*** | | | | | | | | | | | | | | | | | | | | | | | |
| *[*[*87*](#_ENREF_87)*] Amminger et al (1999)* | 15 | | 170 | | | SZ-related psychoses | | Mean 32.27 | | Mean 9.48  (7-12) | | Behavioural problems |  | |  | |  | |  | | OR 3.41 (1.24-9.38)*# | |  |
| *[*[*85*](#_ENREF_85)*] Bearden et al (2000)* | 48 | | 4907^ | | | SZ / SZAff | | 30-37 | | 7 | | Deviant behaviour | 30.8 | | 18.8 | |  | |  | | OR 1.65 (1.13-2.41)**‡ | |  |
|  |  |  |  |  |  |  |  |  |  |  |  | Social maladjustment | 29.2 | | 13.6 | |  | |  | | OR 2.54 (1.33-4.8)**‡ | |  |
| *[*[*38*](#_ENREF_38)*] Cannon et al (2002)* | 36; 20 | | 642 | | | SCHF; Mania | | 26 | | 5, 7, 9, 11 | | Internalising problems |  | |  | |  | |  | | OR 9.68 (5.20-18.01)**# | | OR 22.05 (9.67-50.27)**# |
|  |  |  |  |  |  |  |  |  |  |  |  | Externalising problems |  | |  | |  | |  | | OR 15.40 (8.24-28.80)**# | | OR 59.89 (25.94-138.27)**# |
|  |  |  |  |  |  |  |  |  |  |  |  | Interpersonal adjustment:  -Social isolation |  | |  | |  | |  | | OR 4.04 (2.19-7.46)**# | | OR 243.45 (103.04-575.17)**# |
|  |  |  |  |  |  |  |  |  |  |  |  | -Peer rejection |  | |  | |  | |  | | OR 66.33 (34.68-126.86)**# | | OR 903.18 (372.18-2191.78)**# |
| *[*[*88*](#_ENREF_88)*] Carlson et al (1993)* | 67 | | 194^ | | | BD | | 18+ | | 7-16 | | Behavioural problems |  | |  | | 23.3 | | 12.9 | |  | | OR 2.05 (1.01-4.15)* |
|  |  |  |  |  |  |  |  |  |  |  |  | Attention problems |  | |  | | 23.3 | | 10.8 | |  | | OR 2.51 (1.21-5.18)* |
| *[*[*89*](#_ENREF_89)*] Crow et al (1995);*  *[*[*90*](#_ENREF_90)*] Done et al (1994)* | 33; 31 | | 1385^ | | | SZ; AP | | 16-28 | | 7 | | Social maladjustment (overreaction -externalising behaviour) |  | |  | |  | |  | | OR 3.58 (1.91-6.72)**# | | OR 1.96 (1.03-3.75)*# |
|  | 30; 31 | | 1378^ | | |  |  |  |  | 11 | |  |  | |  | |  | |  | | OR 4.96 (2.57-9.59)**# | | OR 1.23 (0.65-2.35)# |
|  | 33; 31 | | 1385^ | | |  |  | 16-28 | | 7 | | Social maladjustment (underreaction-internalising behaviour) |  | |  | |  | |  | | OR 1.83 (0.98-3.42)# | | OR 1.46 (0.76-2.78)# |
|  | 30; 31 | | 1378^ | | |  |  |  |  | 11 | |  |  | |  | |  | |  | | OR 3.11 (1.61-6.00)**# | | OR 1.35 (0.71-2.58)# |
| **Study citation** | | **Sample size (n)** | | | **Outcome (Diagnosis)** | | **Age at assessment**  **(years)** | | | | **Exposure (Antecedent factor)** | | **Outcome: % with antecedent** | | | | | | | | **Effect size (CI)** | | |
|  | | **Cases** | | **Controls** |  | | **Outcome** | | **Exposure** | | **Measure** | | **SSD** | **not-SSD** | | **AP** | | **not-AP** | | | **SSD (vs. not-SSD)** | **AP (vs. not-AP)** | |
| *[*[*91*](#_ENREF_91)*] Ekstrom et al (2006)* | | 24 | | 73^^ | | SSD | | 31-33 | | Mean age 12  (11-13) | | Emotional instability |  | |  | |  | |  | | OR 2.28 (1.04-5.02)*# | |  |
|  |  |  |  |  |  |  |  |  |  |  |  | Aggression |  | |  | |  | |  | | OR 1.72 (0.78-3.77)# | |  |
|  |  |  |  |  |  |  |  |  |  |  |  | Anxiety |  | |  | |  | |  | | OR 1.10 (0.50-2.41)# | |  |
| *[*[*92*](#_ENREF_92)*] Fisher et al (2013)*  *(update of [*[*93*](#_ENREF_93)*] Poulton et al, 2000)* | | 27 | | 762^^ | | SZ | | 38 | | 11 | | None/weak psychotic symptoms | 89.0 | | 96.6 | |  | |  | | OR 0.28 (0.08-1.01) | |  |
|  |  |  |  |  |  |  |  |  |  |  |  | Strong psychotic symptoms | 11.1 | | 3.5 | |  | |  | | OR 3.44 (0.98-12.15)  RR 4.86 (1.37-17.27)*‡ | |  |
| *[*[*94*](#_ENREF_94)*] Niemi et al (2005)* | | 12 | | 167 | | SSD | | 35-39 | | 5-6 | | Problems in social adjustment |  | |  | |  | |  | | OR 8.08 (1.51-43.17)*‡ | |  |
|  |  |  |  |  |  |  |  |  |  | 7, 12, 15, 17 | | Emotional symptoms |  | |  | |  | |  | | OR 2.38 (0.53-10.69)‡ | |  |
|  |  |  |  |  |  |  |  |  |  |  |  | Conduct problems |  | |  | |  | |  | | OR 2.75 (0.47-16.05)‡ | |  |
|  |  |  |  |  |  |  |  |  |  |  |  | Social inhibition |  | |  | |  | |  | | OR 4.11 (0.66-25.65)‡ | |  |
|  |  |  |  |  |  |  |  |  |  |  |  | Attention problems |  | |  | |  | |  | | OR 4.35 (0.35-54.38)‡ | |  |
| *[*[*95*](#_ENREF_95)*] Ott et al (2002)* | | 9 | | 76^ | | SZ-related psychoses | | Mean 32.1 | | Mean 9.5  (7-12) | | Thought disorder:  - Global thought disorder |  | | | |  | | | | OR 7.48 (2.06-27.14)**# | |  |
|  |  |  |  |  |  |  |  |  |  |  |  | - Positive thought disorder |  |  |  |  |  |  |  |  | OR 3.14 (0.89-11.12)# | |  |
|  |  |  |  |  |  |  |  |  |  |  |  | - Negative thought disorder |  |  |  |  |  |  |  |  | OR 4.98 (1.39-17.83)*# | |  |
|  |  |  |  |  |  |  |  |  |  |  |  | Negative symptoms |  |  |  |  |  |  |  |  | OR 4.06 (1.14-14.46)*# | |  |
| *[*[*93*](#_ENREF_93)*] Poulton et al (2000)* | | 25;14 | | 736; 747 | | SCHF; Manic episode | | 26 | | 11 | | Psychotic Symptoms (≥1 Symptom): | 48.0 | | 12.9 | | 7.1 | | 14.2 | | OR 6.23 (2.76-14.06)** | | OR 0.46 (0.06-3.59) |
|  |  |  |  |  |  |  |  |  |  |  |  | - Weak only (1 symptom) | 36.0 | | 11.7 | | 12.6 | | 12.6 | | OR 4.25 (1.82-9.90)** | | OR 1.00 (0.20-4.92) |
|  |  |  |  |  |  |  |  |  |  |  |  | - Strong only (>1 Symptom) | 12.0 | | 1.2 | | 0 | | 1.6 | | OR 11.23 (2.83-44.48)** | | OR 2.04 (0.11-36.08) |
| *[*[*96*](#_ENREF_96)*] Schiffman et al (2004)* | | 16 | | 70^^ | | SSD | | 31-33 | | 11-13 | | Social deficits |  | | | |  | | | | OR 2.71 (0.82-8.95)# | |  |
| ***Early adolescence*** ***(13-15 years)*** | | | | | | | | | | | | | | | | | | | | | | | |
| *[*[*30*](#_ENREF_30)*] Carter et al (2003)* | | 33 | | 70^ | | SZ | | 39-42 | | Mean 15.1  (9-22) | | Disruptive behaviour (HR group) |  | | | |  | | | | OR 3.05 (1.41-6.62)**# | |  |
|  |  |  |  |  |  |  |  |  |  |  |  | Passive behaviour (HR group) |  |  |  |  |  |  |  |  | OR 1.67 (0.78-3.59)# | |  |
| *[*[*97*](#_ENREF_97)*] Cornblatt et al (1999)* | | 6 | | 40 | | SSD | | 30+ | | Mean ages 12, 13.5, 16.5 | | Poor behavioural adjustment (family functioning, peer relationships, school behaviour) |  | |  | |  | |  | | OR 16.44 (3.13-86.31)**# | |  |
| *[*[*98*](#_ENREF_98)*] Kim-Cohen et al (2003)* | | 37; 29 | | 910 | | SCHF; Mania | | 26 | | 11, 13, 15 | | Oppositional defiant/conduct disorder | 40.0 | | 19.0 | | 38.0 | | 19.0 | | OR 2.84 (1.44-5.60)** | | OR 2.5 (1.1-5.4)*‡ |
|  |  |  |  |  |  |  |  |  |  |  |  | Anxiety disorder | 38.0 | | 20.0 | | 33.0 | | 19.0 | | OR 2.5 (1.2-5.1)*‡ | | OR 2.10 (0.95-4.63) |
|  |  |  |  |  |  |  |  |  |  |  |  | Depression | 32.0 | | 6.0 | | 19.0 | | 6.0 | | OR 7.4 (3.5-16.1)*‡ | | OR 3.3 (1.2-9.2)*‡ |
|  |  |  |  |  |  |  |  |  |  |  |  | ADHD | 20.0 | | 5.0 | | 3.0 | | 6.0 | | OR 4.5 (1.8-11.0)*‡ | | OR 0.48 (0.06-4.16) |

| **Study citation** | | **Sample size (n)** | | | | **Outcome (Diagnosis)** | | **Age at assessment**  **(years)** | | | | **Exposure (Antecedent factor)** | | **Outcome: % with antecedent** | | | | | | | | | | | | **Effect size (CI)** | | | | |
| --- | --- | --- | --- | --- | --- | --- | --- | --- | --- | --- | --- | --- | --- | --- | --- | --- | --- | --- | --- | --- | --- | --- | --- | --- | --- | --- | --- | --- | --- | --- |
|  | | **Cases** | | **Controls** | |  | | **Outcome** | | **Exposure** | | **Measure** | | **SSD** | | | **not-SSD** | | | **AP** | | | **not-AP** | | | **SSD (vs. not-SSD)** | | | **AP (vs. not-AP)** | |
| *[*[*99*](#_ENREF_99)*] Olin et al (1998)* | | 32 | | 249 | | SZ | | 39-42 | | Mean 15.1 (9-20) | | Deviant behaviour | | 37.5 | | | 8.8 | | |  | | |  | | | OR 6.22 (2.69-14.39)** | | |  |  |
|  |  |  |  |  |  |  |  |  |  |  |  | Deviant behaviour (high-risk only) | | 36.7 | | | 10.5 | | |  | | |  | | | OR 4.94 (2.17-11.28)** | | |  |  |
| *[*[*100*](#_ENREF_100)*] Parnas et al (1989)* | | 44 | | 131 | | SSD | | Mean 25  (18-30) | | Mean 15  (9-20) | | Behavioural problems:  - Peculiarity | |  | | | | | |  | | | | | | OR 3.08 (1.64-5.78)**# | | |  |  |
|  |  |  |  |  |  |  |  |  |  |  |  | - Introversion | |  |  |  |  |  |  |  |  |  |  |  |  | OR 1.22 (0.65-2.26)# | | |  |  |
|  |  |  |  |  |  |  |  |  |  |  |  | - Paranoid | |  |  |  |  |  |  |  |  |  |  |  |  | OR 1.89 (1.01-3.52)*# | | |  |  |
|  |  |  |  |  |  |  |  |  |  |  |  | - Schizoid class behaviour | |  |  |  |  |  |  |  |  |  |  |  |  | OR 0.76 (0.41-1.41)# | | |  |  |
|  |  |  |  |  |  |  |  |  |  |  |  | - Class-disturbing behaviour | |  |  |  |  |  |  |  |  |  |  |  |  | OR 0.83 (0.45-1.54)# | | |  |  |
| *[*[*101*](#_ENREF_101)*] Reichart et al (2005)* | | 13 | | 53^ | | BD | | Mean 20.8 (15-26) | | 11-21 | | High score: Depression scale | |  | | |  | | |  | | |  | | |  | | | OR 1.12 (1.04-3.85)** |  |
|  |  |  |  |  |  |  |  |  |  |  |  | High score: Hypomania/Biphasic scale | |  | | |  | | |  | | |  | | |  | | | OR 0.44 (0.18-1.05) |  |
| *[*[*102*](#_ENREF_102)*] Stringaris et al (2009)* | | 96 | | 535 | | BD | | Mean 33.2 | | Mean 13.8 | | Irritability | |  | | |  | | |  | | |  | | |  | | | OR 1.02 (0.39-2.69)‡ |  |
| *[*[*103*](#_ENREF_103)*] Ullman et al (2012)* | | 194 | | 21 090^ | | SSD | | 13-14 | | 30-41 | | Behavioural competency (conduct, orderliness, motivation) | |  | | |  | | |  | | |  | | | HR 0.85 (0.74-0.97)*‡ | | |  |  |
| *[*[*84*](#_ENREF_84)*] Welham et al (2009)* | | 56 | | 3517 | | Non-affective psychosis | | Mean  21 | | Mean  14 | | Overall psychopathology:  - Highest quartile on CBCL (♂) | | 54.0 | | | 24.0 | | |  | | |  | | | OR 2.61 (0.91-7.47)‡ | | |  |  |
|  |  |  |  |  |  |  |  |  |  |  |  | - Highest quartile on CBCL (♀) | | 40.0 | | | 25.0 | | |  | | |  | | | OR 2.07 (0.70-6.07)‡ | | |  |  |
|  |  |  |  |  |  |  |  |  |  |  |  | - Highest quartile on YSR (♂) | | 50.0 | | | 25.0 | | |  | | |  | | | OR 3.77 (1.05-13.55)*‡ | | |  |  |
|  |  |  |  |  |  |  |  |  |  |  |  | - Highest quartile on YSR (♀) | | 37.0 | | | 25.0 | | |  | | |  | | | OR 1.49 (0.53-4.20)‡ | | |  |  |
|  |  |  |  |  |  |  |  |  |  |  |  | Psychotic-like thought problems (YSR): | |  | | |  | | |  | | |  | | |  | | |  |  |
|  |  |  |  |  |  |  |  |  |  |  |  | - ‘I see things’ | |  | | |  | | |  | | |  | | | ♂: OR 2.92 (1.13-7.52)*‡  ♀: OR 0.99 (0.34-2.92)‡ | | |  |  |
|  |  |  |  |  |  |  |  |  |  |  |  | - ‘I hear sounds/voices other people think aren’t there’ | |  | | |  | | |  | | |  | | | ♂: OR 5.09 (2.18-11.84)*‡  ♀: OR 2.27 (1.01-5.12)*‡ | | |  |  |
|  |  |  |  |  |  |  |  |  |  |  |  | - ‘I do things other people think are strange’ | |  | | |  | | |  | | |  | | | ♂: OR 1.22 (0.55-2.71)‡  ♀: OR 2.33 (1.02-5.30)‡ | | |  |  |
|  |  |  |  |  |  |  |  |  |  |  |  | - ‘I day-dream a lot’ | |  | | |  | | |  | | |  | | | ♂: OR 4.22 (1.56-11.39)*‡  ♀: OR 1.95 (0.88-4.29)‡ | | |  |  |
|  |  |  |  |  |  |  |  |  |  |  |  | - ‘I feel confused or in a fog | |  | | |  | | |  | | |  | | | ♂: OR 3.12 (1.38-7.04)*‡  ♀: OR 1.37 (0.66-2.86)‡ | | |  |  |
|  |  |  |  |  |  |  |  |  |  |  |  | - ‘I am suspicious’ | |  | | |  | | |  | | |  | | | ♂: OR 0.75 (0.34-1.65)‡  ♀: OR 2.17 (0.96-4.95)‡ | | |  |  |
|  |  |  |  |  |  |  |  |  |  |  |  | - ‘I feel others are out to get me’ | |  | | |  | | |  | | |  | | | ♂: OR 2.39 (1.07-5.35)*‡  ♀: OR 1.32 (0.58-3.01)‡ | | |  |  |
| **Study citation** | **Sample size (n)** | | | | **Outcome (Diagnosis)** | | **Age at assessment**  **(years)** | | | | **Exposure (Antecedent factor)** | | | **Outcome: % with antecedent** | | | | | | | | | | **Effect size (CI)** | | | | | |  |
|  | **Cases** | | **Controls** | |  | | **Outcome** | | **Exposure** | | **Measure** | | | **SSD** | **not-SSD** | | | **AP** | | | **not-AP** | | | **SSD (vs. not-SSD)** | | | **AP (vs. not-AP)** | | |  |
| ***Cognitive functioning*** | | | | | | | | | | | | | | | | | | | | | | | | | | | | | |  |
| ***Early childhood (0-5 years)*** | | | | | | | | | | | | | | | | | | | | | | | | | | | | | |  |
| *[*[*104*](#_ENREF_104)*] Cannon et al (2000)* | 46 | | 5127^ | | | SZ/SZAff | | 30-37 | | 2-5 | | Low IQ (lowest quartile) | 43.0 | | | 26.0 | | |  | | |  | | | OR 2.15 (1.19-3.86)* | | |  | |  |
|  |  | |  | | |  | |  | |  | | High IQ (highest quartile) | 9.0 | | | 23.0 | | |  | | |  | | | OR 0.33 (0.12-0.91)* | | |  | |  |
| ***Middle childhood (6-12 years)*** | | | | | | | | | | | | | | | | | | | | | | | | | | | | | |  |
| *[*[*104*](#_ENREF_104)*] Cannon et al (2000)* | 57 | | 5829^ | | | SZ/SZAff | | 30-37 | | 6-12 | | Low IQ (lowest quartile) | 37.0 | | | 23.0 | | |  | | |  | | | OR 1.97 (1.14-3.38)* | | |  | |  |
|  |  |  |  |  |  |  |  |  |  |  |  | High IQ (highest quartile) | 14.0 | | | 26.0 | | |  | | |  | | | OR 0.46 (0.22-0.98)* | | |  | |  |
| *[*[*38*](#_ENREF_38)*] Cannon et al (2002)* | 36; 20 | | 642 | | | SCHF; Mania | | 26 | | 3, 5, 7, 9, 11 | | Lower IQ score (ages 3-11) |  | | |  | | |  | | |  | | | OR 16.65 (9.05-31.73)**# | | | OR 3.23 (1.44-7.27)**# | |  |
| *[*[*105*](#_ENREF_105)*] Chong et al (2009)* | 273 | | 5601^ | | | SSD | | 18-21  (♂only) | | 7-12 | | Lower academic achievement |  | | |  | | |  | | |  | | | OR 1.91 (1.53-2.38)** | | |  | |  |
| *[*[*97*](#_ENREF_97)*] Cornblatt et al (1999)* | 6 | | 81 | | | SSD | | >30 | | Mean 12 | | Poor visual attention | 66.7 | | | 21.7 | | |  | | |  | | | OR 7.23 (1.22-42.78)* | | |  | |  |
| *[*[*91*](#_ENREF_91)*] Ekstrom et al (2006)* | 24 | | 145^ | | | SSD | | 31-33 | | Mean 12 | | Lower intellectual functioning |  | | |  | | |  | | |  | | | OR 1.22 (0.56-2.67)# | | |  | |  |
| *[*[*106*](#_ENREF_106)*] Jones et al (1994)* | 30 | | 4716 | | | SZ/ SZAff | | 16-43 | | 8 | | Lower score on verbal test |  | | |  | | |  | | |  | | | OR 2.21 (1.15-4.24)* | | |  | |  |
|  |  |  |  |  |  |  |  |  |  |  |  | Lower score on non-verbal test |  |  |  |  |  |  |  |  |  |  |  |  | OR 1.91 (1.00-3.66)* | | |  |  |  |
|  |  |  |  |  |  |  |  |  |  |  |  | Lower score on reading test |  |  |  |  |  |  |  |  |  |  |  |  | OR 1.56 (0.81-2.99) | | |  |  |  |
|  |  |  |  |  |  |  |  |  |  |  |  | Lower score on vocabulary test |  |  |  |  |  |  |  |  |  |  |  |  | OR 1.42 (0.74-2.72) | | |  |  |  |
|  |  |  |  |  |  |  |  |  |  | 11 | | Lower score on arithmetic test |  |  |  |  |  |  |  |  |  |  |  |  | OR 2.12 (1.10-4.06)* | | |  |  |  |
|  |  |  |  |  |  |  |  |  |  |  |  | Lower score on verbal test |  |  |  |  |  |  |  |  |  |  |  |  | OR 1.83 (0.95-3.51) | | |  |  |  |
|  |  |  |  |  |  |  |  |  |  |  |  | Lower score on non-verbal test |  |  |  |  |  |  |  |  |  |  |  |  | OR 1.62 (0.84-3.10) | | |  |  |  |
|  |  |  |  |  |  |  |  |  |  |  |  | Lower score on reading test |  |  |  |  |  |  |  |  |  |  |  |  | OR 1.66 (0.86-3.17) | | |  |  |  |
|  |  | |  | | |  | |  | |  | | Lower score on vocabulary test |  |  |  |  |  |  |  |  |  |  |  |  | OR 1.66 (0.86-3.17) | | |  |  |  |
| *[*[*107*](#_ENREF_107)*] Koenen et al (2009)* | 35; 8 | | 583^ | | | SSD; Mania | | 32 | | 7,9,11 | | Low IQ (lowest quartile) | 28.6 | | | 11.9 | | | 0.0 | | | 12.6 | | | OR 2.97 (1.37-6.44)* | | | OR 0.41 (0.02-7.10) | |  |
|  |  |  |  |  |  |  |  |  |  |  |  | High IQ (highest quartile) | 11.4 | | | 15.0 | | | 50.0 | | | 14.8 | | | OR 0.73 (0.25-2.12) | | | OR 5.76 (1.41-23.45)* | |  |
| *[*[*108*](#_ENREF_108)*] Kremen et al (2010)* | 10 | | 15^ | | | SZ/ SZAff | | ~40 | | 9-11 | | Poor receptive vocabulary |  | | |  | | |  | | |  | | | OR 5.32 (1.16-24.43)*# | | |  | |  |
| *[*[*109*](#_ENREF_109)*] Meyer et al (2004)* | 9 | | 64 | | | BD | | Mean 21.7 | | Mean 11  (8-15) | | Lower WISC  - Full scale IQ |  | | |  | | |  | | |  | | |  | | | OR 2.51 (0.70-8.97)# | |  |
|  |  |  |  |  |  |  |  |  |  |  |  | - Verbal IQ score |  |  |  |  |  |  |  |  |  |  |  |  |  |  |  | OR 1.80 (0.51-6.40)# | |  |
|  |  |  |  |  |  |  |  |  |  |  |  | - Performance IQ score |  |  |  |  |  |  |  |  |  |  |  |  |  |  |  | OR 2.72 (0.76-9.73)# | |  |
| *[*[*94*](#_ENREF_94)*] Niemi et al (2005)* | 12 | | 133 | | | SSD | | 35-39 | | 7, 12, 15, 17 | | Attention problems |  | | | | | |  | | | | | | OR 4.35 (0.35-54.7)‡ | | |  | |  |
| **Study citation** | **Sample size (n)** | | | | **Outcome (Diagnosis)** | | **Age at assessment**  **(years)** | | | | **Exposure (Antecedent factor)** | | **Outcome: % with antecedent** | | | | | | | | | | | **Effect size (CI)** | | | | | |  |
|  | **Cases** | | **Controls** | |  | | **Outcome** | | **Exposure** | | **Measure** | | **SSD** | | **not-SSD** | | | **AP** | | | **not-AP** | | | **SSD (vs. not-SSD)** | | | **AP (vs. not-AP)** | | |  |
| *[*[*110*](#_ENREF_110)*] Niendam et al (2003)* | 32 | | 201^ | | | SZ/ SZAff | | 30-37 | | 7 | | Low IQ:  - Block design |  | | |  | | |  | | |  | | | OR 1.39 (0.71-2.73)# | | |  | |  |
|  |  |  |  |  |  |  |  |  |  |  |  | - Picture arrangement |  | | |  | | |  | | |  | | | OR 3.82 (1.92-7.59)**# | | |  | |  |
|  |  |  |  |  |  |  |  |  |  |  |  | - Digit span |  | | |  | | |  | | |  | | | OR 1.18 (0.60-2.32)# | | |  | |  |
|  |  |  |  |  |  |  |  |  |  |  |  | - Digit symbol coding |  | | |  | | |  | | |  | | | OR 8.16 (4.04-16.48)**# | | |  | |  |
|  |  |  |  |  |  |  |  |  |  |  |  | - Vocabulary |  | | |  | | |  | | |  | | | OR 2.67 (1.35-5.29)**# | | |  | |  |
|  |  |  |  |  |  |  |  |  |  |  |  | - Comprehension |  | | |  | | |  | | |  | | | OR 1.30 ( 0.66-2.57)# | | |  | |  |
|  |  |  |  |  |  |  |  |  |  |  |  | - Information |  | | |  | | |  | | |  | | | OR 1.46 (0.74-2.87)# | | |  | |  |
| *[*[*111*](#_ENREF_111)*] Osler et al (2007)* | 133; 16 | | 6790 | | | SSD; BD | | 16-49 | | 12 | | Poor cognitive function |  | | |  | | |  | | |  | | | HR 1.14 (0.90-1.44)‡ | | | HR 0.83 (0.41-1.67)‡ | |  |
| *[*[*112*](#_ENREF_112)*] Ott et al (1998)* | 13 | | 159 | | | SSD | | Mean 30.17 (sample 1), 22.09 (sample 2); combined in analysis | | 7 | | Low IQ:  - Full-scale IQ |  | | | | | |  | | | | | | OR 4.13 (1.70-10.02)**# | | |  | |  |
|  |  |  |  |  |  |  |  |  |  |  |  | - Verbal IQ |  |  |  |  |  |  |  |  |  |  |  |  | OR 4.41 (1.81-10.71)**# | | |  |  |  |
|  |  |  |  |  |  |  |  |  |  |  |  | - Performance IQ |  |  |  |  |  |  |  |  |  |  |  |  | OR 2.94 (1.21-7.10)*# | | |  |  |  |
|  |  |  |  |  |  |  |  |  |  |  |  | - Picture arrangement |  |  |  |  |  |  |  |  |  |  |  |  | OR 2.77 (1.15-6.70)*# | | |  |  |  |
|  |  |  |  |  |  |  |  |  |  |  |  | - Comprehension |  |  |  |  |  |  |  |  |  |  |  |  | OR 2.31 (0.96-5.57)# | | |  |  |  |
|  |  |  |  |  |  |  |  |  |  |  |  | - Vocabulary |  |  |  |  |  |  |  |  |  |  |  |  | OR 3.20 (1.32-7.75)*# | | |  |  |  |
|  |  |  |  |  |  |  |  |  |  |  |  | - Subtest-Scatter |  |  |  |  |  |  |  |  |  |  |  |  | OR 1.00 (0.42-2.40)# | | |  |  |  |
|  |  |  |  |  |  |  |  |  |  |  |  | - Picture Arrangement- Vocabulary |  |  |  |  |  |  |  |  |  |  |  |  | OR 1.66 (0.69-3.99)# | | |  |  |  |
|  |  |  |  |  |  |  |  |  |  |  |  | - Comprehension-Vocabulary |  |  |  |  |  |  |  |  |  |  |  |  | OR 1.34 (0.56-3.23)# | | |  |  |  |
|  |  |  |  |  |  |  |  |  |  | 9 | | Low IQ:  - Full-scale IQ |  | | | | | |  | | | | | | OR 3.32 (1.18-9.36)*# | | |  | |  |
|  |  |  |  |  |  |  |  |  |  |  |  | - Verbal IQ |  |  |  |  |  |  |  |  |  |  |  |  | OR 2.21 (0.79-6.21)# | | |  |  |  |
|  |  |  |  |  |  |  |  |  |  |  |  | - Performance IQ |  |  |  |  |  |  |  |  |  |  |  |  | OR 3.79 (1.34-10.73)*# | | |  |  |  |
|  |  |  |  |  |  |  |  |  |  |  |  | - Picture arrangement |  |  |  |  |  |  |  |  |  |  |  |  | OR 2.54 (0.90-7.15)# | | |  |  |  |
|  |  |  |  |  |  |  |  |  |  |  |  | - Comprehension |  |  |  |  |  |  |  |  |  |  |  |  | OR 1.54 (0.55-4.31)# | | |  |  |  |
|  |  |  |  |  |  |  |  |  |  |  |  | - Vocabulary |  |  |  |  |  |  |  |  |  |  |  |  | OR 1.32 (0.47-3.69)# | | |  |  |  |
|  |  |  |  |  |  |  |  |  |  |  |  | - Subtest-Scatter |  |  |  |  |  |  |  |  |  |  |  |  | OR 2.96 (1.05-8.34)*# | | |  |  |  |
|  |  |  |  |  |  |  |  |  |  |  |  | - Picture Arrangement- Vocabulary |  |  |  |  |  |  |  |  |  |  |  |  | OR 1.31 (0.47-3.65)# | | |  |  |  |
|  |  |  |  |  |  |  |  |  |  |  |  | - Comprehension-Vocabulary |  |  |  |  |  |  |  |  |  |  |  |  | OR 1.94 (0.69-5.44)# | | |  |  |  |
| *[*[*113*](#_ENREF_113)*] Schulz et al (2014)* | 49 | | 141 206 | | | SZ | | 16-36 | | 7 | | Low IQ (<-2 SD<70) (vs. ±1SD) | 16.3 | | | 3.3 | | |  | | |  | | | OR 7.89 (3.00-20.72)**‡ | | |  | |  |
|  |  |  |  |  |  |  |  |  |  | 11 | | Low IQ (<-2 SD<70) (vs. ±1SD) | 10.0 | | | 0.1 | | |  | | |  | | | OR 9.01 (2.75-29.50)*‡ | | |  | |  |

| **Study citation** | **Sample size (n)** | | **Outcome (Diagnosis)** | | **Age at assessment**  **(years)** | | | | **Exposure (Antecedent factor)** | **Outcome: % with antecedent** | | | | | **Effect size (CI)** | |  |
| --- | --- | --- | --- | --- | --- | --- | --- | --- | --- | --- | --- | --- | --- | --- | --- | --- | --- |
|  | **Cases** | **Controls** |  | | **Outcome** | | | **Exposure** | **Measure** | **SSD** | **not-SSD** | | **AP** | **not-AP** | **SSD (vs. not-SSD)** | **AP (vs. not-AP)** | |
| *[*[*68*](#_ENREF_68)*] Seidman et al (2013)* | 45; 35 | 101^ | | SSD; AP | | ~37 | 7 | | Lower full scale IQ |  | | |  | | OR 4.11 (2.09-8.11)**# | OR 2.41 (1.07-5.45)*# |  |
|  |  |  |  |  |  |  |  |  | Lower academic achievement |  |  |  |  |  | OR 4.56 (2.31-9.02)**# | OR 1.61 (0.72-3.61)# |  |
|  |  |  |  |  |  |  |  |  | Lower verbal ability |  |  |  |  |  | OR 3.92 (1.99-7.72)**# | OR 2.58 (1.14-5.83)*# |  |
|  |  |  |  |  |  |  |  |  | Lower perceptual motor ability |  |  |  |  |  | OR 2.25 (1.16-4.37)*# | OR 1.52 (0.68-3.42)# |  |
|  |  |  |  |  |  |  |  |  | Lower attention and working memory |  |  |  |  |  | OR 5.26 (2.65-10.44)**# | OR 5.59 (2.43-12.89)**# |  |
| *[*[*114*](#_ENREF_114)*] Sorensen et al (2010)* | 32 | 133^ | | SSD | | 31-33 | 10-13 | | Lower total IQ |  | | |  | | OR 2.24 (1.11-4.54)*# |  |  |
|  |  |  |  |  |  |  |  |  | Lower verbal IQ |  | | |  | | OR 2.01 (0.99-4.05)# |  |  |
|  |  |  |  |  |  |  |  |  | Lower performance IQ |  | | |  | | OR 2.09 (1.04-4.24)*# |  |  |
|  |  |  |  |  |  |  |  |  | Lower score on similarities |  | | |  | | OR 2.57 (1.27-5.22)*# |  |  |
|  |  |  |  |  |  |  |  |  | Lower vocabulary ability |  | | |  | | OR 1.51 (0.75-3.04)# |  |  |
|  |  |  |  |  |  |  |  |  | Lower block design ability |  | | |  | | OR 1.28 (0.63-2.58)# |  |  |
|  |  |  |  |  |  |  |  |  | Lower mazes ability |  | | |  | | OR 2.37 (1.17-4.79)*# |  |  |
|  |  |  |  |  |  |  |  |  | Lower object assembly ability |  | | |  | | OR 2.29 (1.13-4.63)*# |  |  |
| ***Early adolescence (13-15 years)*** | | | | | | | | | | | | | | | | |  |
| *[*[*30*](#_ENREF_30)*] Carter et al (2003)* | 33 | 94^ | | SZ | | 39-42 | Mean 15.1 | | Poor performance IQ |  | |  |  |  | OR 0.90 (0.42-1.92)# |  |  |
|  |  |  |  |  |  |  |  |  | Poor verbal IQ |  | |  |  |  | OR 1.19 (0.56-2.54)# |  |  |
| *[*[*115*](#_ENREF_115)*] Isohanni et al (1998)* | 200 | 10 595 | | SSD | | 28 | 14 | | Special school/class below age-appropriate level | 14.6 | | 5.4 |  |  | OR 2.5 (1.2-5.1)*‡ |  |  |
| *[*[*106*](#_ENREF_106)*] Jones et al (1994)* | 30 | 4716 | | SZ/ SZAff | | 16-43 | 15 | | Low score on arithmetic test |  | |  |  |  | OR 2.21 (1.15-4.24)*# |  |  |
|  |  |  |  |  |  |  |  |  | Low score on non-verbal test |  | |  |  |  | OR 2.84 (1.48-5.46)**# |  |  |
|  |  |  |  |  |  |  |  |  | Low score on reading test |  | |  |  |  | OR 1.50 (0.78-2.88)# |  |  |
|  |  |  |  |  |  |  |  |  | Low score on verbal test |  | |  |  |  | OR 2.33 (1.22-4.47)*# |  |  |
| *[*[*116*](#_ENREF_116)*] MacCabe et al (2013)* | 50; 18 | 10 717 | | SZ/ SZAff; BD | |  | 13 | | Lower verbal ability score |  | |  |  |  | OR 0.90 (0.54-1.48)# | OR 0.48 (0.21-1.11)# |  |
|  |  |  |  |  |  |  |  |  | Lower spatial ability score |  | |  |  |  | OR 1.47 (0.89-2.44)# | OR 0.59 (0.26-1.38)# |  |
|  |  |  |  |  |  |  |  |  | Lower inductive ability score |  | |  |  |  | OR 1.13 (0.68-1.87)# | OR 0.93 (0.40-2.15)# |  |
| *[*[*117*](#_ENREF_117)*] Sorensen et al (2006)* | 84 | 127^ | | SSD | | 39-42 | Mean 15.1 (8-20) | | Low IQ:  - Lower coding score |  | |  |  |  | OR 2.34 (1.41-3.89)**# |  |  |
|  |  |  |  |  |  |  |  |  | - Lower object assembly score |  | |  |  |  | OR 1.52 (0.92-2.52)# |  |  |
| *[*[*103*](#_ENREF_103)*] Ullman et al (2012)* | 194 | 21 090 | | SZ | | 30-41 | 13-14 | | Poor academic performance |  | |  |  |  | HR 0.91 (0.79-1.05)‡ |  |  |

| **Study citation** | **Sample size (n)** | | **Outcome (Diagnosis)** | | **Age at assessment**  **(years)** | | | | **Exposure (Antecedent factor)** | | **Outcome: % with antecedent** | | | | | | | **Effect size (CI)** | | | |
| --- | --- | --- | --- | --- | --- | --- | --- | --- | --- | --- | --- | --- | --- | --- | --- | --- | --- | --- | --- | --- | --- |
|  | **Cases** | **Controls** |  | | **Outcome** | | **Exposure** | | **Measure** | | **SSD** | **not-SSD** | | **AP** | | **not-AP** | | **SSD (vs. not-SSD)** | | **AP (vs. not-AP)** | |
| ***Language functioning*** | | | | | | | | | | | | | | | | | | | | | |
| ***Early childhood (0-5 years)*** | | | | | | | | | | | | | | | | | | | | | |
| *[*[*118*](#_ENREF_118)*] Mouridsen et al (2008)* | 44; 7 | 2763 | | SZ; BD | | Mean 35.8  (28.3-46.7) | | Mean 5.5  (2.9-10.6) | | Developmental language disorder | 40.9 | | 16.3 | | 42.9 | | 16.6 | | OR 3.55 (1.93-6.54)** | | OR 3.77 (0.84-16.92) |
| ***Middle childhood (6-12 years)*** | | | | | | | | | | | | | | | | | | | | | |
| *[*[*85*](#_ENREF_85)*] Bearden et al (2000)* | 21 | 2047^ | | SZ/SZAff | | 30-37 | | 7 | | Abnormal speech | 14.0 | | 0.73 | |  | |  | | OR 12.70 (2.46-65.66)*‡ | |  |
|  |  |  |  |  |  |  |  |  |  | Auditory-Vocal Association Test (expressive language and word association ability) | 10.6 | | 4.3 | |  | |  | | OR 0.71 (0.57-0.89)*‡ | |  |
| *[*[*38*](#_ENREF_38)*] Cannon et al (2002)* | 36; 20 | 642 | | SCHF; Mania | | 26 | | 3-9 | | Receptive language |  | |  | |  | |  | | OR 14.01 (7.50-26.17)**# | | OR 1.24 (0.55-2.77)# |
|  |  |  |  |  |  |  |  |  |  | Expressive language |  | |  | |  | |  | | OR 2.08 (1.13-3.83)*# | | OR 3.23 (1.44-7.27)**# |
| *[*[*119*](#_ENREF_119)*] Griffith et al (1980)* | 17 | 190 | | SZ | | 19-30 | | Mean 15.1 (9-16) | | Verbal associative disturbances |  | |  | |  | |  | | OR 0.73 (0.30-1.80)# | |  |
| *[*[*106*](#_ENREF_106)*] Jones et al (1994)* | 30 | 4716 | | SZ/SZAff | | 16-43 | | 6 | | Non-structural speech problems | 11.1 | | 5.1 | |  | |  | | OR 2.2 (0.7-7.3)‡ | |  |
| *[*[*80*](#_ENREF_80)*] Leask et al (2002)* | 24; 29 | 12 180 | | SZ; AP | | 33 | | 7, 11 | | Speech problems | 17.0 | | 5.0 | | 3.0 | | 5.0 | | OR 3.89 (1.34-11.32)* | | OR 0.59 (0.07-4.97) |
| ***Motor functioning and motor milestones*** | | | | | | | | | | | | | | | | | | | | | |
| ***Early childhood (0-5 years)*** | | | | | | | | | | | | | | | | | | | | | |
| *[*[*38*](#_ENREF_38)*] Cannon et al (2002)* | 36; 20 | 642 | | SCHF; Mania | | 26 | | 3 | | Neurological abnormalities (in motility, passive movements, reflexes, facial musculature, strabismus, nystagmus, foot posture, gait) |  | |  | |  | |  | | OR 4.60 (1.92-11.02)*‡ | | OR 0.80 (0.10-6.40)‡ |
| *[*[*40*](#_ENREF_40)*] Clarke et al (2011)* | 189 | 189 | | SSD | | 31-38 | |  | | Delayed motor milestones:  - Sitting unsupported (in 9^th^ month, compared to earlier) | 11.6 | | 8.5 | |  | |  | | OR 3.4 (1.4-8.2)*‡ | |  |
|  |  |  |  |  |  |  |  |  |  |  |  |  |  |  |  | |  | |  |  |  |
|  |  |  |  |  |  |  |  |  |  | - Walking without support (in 12^th^ month, compared to earlier) | 21.7 | | 13.8 | |  | |  | | OR 1.8 (0.5-6.4)‡ | |  |
|  |  |  |  |  |  |  |  |  |  | - Standing without support (in 13^th^ month, compared to earlier) | 4.8 | | 2.1 | |  | |  | | OR 9.0 (1.8-44.2)*‡ | |  |

| **Study citation** | **Sample size (n)** | | **Outcome (Diagnosis)** | | **Age at assessment**  **(years)** | | | | **Exposure (Antecedent factor)** | | **Outcome: % with antecedent** | | | | | | | **Effect size (CI)** | | | |
| --- | --- | --- | --- | --- | --- | --- | --- | --- | --- | --- | --- | --- | --- | --- | --- | --- | --- | --- | --- | --- | --- |
|  | **Cases** | **Controls** |  | | **Outcome** | | **Exposure** | | **Measure** | | **SSD** | **not-SSD** | | **AP** | | **not-AP** | | **SSD (vs. not-SSD)** | | **AP (vs. not-AP)** | |
| *[*[*120*](#_ENREF_120)*] Isohanni et al (2001)* | 100 | 10 905^ | | SZ | | 31 | | 1 | | Neurological Deviance: | 4.6 | | 1.0 | |  | |  | | OR 4.77 (1.84-12.40)** | |  |
|  |  |  |  |  |  |  |  |  |  | - Delayed Standing Unsupported ≥12 months | 30.3 | | 15.6 | |  | |  | | OR 2.35 (1.53-3.61)** | |  |
|  |  |  |  |  |  |  |  |  |  | - Compared to <9 months | 5.6 | | 10.2 | |  | |  | | OR 0.52 (0.22-1.23) | |  |
|  |  |  |  |  |  |  |  |  |  | - Delayed walking unsupported ≥12 months | 46.0 | | 27.8 | |  | |  | | OR 2.21 (1.49-3.29)** | |  |
|  |  |  |  |  |  |  |  |  |  | - Compared to <10 months | 4.6 | | 10.0 | |  | |  | | OR 0.43 (0.17-1.11) | |  |
| *[*[*121*](#_ENREF_121)*] Rosso et al (2000)* | 49 | 5538^ | | SZ/SZAff | | 30-37 | | 4 | | Unusual movements/postural abnormalities | 14.3 | | 4.9 | |  | |  | | OR 3.1 (1.3-7.0)**‡ | |  |
| ***Middle childhood (6-12 years)*** | | | | | | | | | | | | | | | | | | | | | |
| *[*[*122*](#_ENREF_122)*] Cannon et al (1997)* | 24 | 3953 | | SZ | | 43 | | 11 | | Left or inconsistent hand preference | 16.7 | | 9.6 | |  | |  | | OR 1.89 (0.64-5.55) | |  |
|  |  |  | |  | |  | |  | | Left or inconsistent hand throwing | 16.7 | | 12.3 | |  | |  | | OR 1.43 (0.49-4.20) | |  |
|  |  |  | |  | |  | |  | | Left or inconsistent eye dominance | 58.3 | | 39.3 | |  | |  | | OR 2.16 (0.96-4.87) | |  |
| *[*[*38*](#_ENREF_38)*] Cannon et al (2002)* | 36; 20 | 642 | | SCHF; Mania | | 26 | | 3-9 | | Poor motor development |  | |  | |  | |  | | OR 30.85 (16.34-58.24)** | | OR 40.76 (17.74-93.61)** |
| *[*[*123*](#_ENREF_123)*] Erlenmeyer-Kimling et al (2000)* | 12 | 26^ | | SZ-related psychoses | | 19.7-30.7 mean | | Mean 9.29 | | Gross motor skills |  | |  | |  | |  | | OR 1.85 (0.53-6.43) | |  |
| *[*[*80*](#_ENREF_80)*] Leask et al (2002)* | 30; 29 | 12 180 | | SZ; AP | | 33 | | 7,11 | | Neurological soft signs:  - Hand control problem | 10.0 | | 1.0 | | 0 | | 1.0 | | OR 11.00 (3.29-36.74)** | | OR 1.67 (0.10-27.51) |
|  | 29; 28 |  |  |  |  |  |  |  |  | - Co-ordination problem | 14.0 | | 3.0 | | 4.0 | | 3.0 | | OR 5.26 (1.83-15.10)** | | OR 1.35 (0.20-8.94) |
|  | 24; 29 |  |  |  |  |  |  |  |  | - Tics | 8.0 | | 7.0 | | 17.0 | | 7.0 | | OR 1.16 (0.26-5.06) | | OR 2.72 (1.03-7.19)* |
|  |  |  |  |  |  |  |  |  |  | - Twitches | 8.0 | | 2.0 | | 3.0 | | 2.0 | | OR 4.26 (0.97-18.72) | | OR 1.52 (0.18-12.85) |
|  | 22; 25 |  |  |  |  |  |  |  |  | - Neurological problem | 9.0 | | 0.5 | | 4.0 | | 0.5 | | OR 19.68 (4.47-86.61)** | | OR 8.29 (1.10-62.27)* |
|  | 22; 25 |  |  |  |  |  |  |  |  | - Left thrower | 23.0 | | 10.0 | | 12.0 | | 10.0 | | OR 2.69 (0.99-7.27) | | OR 1.23 (0.37-4.11) |
|  | 22; 24 |  |  |  |  |  |  |  |  | - Left kicker | 18.0 | | 11.0 | | 8.0 | | 11.0 | | OR 1.78 (0.60-5.28) | | OR 0.70 (0.16-3.08) |
|  | 24; 28 |  |  |  |  |  |  |  |  | - Left handed | 21.0 | | 10.0 | | 11.0 | | 10.0 | | OR 2.39 (0.89-6.40) | | OR 1.11 (0.34-3.64) |
|  | 22; 25 |  |  |  |  |  |  |  |  | - Left eyed | 32.0 | | 32.0 | | 36.0 | | 32.0 | | OR 1.00 (0.41-2.45) | | OR 1.20 (0.53-2.71) |
|  | 8; 6 |  |  |  |  |  |  |  |  | - Clumsy | 0 | | 12.0 | | 0 | | 12.0 | | OR 0.43 (0.02-7.48) | | OR 0.43 (0.02-7.48) |
|  | 22; 24 |  |  |  |  |  |  |  |  | - Unsteady (right foot) | 14.0 | | 3.0 | | 8.0 | | 3.0 | | OR 5.26 (1.57-17.63)* | | OR 2.81 (0.64-12.33) |
|  |  |  |  |  |  |  |  |  |  | - Unsteady (left foot) | 14.0 | | 4.0 | | 16.0 | | 4.0 | | OR 3.91 (1.17-13.07)* | | OR 4.57 (1.53-13.67)* |
| *[*[*94*](#_ENREF_94)*] Niemi et al (2005)* | 12 | 28^^ | | SSD | | 35-39 | | 7-17 | | Neurological soft signs |  | |  | |  | |  | | OR 4.48 (0.98-20.5)‡ | |  |

| **Study citation** | **Sample size (n)** | | **Outcome (Diagnosis)** | **Age at assessment**  **(years)** | | **Exposure (Antecedent factor)** | **Outcome: % with antecedent** | | | | | **Effect size (CI)** | | | |
| --- | --- | --- | --- | --- | --- | --- | --- | --- | --- | --- | --- | --- | --- | --- | --- |
|  | **Cases** | **Controls** |  | **Outcome** | **Exposure** | **Measure** | **SSD** | **not-SSD** | | **AP** | **not-AP** | **SSD (vs. not-SSD)** | **AP (vs. not-AP)** | | |
| *[*[*121*](#_ENREF_121)*] Rosso et al (2000)* | 65 | 6400^ | SZ/SZAff | 30-37 | 7 | Unusual movements/postural abnormalities | 15.4 | | 4.2 |  |  | OR 4.4 (2.2-8.9)**‡ |  | |  |
|  |  |  |  |  |  | Poor motor co-ordination | 10.6 | | 4.3 |  |  | OR 2.4 (1.1-5.5)*‡ |  |  |  |
| *[*[*124*](#_ENREF_124)*] Schiffman et al (2002)* | 26 | 216^ | SSD | 31-33 | 11-13 | Minor physical anomalies:  - Low (0-2 anomalies) | 23.1 | | 51.2 |  |  | OR 0.29 (0.11-0.74)* |  |  |  |
|  |  |  |  |  |  | - High (3-8 anomalies) | 76.9 | | 48.6 |  |  | OR 3.52 (1.36-9.11)* |  |  |  |
|  |  |  |  |  |  | - High-risk subjects only (high) | 70.6 | | 42.2 |  |  | OR 3.29 (1.36-7.98)* |  |  |  |
| *[*[*96*](#_ENREF_96)*] Schiffman et al (2004)* | 14 | 114^ | SSD | 31-33 | 11-13 | Neuromotor deficits |  | |  |  |  | OR 3.25 (0.98-10.73)# |  |  |  |
| *[*[*125*](#_ENREF_125)*] Schiffman et al (2005)* | 16 | 226 | SSD | 31-33 | 11-13 | Laterality:  - Left or mixed hand preference | 15.4 | | 13.4 |  |  | OR 1.18 (0.29-4.82) |  |  |  |
|  |  |  |  |  |  | - Left or mixed foot preference | 47.8 | | 28.8 |  |  | OR 2.26 (0.81-6.29) |  |  |  |
|  |  |  |  |  |  | - Left or mixed eye dominance | 61.5 | | 42.1 |  |  | OR 2.20 (0.78-6.22) |  |  |  |
|  |  |  |  |  |  | - Any anomalous laterality | 80.8 | | 56.0 |  |  | OR 3.31 (0.93-11.79) |  |  |  |
| *[*[*126*](#_ENREF_126)*] Schiffman et al (2006)* | 26 | 216 | SSD | 31-33 | 11-13 | Ocular alignment abnormalities  - Strabismus scale |  | |  |  |  | OR 2.91 (1.38-6.12)**# |  |  |  |
|  |  |  |  |  |  | - Eye exam scale |  | |  |  |  | OR 2.22 (1.06-4.66)*# |  |  |  |
| *[*[*127*](#_ENREF_127)*] Schiffman et al (2009)* | 32 | 133^ | SSD | 31-33 | 10-13 | Motor coordination:  - Diadochokinesia (right) | 7.0 | | 3.0 |  |  | OR 3.7 (1.6-8.4)*‡ |  |  |  |
|  |  |  |  |  |  | - Diadochokinesia (left) | 33.0 | | 11.0 |  |  | OR 2.5 (1.0-6.3)‡ |  |  |  |
|  |  |  |  |  |  | - Finger opposition (right) | 19.0 | | 8.0 |  |  | OR 1.5 (0.7-3.4)‡ |  |  |  |
|  |  |  |  |  |  | - Finger opposition (left) | 19.0 | | 8.0 |  |  | OR 1.8 (0.8-4.0)‡ |  |  |  |
|  |  |  |  |  |  | - Right index finger and right foot tap | 9.0 | | 3.0 |  |  | OR 2.0 (0.8-4.8)‡ |  |  |  |
|  |  |  |  |  |  | - Right and left index finger and right foot tap | 41.0 | | 17.0 |  |  | OR 2.5 (1.1-5.5)*‡ |  |  |  |
|  |  |  |  |  |  | - Right hand-left hand opens-closes | 6.0 | | 1.0 |  |  | OR 2.2 (1.0-4.8)‡ |  |  |  |

**References to Supplementary Tables 1-4:**

1. Herman DB, Brown AS, Opler MG, Desai M, Malaspina D, Bresnahan M, Schaefer CA, Susser ES: **Does unwantedness of pregnancy predict schizophrenia in the offspring? Findings from a prospective birth cohort study**. *Social Psychiatry and Psychiatric Epidemiology* 2006, **41**(8):605-610.

2. Khashan AS, Abel KM, McNamee R, et al.: **Higher risk of offspring schizophrenia following antenatal maternal exposure to severe adverse life events**. *Archives of General Psychiatry* 2008, **65**(2):146-152.

3. Maki P, Riekki T, Miettunen J, Isohanni M, Jones PB, Murray GK, Veijola J: **Schizophrenia in the offspring of antenatally depressed mothers in the northern Finland 1966 birth cohort: relationship to family history of psychosis**. *American Journal of Psychiatry* 2010, **167**(1):70-77.

4. Niemi LT, Suvisaari JM, Haukka JK, Lonnqvist JK: **Do maternal psychotic symptoms predict offspring's psychotic disorder? Findings from the Helsinki High-Risk Study**. *Psychiatry Research* 2004, **125**(2):105-115.

5. Babulas V, Factor-Litvak P, Goetz R, Schaefer CA, Brown AS: **Prenatal exposure to maternal genital and reproductive infections and adult schizophrenia**. *American Journal of Psychiatry* 2006, **163**(5):927-929.

6. Bain M, Juszczak E, McInneny K, Kendell RE: **Obstetric complications and affective psychoses. Two case-control studies based on structured obstetric records**. *British Journal of Psychiatry* 2000, **176**:523-526.

7. Kendell RE, McInneny K, Juszczak E, Bain M: **Obstetric complications and schizophrenia. Two case-control studies based on structured obstetric records**. *British Journal of Psychiatry* 2000, **176**:516-522.

8. Brown AS, Schaefer CA, Wyatt RJ, Goetz R, Begg MD, Gorman JM, Susser ES: **Maternal exposure to respiratory infections and adult schizophrenia spectrum disorders: a prospective birth cohort study**. *Schizophr Bull* 2000, **26**(2):287-295.

9. Brown AS, Begg MD, Gravenstein S, Schaefer CA, Wyatt RJ, Bresnahan M, Babulas VP, Susser ES: **Serologic evidence of prenatal influenza in the etiology of schizophrenia**. *Archives of General Psychiatry* 2004, **61**(8):774-780.

10. Brown AS, Hooton J, Schaefer CA, Zhang H, Petkova E, Babulas V, Perrin M, Gorman JM, Susser ES: **Elevated maternal interleukin-8 levels and risk of schizophrenia in adult offspring**. *American Journal of Psychiatry* 2004, **161**(5):889-895.

11. Brown AS, Schaefer CA, Quesenberry CP, Jr., Liu L, Babulas VP, Susser ES: **Maternal exposure to toxoplasmosis and risk of schizophrenia in adult offspring**. *American Journal of Psychiatry* 2005, **162**(4):767-773.

12. Brown AS, Schaefer CA, Quesenberry CP, Jr., Shen L, Susser ES: **No evidence of relation between maternal exposure to herpes simplex virus type 2 and risk of schizophrenia?** *American Journal of Psychiatry* 2006, **163**(12):2178-2180.

13. Buka SL, Cannon TD, Torrey EF, Yolken RH: **Maternal Exposure to Herpes Simplex Virus and Risk of Psychosis Among Adult Offspring**. *Biological Psychiatry* 2008, **63**(8):809-815.

14. Canetta S, Sourander A, Surcel HM, Hinkka-Yli-Salomaki S, Leiviska J, Kellendonk C, McKeague IW, Brown AS: **Elevated Maternal C-Reactive Protein and Increased Risk of Schizophrenia in a National Birth Cohort**. *Am J Psychiatry* in press.

15. Canetta SE, Bao Y, Co MDT, Ennis FA, Cruz J, Terajima M, Shen L, Kellendonk C, Schaefer CA, Brown AS: **Serological documentation of maternal influenza exposure and bipolar disorder in adult offspring**. *American Journal of Psychiatry* 2014, **171**(5):557-563.

16. Clarke MC, Tanskanen A, Huttunen M, Whittaker JC, Cannon M: **Evidence for an Interaction Between Familial Liability and Prenatal Exposure to Infection in the Causation of Schizophrenia**. *American Journal of Psychiatry* 2009, **166**(9):1025-1030.

17. Hultman CM, Sparen P, Takei N, Murray RM, Cnattingius S: **Prenatal and perinatal risk factors for schizophrenia, affective psychosis, and reactive psychosis of early onset: Case-control study**. *British Medical Journal* 1999, **318**(7181):421-426.

18. Jones PB, Rantakallio P, Hartikainen AL, Isohanni M, Sipila P: **Schizophrenia as a long-term outcome of pregnancy, delivery, and perinatal complications: a 28-year follow-up of the 1966 north Finland general population birth cohort**. *American Journal of Psychiatry* 1998, **155**(3):355-364.

19. Kawai M, Minabe Y, Takagai S, Ogai M, Matsumoto H, Mori N, Takei N: **Poor maternal care and high maternal body mass index in pregnancy as a risk factor for schizophrenia in offspring**. *Acta Psychiatrica Scandinavica* 2004, **110**(4):257-263.

20. Mortensen PB, Pedersen CB, Hougaard DM, Nørgaard-Petersen B, Mors O, Børglum AD, Yolken RH: **A Danish National Birth Cohort study of maternal HSV-2 antibodies as a risk factor for schizophrenia in their offspring**. *Schizophrenia Research* 2010, **122**(1–3):257-263.

21. Nielsen PR, Laursen TM, Mortensen PB: **Association Between Parental Hospital-Treated Infection and the Risk of Schizophrenia in Adolescence and Early Adulthood**. *Schizophr Bull* 2013, **39**(1):230-237.

22. Parboosing R, Bao Y, Shen L, Schaefer CA, Brown AS: **Gestational influenza and bipolar disorder in adult offspring**. *JAMA Psychiatry* 2013, **70**(7):677-685.

23. Sørensen HJ, Mortensen EL, Reinisch JM, Mednick SA: **Association Between Prenatal Exposure to Bacterial Infection and Risk of Schizophrenia**. *Schizophr Bull* 2009, **35**(3):631-637.

24. Suvisaari JM, Taxell-Lassas V, Pankakoski M, Haukka JK, Lönnqvist JK, Häkkinen LT: **Obstetric Complications as Risk Factors for Schizophrenia Spectrum Psychoses in Offspring of Mothers With Psychotic Disorder**. *Schizophr Bull* 2012, **39**(5):1056-1066.

25. Talovic SA, Mednick SA, Schulsinger F, Falloon IR: **Schizophrenia in high-risk subjects: prognostic maternal characteristics**. *Journal of Abnormal Psychology* 1980, **89**(3):501-504.

26. Kemppainen L, Makikyro T, Jokelainen J, Nieminen P, Jarvelin MR, Isohanni M: **Is grand multiparity associated with offsprings' hospital-treated mental disorders? A 28-year follow-up of the North Finland 1966 birth cohort**. *Social Psychiatry and Psychiatric Epidemiology* 2000, **35**(3):104-108.

27. Nosarti C, Reichenberg A, Murray RM, Cnattingius S, Lambe MP, Yin L, MacCabe J, Rifkin L, Hultman CM: **Preterm birth and psychiatric disorders in young adult life**. *Archives of General Psychiatry* 2012, **69**(6):E1-8.

28. Sacker A, Done DJ, Crow TJ, Golding J: **Antecedents of schizophrenia and affective illness. Obstetric complications**. *British Journal of Psychiatry* 1995, **166**(6):734-741.

29. Bao Y, Ibram G, Blaner WS, Quesenberry CP, Shen L, McKeague IW, Schaefer CA, Susser ES, Brown AS: **Low maternal retinol as a risk factor for schizophrenia in adult offspring**. *Schizophrenia Research* 2012, **137**(1-3):159-165.

30. Carter JW, Schulsinger F, Parnas J, Cannon T, Mednick SA: **A multivariate prediction model of schizophrenia**. *Schizophr Bull* 2003, **28**(4):649-682.

31. Harper KN, Hibbeln JR, Deckelbaum R, Quesenberry CP, Jr., Schaefer CA, Brown AS: **Maternal serum docosahexaenoic acid and schizophrenia spectrum disorders in adult offspring**. *Schizophrenia Research* 2011, **128**(1-3):30-36.

32. Machon RA, Mednick SA, Schulsinger F: **Seasonality, birth complications and schizophrenia in a high risk sample**. *British Journal of Psychiatry* 1987, **151**:122-124.

33. McGrath J, Saari K, Hakko H, Jokelainen J, Jones P, Jarvelin M-R, Chant D, Isohanni M: **Vitamin D supplementation during the first year of life and risk of schizophrenia: a Finnish birth cohort study**. *Schizophrenia Research* 2004, **67**(2-3):237-245.

34. McGrath JJ, Eyles DW, Pedersen CB, Anderson C, Ko P, Burne TH, Norgaard-Pedersen B, Hougaard DM, Mortensen PB: **Neonatal vitamin D status and risk of schizophrenia: a population-based case-control study**. *Arch Gen Psychiatry* 2010, **67**(9):889-894.

35. Perrin MA, Chen H, Sandberg DE, Malaspina D, Brown AS: **Growth trajectory during early life and risk of adult schizophrenia**. *British Journal of Psychiatry* 2007, **191**(6):512-520.

36. Schaefer CA, Brown AS, Wyatt RJ, Kline J, Begg MD, Bresnahan MA, Susser ES: **Maternal Prepregnant Body Mass and Risk of Schizophrenia in Adult Offspring**. *Schizophr Bull* 2000, **26**(2):275-286.

37. Talati A, Bao Y, Kaufman J, Shen L, Schaefer CA, Brown AS: **Maternal smoking during pregnancy and bipolar disorder in offspring**. *American Journal of Psychiatry* 2013, **170**(10):1178-1185.

38. Cannon M, Caspi A, Moffitt TE, Harrington H, Taylor A, Murray RM, Poulton R: **Evidence for early-childhood, pan-developmental impairment specific to schizophreniform disorder: results from a longitudinal birth cohort**. *Archives of General Psychiatry* 2002, **59**(5):449-456.

39. Cantor-Graae E, McNeil TF, Sjöström K, Nordström LG, Rosenlund T: **Maternal demographic correlates of increased history of obstetric complications in schizophrenia**. *Journal of Psychiatric Research* 1997, **31**(3):347-357.

40. Clarke MC, Tanskanen A, Huttunen M, Leon DA, Murray RM, Jones PB, Cannon M: **Increased risk of schizophrenia from additive interaction between infant motor developmental delay and obstetric complications: evidence from a population-based longitudinal study.[Erratum appears in Am J Psychiatry. 2011, 168(12):1345]**. *American Journal of Psychiatry* 2011, **168**(12):1295-1302.

41. Freedman D, Bao Y, Kremen WS, Vinogradov S, McKeague IW, Brown AS: **Birth weight and neurocognition in schizophrenia spectrum disorders**. *Schizophr Bull* 2013, **39**(3):592-600.

42. Gunther-Genta F, Bovet P, Hohlfeld P: **Obstetric complications and schizophrenia. A case-control study**. *British Journal of Psychiatry* 1994, **164**(2):165-170.

43. Hollister J, Laing P, Mednick SA: **Rhesus incompatibility as a risk factor for schizophrenia in male adults**. *Archives of General Psychiatry* 1996, **53**(1):19-24.

44. Hultman CM, Ohman A, Cnattingius S, Wieselgren IM, Lindström LH: **Prenatal and neonatal risk factors for schizophrenia**. *British Journal of Psychiatry* 1997, **170**(2):128-133.

45. Laursen TM, Munk-Olsen T, Nordentoft M, Bo Mortensen P: **A comparison of selected risk factors for unipolar depressive disorder, bipolar affective disorder, schizoaffective disorder, and schizophrenia from a danish population-based cohort**. *Journal of Clinical Psychiatry* 2007, **68**(11):1673-1681.

46. Mathiasen R, Hansen BM, Forman JL, Kessing LV, Greisen G: **The risk of psychiatric disorders in individuals born prematurely in Denmark from 1974 to 1996**. *Acta Paediatrica* 2011, **100**(5):691-699.

47. Moilanen K, Jokelainen J, Jones PB, Hartikainen A-L, Jarvelin M-R, Isohanni M: **Deviant intrauterine growth and risk of schizophrenia: a 34-year follow-up of the Northern Finland 1966 Birth Cohort**. *Schizophrenia Research* 2010, **124**(1-3):223-230.

48. Monfils GW, Josefsson A, Ekholm Selling K, Sydsjö G: **Preterm birth or foetal growth impairment and psychiatric hospitalization in adolescence and early adulthood in a Swedish population-based birth cohort**. *Acta Psychiatrica Scandinavica* 2009, **119**(1):54-61.

49. Parnas J, Schulsinger F, Teasdale TW, Schulsinger H, Feldman PM, Mednick SA: **Perinatal complications and clinical outcome within the schizophrenia spectrum**. *British Journal of Psychiatry* 1982, **140**:416-420.

50. Preti A, Cardascia L, Zen T, Marchetti M, Favaretto G, Miotto P: **Risk for obstetric complications and schizophrenia**. *Psychiatry Research* 2000, **96**(2):127-139.

51. Rosso IM, Cannon TD, Huttunen T, Huttunen MO, Lonnqvist J, Gasperoni TL: **Obstetric risk factors for early-onset schizophrenia in a Finnish birth cohort**. *American Journal of Psychiatry* 2000, **157**(5):801-807.

52. Tuovinen S, Räikkönen K, Pesonen A-K, Lahti M, Heinonen K, Wahlbeck K, Kajantie E, Osmond C, Barker DJP, Eriksson JG: **Hypertensive disorders in pregnancy and risk of severe mental disorders in the offspring in adulthood: The Helsinki Birth Cohort Study**. *Journal of Psychiatric Research* 2012, **46**(3):303-310.

53. Zornberg GL, Buka SL, Tsuang MT: **Hypoxic-ischemia-related fetal/neonatal complications and risk of schizophrenia and other nonaffective psychoses: a 19-year longitudinal study**. *American Journal of Psychiatry* 2000, **157**(2):196-202.

54. Haukka JK, Suvisaari J, Lonnqvist J: **Family structure and risk factors for schizophrenia: case-sibling study**. *BMC Psychiatry* 2004, **4**:41.

55. Brown AS, Schaefer CA, Wyatt RJ, Begg MD, Goetz R, Bresnahan MA, Harkavy-Friedman J, Gorman JM, Malaspina D, Susser ES: **Paternal age and risk of schizophrenia in adult offspring**. *American Journal of Psychiatry* 2006, **159**(9):1528-1533.

56. Buizer-Voskamp JE, Laan W, Staal WG, Hennekam EAM, Aukes MF, Termorshuizen F, Kahn RS, Boks MPM, Ophoff RA: **Paternal age and psychiatric disorders: Findings from a Dutch population registry**. *Schizophrenia Research* 2011, **129**(2–3):128-132.

57. Menezes PR, Lewis G, Rasmussen F, Zammit S, Sipos A, Harrison GL, Tynelius P, Gunnell D: **Paternal and maternal ages at conception and risk of bipolar affective disorder in their offspring**. *Psychol Med* 2010, **40**(03):477-485.

58. Perrin M, Harlap S, Kleinhaus K, Lichtenberg P, Manor O, Draiman B, Fennig S, Malaspina D: **Older paternal age strongly increases the morbidity for schizophrenia in sisters of affected females**. *American Journal of Medical Genetics Part B, Neuropsychiatric Genetics* 2010, **153B**(7):1329-1335.

59. Sipos A, Rasmussen F, Harrison G, Tynelius P, Lewis G, Leon DA, Gunnell D: **Paternal Age And Schizophrenia: A Population Based Cohort Study**. *British Medical Journal* 2004, **329**(7474):1070-1073.

60. Werner S, Malaspina D, Rabinowitz J: **Socioeconomic status at birth is associated with risk of schizophrenia: population-based multilevel study**. *Schizophr Bull* 2007, **33**(6):1373-1378.

61. Corcoran C, Perrin M, Harlap S, Deutsch L, Fennig S, Manor O, Nahon D, Kimhy D, Malaspina D, Susser E: **Effect of socioeconomic status and parents’ education at birth on risk of schizophrenia in offspring**. *Social Psychiatry and Psychiatric Epidemiology* 2009, **44**(4):265-271.

62. Castle DJ, Scott K, Wessely S, Murray RM: **Does social deprivation during gestation and early life predispose to later schizophrenia?** *Soc Psychiatry Psychiatr Epidemiol* 1993, **28**(1):1-4.

63. Hare EH, Price JS, Slater E: **Parental social class in psychiatric patients**. *Br J Psychiatry* 1972, **121**(564):515-534.

64. Harrison G, Gunnell D, Glazebrook C, Page K, Kwiecinski R: **Association between schizophrenia and social inequality at birth: case-control study**. *Br J Psychiatry* 2001, **179**:346-350.

65. Koponen H, Rantakallio P, Veijola J, Jones P, Jokelainen J, Isohanni M: **Childhood central nervous system infections and risk for schizophrenia**. *European Archives of Psychiatry and Clinical Neuroscience* 2004, **254**(1):9-13.

66. Makikyro T, Isohanni M, Moring J, Oja H, Hakko H, Jones P, Rantakallio P: **Is a child's risk of early onset schizophrenia increased in the highest social class?** *Schizophrenia Research* 1997, **23**(3):245-252.

67. Mulvany F, O'Callaghan E, Takei N, Byrne M, Fearon P, Larkin C: **Effect of social class at birth on risk and presentation of schizophrenia: case-control study**. *BMJ* 2001, **323**(7326):1398-1401.

68. Seidman LJ, Cherkerzian S, Goldstein JM, Agnew-Blais J, Tsuang MT, Buka SL: **Neuropsychological performance and family history in children at age 7 who develop adult schizophrenia or bipolar psychosis in the New England Family Studies**. *Psychol Med* 2013, **43**(01):119-131.

69. Wicks S, Hjern A, Gunnell D, Lewis G, Dalman C: **Social adversity in childhood and the risk of developing psychosis: a national cohort study**. *Am J Psychiatry* 2005, **162**(9):1652-1657.

70. Marcelis M, Navarro-Mateu F, Murray R, Selten J-P, Van Os J: **Urbanization and psychosis: a study of 1942–1978 birth cohorts in The Netherlands**. *Psychol Med* 1998, **28**(04):871-879.

71. Bresnahan M, Begg MD, Brown A, Schaefer C, Sohler N, Insel B, Vella L, Susser E: **Race and risk of schizophrenia in a US birth cohort: another example of health disparity?** *International Journal of Epidemiology* 2007, **36**(4):751-758.

72. Corcoran C, Perrin M, Harlap S, Deutsch L, Fennig S, Manor O, Nahon D, Kimhy D, Malaspina D, Susser E: **Incidence of schizophrenia among second-generation immigrants in the jerusalem perinatal cohort**. *Schizophr Bull* 2009, **35**(3):596-602.

73. Burman B, Mednick SA, Machon RA, Parnas J, Schulsinger F: **Children at high risk for schizophrenia: parent and offspring perceptions of family relationships**. *Journal of Abnormal Psychology* 1987, **96**(4):364-366.

74. Goldstein MJ: **The UCLA High-Risk Project**. *Schizophr Bull* 1987, **13**(3):505-514.

75. Schiffman J, LaBrie J, Carter J, Cannon T, Schulsinger F, Parnas J, Mednick S: **Perception of parent-child relationships in high-risk families, and adult schizophrenia outcome of offspring**. *Journal of Psychiatric Research* 2002, **36**(1):41-47.

76. Walker E, Hoppes E, Emory E, Mednick S, Schulsinger F: **Environmental factors related to schizophrenia in psychophysiologically labile high-risk males**. *Journal of Abnormal Psychology* 1981, **90**(4):313-320.

77. Riordan DV, Morris C, Hattie J, Stark C: **Interbirth spacing and offspring mental health outcomes**. *Psychol Med* 2012, **42**(12):2511-2521.

78. Dalman C, Allebeck P, Gunnell D, Harrison G, Kristensson K, Lewis G, Lofving S, Rasmussen F, Wicks S, Karlsson H: **Infections in the CNS during childhood and the risk of subsequent psychotic illness: a cohort study of more than one million Swedish subjects**. *American Journal of Psychiatry* 2008, **165**(1):59-65.

79. Rantakallio P, Jones P, Moring J, Von Wendt L: **Association between central nervous system infections during childhood and adult onset schizophrenia and other psychoses: a 28-year follow-up**. *International Journal of Epidemiology* 1997, **26**(4):837-843.

80. Leask SJ, Done DJ, Crow TJ: **Adult psychosis, common childhood infections and neurological soft signs in a national birth cohort**. *British Journal of Psychiatry* 2002, **181**:387-392.

81. Orlovska S, Pedersen MS, Benros ME, Mortensen PB, Agerbo E, Nordentoft M: **Head injury as risk factor for psychiatric disorders: a nationwide register-based follow-up study of 113,906 persons with head injury**. *American Journal of Psychiatry* 2014, **171**(4):463-469.

82. Niemi LT, Suvisaari JM, Haukka JK, Lonnqvist JK: **Childhood growth and future development of psychotic disorder among Helsinki high-risk children**. *Schizophrenia Research* 2005, **76**(1):105-112.

83. Arseneault L, Cannon M, Poulton R, Murray R, Caspi A, Moffitt TE: **Cannabis use in adolescence and risk for adult psychosis: longitudinal prospective study**. *British Medical Journal* 2002, **325**(7374):1212-1213.

84. Welham J, Scott J, Williams G, Najman J, Bor W, O'Callaghan M, McGrath J: **Emotional and behavioural antecedents of young adults who screen positive for non-affective psychosis: a 21-year birth cohort study**. *Psychol Med* 2009, **39**(4):625-634.

85. Bearden CE, Rosso IM, Hollister JM, Sanchez LE, Hadley T, Cannon TD: **A prospective cohort study of childhood behavioral deviance and language abnormalities as predictors of adult schizophrenia**. *Schizophr Bull* 2000, **26**(2):395-410.

86. Meyer SE, Carlson GA, Youngstrom E, Ronsaville DS, Martinez PE, Gold PW, Hakak R, Radke-Yarrow M: **Long-term outcomes of youth who manifested the CBCL-Pediatric Bipolar Disorder phenotype during childhood and/or adolescence**. *Journal of Affective Disorders* 2009, **113**(3):227-235.

87. Amminger GP, Pape S, Rock D, Roberts SA, Ott SL, Squires-Wheeler E, Kestenbaum C, Erlenmeyer-Kimling L: **Relationship between childhood behavioral disturbance and later schizophrenia in the New York High-Risk Project**. *American Journal of Psychiatry* 1999, **156**(4):525-530.

88. Carlson GA, Weintraub S: **Childhood behavior problems and bipolar disorder - relationship or coincidence?** *Journal of Affective Disorders* 1993, **28**(3):143-153.

89. Crow TJ, Done DJ, Sacker A: **Chidhood precursors of psychiosis as clues to its evolutionary orgins**. *European Archives of Psychiatry and Clinical Neuroscience* 1995, **245**(2):61-69.

90. Done DJ, Crow TJ, Johnstone EC, Sacker A: **Childhood antecedents of schizophrenia and affective illness: Social adjustment at ages 7 and 11**. *British Medical Journal* 1994, **309**(6956):699.

91. Ekstrom M, Sorensen H, Mednick SA: **Premorbid personality in schizophrenia spectrum: A prospective study**. *Nordic Journal of Psychiatry* 2006, **60**(5):417-422.

92. Fisher HL, Caspi A, Poulton R, Meier MH, Houts R, Harrington H, Arseneault L, Moffitt TE: **Specificity of childhood psychotic symptoms for predicting schizophrenia by 38 years of age: a birth cohort study**. *Psychol Med* 2013, **43**(10):2077-2086.

93. Poulton R, Caspi A, Moffitt TE, Cannon M, Murray R, Harrington H: **Children's self-reported psychotic symptoms and adult schizophreniform disorder: A 15-year longitudinal study**. *Archives of General Psychiatry* 2000, **57**(11):1053-1058.

94. Niemi LT, Suvisaari JM, Haukka JK, Lonnqvist JK: **Childhood predictors of future psychiatric morbidity in offspring of mothers with psychotic disorder: results from the Helsinki High-Risk Study**. *British Journal of Psychiatry* 2005, **186**:108-114.

95. Ott SL, Roberts S, Rock D, Allen J, Erlenmeyer-Kimling L: **Positive and negative thought disorder and psychopathology in childhood among subjects with adulthood schizophrenia**. *Schizophrenia Research* 2002, **58**(2-3):231-239.

96. Schiffman J, Walker E, Ekstrom M, Schulsinger F, Sorensen H, Mednick S: **Childhood videotaped social and neuromotor precursors of schizophrenia: a prospective investigation**. *American Journal of Psychiatry* 2004, **161**(11):2021-2027.

97. Cornblatt BA, Obuchowski M, Roberts S, Pollack S, Erlenmeyer–Kimling L: **Cognitive and behavioral precursors of schizophrenia**. *Development and Psychopathology* 1999, **11**(03):487-508.

98. Kim-Cohen J, Caspi A, Moffitt TE, Harrington H, Milne BJ, Poulton R: **Prior juvenile diagnoses in adults with mental disorder: Developmental follow-back of a prospective-longitudinal cohort**. *Archives of General Psychiatry* 2003, **60**(7):709-717.

99. Olin SS, John RS, Mednick SA: **Assessing the predictive value of teacher reports in a high risk sample for schizophrenia: a ROC analysis**. *Schizophrenia Research* 1998, **16**(1):53-66.

100. Parnas J, Jorgensen A: **Pre-morbid psychopathology in schizophrenia spectrum**. *British Journal of Psychiatry* 1989, **155**:623-627.

101. Reichart CG, van der Ende J, Wals M, Hillegers MHJ, Nolen WA, Ormel J, Verhulst FC: **The use of the GBI as predictor of bipolar disorder in a population of adolescent offspring of parents with a bipolar disorder**. *Journal of Affective Disorders* 2005, **89**(1-3):147-155.

102. Stringaris A, Cohen P, Pine DS, Leibenluft E: **Adult Outcomes of Youth Irritability: A 20-Year Prospective Community-Based Study**. *American Journal of Psychiatry* 2009, **166**(9):1048-1054.

103. Ullman VZ, Levine SZ, Reichenberg A, Rabinowitz J: **Real-world premorbid functioning in schizophrenia and affective disorders during the early teenage years: A population-based study of school grades and teacher ratings**. *Schizophrenia Research* 2012, **136**(1–3):13-18.

104. Cannon TD, Bearden CE, Hollister JM, Rosso IM, Sanchez LE, Hadley T: **Childhood Cognitive Functioning in Schizophrenia Patients and Their Unaffected Siblings: A Prospective Cohort Study**. *Schizophr Bull* 2000, **26**(2):379-393.

105. Chong S, Subramaniam M, Lee IM, Pek E, Cheok C, Verma S, Wong J: **Academic attainment: a predictor of psychiatric disorders?** *Social Psychiatry and Psychiatric Epidemiology* 2009, **44**(11):999-1004.

106. Jones P, Murray R, Rodgers B, Marmot M: **Child developmental risk factors for adult schizophrenia in the British 1946 birth cohort**. *The Lancet* 1994, **344**(8934):1398-1402.

107. Koenen KC, Moffitt TE, Roberts AL, Martin LT, Kubzansky L, Harrington H, Poulton R, Caspi A: **Childhood IQ and adult mental disorders: a test of the cognitive reserve hypothesis**. *American Journal of Psychiatry* 2009, **166**(1):50-57.

108. Kremen WS, Vinogradov S, Poole JH, Schaefer CA, Deicken RF, Factor-Litvak P, Brown AS: **Cognitive decline in schizophrenia from childhood to midlife: a 33-year longitudinal birth cohort study**. *Schizophrenia Research* 2010, **118**(1-3):1-5.

109. Meyer SE, Carlson GA, Wiggs EA, Martinez PE, Ronsaville DS, Klimes-dougan B, Gold PW, Radke-yarrow M: **A prospective study of the association among impaired executive functioning, childhood attentional problems, and the development of bipolar disorder**. *Development and Psychopathology* 2004, **16**(2):461-476.

110. Niendam TA, Bearden CE, Rosso IM, Sanchez LE, Hadley T, Nuechterlein KH, Cannon TD: **A prospective study of childhood neurocognitive functioning in schizophrenic patients and their siblings**. *American Journal of Psychiatry* 2003, **160**(11):2060-2062.

111. Osler M, Lawlor DA, Nordentoft M: **Cognitive function in childhood and early adulthood and hospital admission for schizophrenia and bipolar disorders in Danish men born in 1953**. *Schizophrenia Research* 2007, **92**(1–3):132-141.

112. Ott SL, Spinelli S, Rock D, Roberts S, Amminger GP, Erlenmeyer-Kimling L: **The New York High-Risk Project: social and general intelligence in children at risk for schizophrenia**. *Schizophrenia Research* 1998, **31**(1):1-11.

113. Schulz J, Sundin J, Leask S, Done DJ: **Risk of adult schizophrenia and its relationship to childhood IQ in the 1958 British birth cohort**. *Schizophr Bull* 2014, **40**(1):143-151.

114. Sørensen HJ, Mortensen EL, Schiffman J, Ekstrøm M, Denenney D, Mednick SA: **Premorbid IQ and adult schizophrenia spectrum disorder: Verbal Performance subtests**. *Psychiatry Research* 2010, **178**(1):23-26.

115. Isohanni I, Jarvelin M-R, Nieminen P, Jones P, Rantakallio P, Jokelainen J, Isohanni M: **School performance as a predictor of psychiatric hospitalization in adult life. A 28-year follow-up in the Northern Finland 1966 Birth Cohort**. *Psychol Med* 1998, **28**(04):967-974.

116. MacCabe JH, Wicks S, Lofving S, David AS, Berndtsson A, Gustafsson JE, Allebeck P, Dalman C: **Decline in cognitive performance between ages 13 and 18 years and the risk for psychosis in adulthood: a Swedish longitudinal cohort study in males**. *JAMA Psychiatry* 2013, **70**(3):261-270.

117. Sorensen HJ, Mortensen EL, Parnas J, Mednick SA: **Premorbid neurocognitive functioning in schizophrenia spectrum disorder**. *Schizophr Bull* 2006, **32**(3):578-583.

118. Mouridsen SE, Hauschild K-M: **A longitudinal study of schizophrenia- and affective spectrum disorders in individuals diagnosed with a developmental language disorder as children**. *Journal of Neural Transmission* 2008, **115**(11):1591-1597.

119. Griffith JJ, Mednick SA, Schulsinger F, Diderichsen B: **Verbal associative disturbances in children at high risk for schizophrenia**. *Journal of Abnormal Psychology* 1980, **89**(2):125-131.

120. Isohanni M, Jones PB, Moilanen K, Rantakallio P, Veijola J, Oja H, Koiranen M, Jokelainen J, Croudace T, Järvelin MR: **Early developmental milestones in adult schizophrenia and other psychoses. A 31-year follow-up of the Northern Finland 1966 Birth Cohort**. *Schizophrenia Research* 2001, **52**(1–2):1-19.

121. Rosso IM, Bearden CE, Hollister JM, Gasperoni TL, Sanchez LE, Hadley T, Cannon TD: **Childhood neuromotor dysfunction in schizophrenia patients and their unaffected siblings: a prospective cohort study**. *Schizophr Bull* 2000, **26**(2):367-378.

122. Cannon M, Jones P, Murray RM, Wadsworth ME: **Childhood laterality and later risk of schizophrenia in the 1946 British birth cohort**. *Schizophrenia Research* 1997, **26**(2-3):117-120.

123. Erlenmeyer-Kimling L, Rock D, Roberts SA, Janal M, Kestenbaum C, Cornblatt B, Adamo UH, Gottesman II: **Attention, memory, and motor skills as childhood predictors of schizophrenia-related psychoses: the New York High-Risk Project**. *American Journal of Psychiatry* 2000, **157**(9):1416-1422.

124. Schiffman J, Ekstrom M, LaBrie J, Schulsinger F, Sorensen H, Mednick S: **Minor physical anomalies and schizophrenia spectrum disorders: a prospective investigation**. *American Journal of Psychiatry* 2002, **159**(2):238-243.

125. Schiffman J, Pestle S, Mednick S, Ekstrom M, Sorensen H, Mednick S: **Childhood laterality and adult schizophrenia spectrum disorders: a prospective investigation**. *Schizophrenia Research* 2005, **72**(2-3):151-160.

126. Schiffman J, Maeda JA, Hayashi K, Michelsen N, Sorensen HJ, Ekstrom M, Abe KA, Chronicle EP, Mednick SA: **Premorbid childhood ocular alignment abnormalities and adult schizophrenia-spectrum disorder**. *Schizophrenia Research* 2006, **81**(2-3):253-260.

127. Schiffman J, Sorensen HJ, Maeda J, Mortensen EL, Victoroff J, Hayashi K, Michelsen NM, Ekstrom M, Mednick S: **Childhood motor coordination and adult schizophrenia spectrum disorders**. *American Journal of Psychiatry* 2009, **166**(9):1041-1047.
