# Supplementary material for: Common or distinct pathways to psychosis? A systematic review of evidence from prospective studies for developmental risk factors and antecedents of the schizophrenia spectrum disorders and affective psychoses
Source: BMC Psychiatry. 2015 Aug 25;15:205. doi: 10.1186/s12888-015-0562-2 (PMC4548447; doi:10.1186/s12888-015-0562-2)
Supplement: Additional file 2: Table S1. — Conception, pregnancy, and birth risk factors. Table S2. Demographic or familial risk factors. Table S3. Childhood and adolescent risk factors. Table S4. Childhood and adolescent antecedents. (DOCX 545 kb) [file 12888_2015_562_MOESM2_ESM.docx]

| **Supplementary Table 5:** Study details (cohort/sample; study design; outcome measure; exposure measure; variables of adjustment) | | | | |
| --- | --- | --- | --- | --- |
| **Study citation** | **Cohort / Sample**  **(Study design)** | **Psychiatric outcome measure** | **Antecedent/risk factor measure** | **Analyses adjusted for the following variables:** |
| [[1](#_ENREF_1)] Amminger et al (1999) | *New York High-Risk Project* (High-risk) | SADS-L and RDC | Parental report on behavioural items (composite score) | Substance abuse, gender |
| [[2](#_ENREF_2)] Arseneault et al (2002) | *Dunedin Multidisciplinary Health and Development Study* (Birth cohort) | DIS (DSM-IV) | Self-report | SES, sex, other drug use, strong psychotic symptoms at age 11 (after controlling for this, cannabis users still had significantly more psychotic symptoms at age 26, but schizophreniform outcome no longer significant) |
| [[3](#_ENREF_3)] Babulus et al (2006) | *Prenatal Determinants of Schizophrenia Study* (Birth cohort) | DIGS or chart review | Child Health and Development Study interview data | Maternal age, maternal race, maternal education, and maternal mental illness |
| [[4](#_ENREF_4)] Bain et al (2000)  [[56](#_ENREF_56)] Kendell et al (2000) | *1971-74 Scottish birth cohort* (Nested case-control) | Scottish Morbidity Record 4 (ICD-9) | Scottish Morbidity Record 2 (Cumulative register of all hospital births in Scotland) | Matched controls on DOB, obstetric unit of birth, gender, maternal age, maternal parity, paternal occupation |
| [[5](#_ENREF_5)] Bao et al (2012) | *Prenatal Determinants of Schizophrenia Study* (Nested case-control) | KPMCP, chart review and/or diagnostic interview (ICD-9) | Archived maternal serum samples obtained during pregnancy assayed for vitamin A | Maternal age, maternal education  Controls matched on membership to KPMCP at time of case ascertainment, DOB, sex, gestational timing of first maternal serum sample, number of maternal blood samples drawn during pregnancy |
| [[6](#_ENREF_6)] Bearden et al (2000) | *National Collaborative Perinatal Project* (Birth cohort) | Chart-review (DSM-IV) | *Deviant behaviour* – assigned by clinician (list of items)  *Social maladjustment* – behavioural assessments conducted by clinical psychologist | Race, gender, parental education level, parental socioeconomic status, and age at time of examination |
| [[7](#_ENREF_7)] Bresnahan et al (2007) | *Prenatal Determinants of Schizophrenia Study* (Birth cohort) | DIGS or chart review (DSM-IV) | Child Health and Development Study maternal intake interview | Maternal education, paternal occupation, family income, year of intake, marital status |

| **Study citation** | **Cohort / Sample**  **(Study design)** | **Psychiatric outcome measure** | **Antecedent/risk factor measure** | **Analyses adjusted for the following variables:** |
| --- | --- | --- | --- | --- |
| [[8](#_ENREF_8)] Brown et al (2000) | *Prenatal Determinants of Schizophrenia Study* (Birth cohort) | DIGS or chart review | Health Plan charts (prior to birth) | Maternal age, smoking, education, race, parity, alcohol use, and marital status (maternal smoking, race and education included in final model) |
| [[9](#_ENREF_9)] Brown et al (2004) | *Prenatal Determinants of Schizophrenia Study* (Nested case-control) | DIGS or chart review (DSM-IV) | Child Health and Development Study maternal intake interview; Maternal serum samples obtained during pregnancy | Maternal age, maternal ethnicity, SES (maternal education), maternal smoking, gestational age of serum sample  Controls matched on DOB, gender, number and timing of maternal blood samples taken during index pregnancy |
| [[10](#_ENREF_10)] Brown et al (2004) | *Prenatal Determinants of Schizophrenia Study* (Nested case-control) | DIGS (DSM-IV) | Maternal serum samples collected during pregnancy – KFHP | Matched controls on membership in KFHP at time of case ascertainment, DOB, sex, number of maternal blood samples drawn, number of weeks after last menstrual period of first maternal blood draw during pregnancy |
| [[11](#_ENREF_11)] Brown et al (2005) | *Prenatal Determinants of Schizophrenia Study* (Nested case-control) | DIGS or chart review (DSM-IV) | Maternal serum samples obtained during pregnancy | Maternal age (older mothers had higher levels), maternal ethnicity, SES (maternal education), gestational age of serum sample  Controls matched on DOB, gender, number and timing of first maternal blood sample taken during index pregnancy, gestational age of serum sample |
| [[12](#_ENREF_12)] Brown et al (2006) | *Prenatal Determinants of Schizophrenia Study* (Nested case-control) | DIGS or chart review (DSM-IV) | Maternal serum samples (from pregnancy) | Maternal education and race  Matched controls on DOB, gender, time in cohort, availability of maternal sera |
| [[13](#_ENREF_13)] Brown et al (2006) | *Prenatal Determinants of Schizophrenia Study* (Birth cohort) | DIGS or chart review (DSM-IV) | Child Health and Development Study Interview | Maternal age, paternal education, paternal race, and parity |
| [[14](#_ENREF_14)] Buizer-Voskamp et al (2011) | *Netherlands population-based study* (Population cohort) | Psychiatric Case Registry Middle Netherlands | Civil Registry of Statistics Netherlands | Income neighbourhood, difference in age between father and mother, ethnic background |
| [[15](#_ENREF_15)] Buka et al (2008) | *National Collaborative Perinatal Project* (Nested case-control) | SCID (DSM-IV) | Maternal serum samples | Maternal education, parental treatment for mental disorder  Matched controls on study site, gender, race, DOB, parental history of treatment for mental disorder |

| **Study citation** | **Cohort / Sample**  **(Study design)** | **Psychiatric outcome measure** | **Antecedent/risk factor measure** | **Analyses adjusted for the following variables:** |
| --- | --- | --- | --- | --- |
| [[16](#_ENREF_16)] Burman et al (1987) | *Danish High-Risk Project* (High-Risk) | CAPPS, PSE, clinical interview | Perception of Relationship with Mother/Father Scale |  |
| [[17](#_ENREF_17)] Canetta et al (in press) | *Finnish Prenatal Study of Schizophrenia* (Nested case-control) | Finnish Hospital Discharge and Outpatient Registry | Sera drawn during first and early second trimesters of pregnancy | Maternal age, maternal education, number of previous births, maternal and parental history of schizophrenia, other non-affective psychotic disorders, and affective or other psychiatric disorders, gestational week of the maternal blood draw, twin/singleton birth, urban/semiurban/rural birth, province at birth  Matched controls on date of birth, sex, and residence in Finland at time of diagnosis |
| [[18](#_ENREF_18)] Canetta et al (2014) | *Child Health and Development Study* (Nested case-control) | SCID/DIGS | Sera drawn during pregnancy | Maternal age, race, education, and psychiatric history  Matched controls on date of birth, sex, gestational timing or availability of maternal archived sera |
| [[19](#_ENREF_19)] Cannon et al (1997) | *1946 British Birth Cohort* (Birth cohort) | Mental Health Inquiry (DSM-III-R) | Examination by school doctors |  |
| [[20](#_ENREF_20)] Cannon et al (2002) | *Dunedin Multidisciplinary Health and Development Study* (Birth cohort) | DIS (DSM-IV) | Birth and prenatal information from hospital records | Sex, SES |
| [[21](#_ENREF_21)] Cannon et al (2000) | *National Collaborative Perinatal Project* (Birth cohort) | Chart review (DSM-IV) | Stanford-Binet |  |
| [[22](#_ENREF_22)] Cantor-Graae et al (1997) | *Swedish case-control study* (Case-control) | Psychiatric inpatients; RDC | Pregnancy and birth records | Matched controls on same obstetric clinic, gender, maternal parity, maternal age, paternal occupation, parental marital status at delivery |
| [[23](#_ENREF_23)] Carlson et al (1993) | *Stony Book High-Risk Project* (High-Risk) | SADS-L (DSM-III) | Parent, teacher and peer rated Behavior and Attention Problems Scales |  |
| [[24](#_ENREF_24)] Carter et al (2003) | *Copenhagen High-Risk Project* (High-Risk) | PSE; CAPPS | Birth records |  |

| **Study citation** | **Cohort / Sample**  **(Study design)** | **Psychiatric outcome measure** | **Antecedent/risk factor measure** | **Analyses adjusted for the following variables:** |
| --- | --- | --- | --- | --- |
| [[25](#_ENREF_25)] Castle et al (1993) | *British case-control study* (Case-control) | Camberwell Cumulative Psychiatric Case Register | Medical or birth records | Low SES area of residence  Matched controls on age and gender |
| [[26](#_ENREF_26)] Chong et al (2009) | *Singapore population-based study* (Population cohort) | CIDI-AUTO (DSM-IV) | Primary School Leaving Examination score | Ethnicity |
| [[27](#_ENREF_27)] Clarke et al (2009) | *Finnish population-based study* (Population cohort) | FHDR | Finnish medical birth register, Finnish Population Register | Maternal psychotic illness |
| [[28](#_ENREF_28)] Clarke et al (2011) | *Helsinki birth cohort* (Nested case-control) | FHDR | Child Health Archives | Matched controls on gender and DOB |
| [[30](#_ENREF_30)] Cocoran et al (2009) | *Jerusalem Perinatal Study* (Birth cohort) | National Psychiatric Registry | *Immigration* : Paternal occupation | Sex, maternal age, duration of marriage, birth order |
| [[29](#_ENREF_29)] Cocoran et al (2009) | *Jerusalem Perinatal Study* (Birth cohort) | National Psychiatric Registry | *SES:* Parental birth certificates | Paternal age and maternal age, maternal education, paternal social class, sex, birth order |
| [[31](#_ENREF_31)] Cornblatt et al (1999) | *New York High-Risk Project* (High-Risk) | RDC, SADS-L | Behavioural Global Adjustment Scale (completed by parents) |  |
| [[32](#_ENREF_32)] Crow et al (1995)  [[34](#_ENREF_34)] Done et al (1994) | *National Child Development Study* (Birth cohort) | Hospital admission records | Bristol Social Adjustment Guide (teacher report) |  |
| [[33](#_ENREF_33)] Dalman et al (2008) | *Swedish birth cohort* (Birth cohort) | Swedish National Inpatient Register | Swedish National Inpatient Register | Sex, Age, Urbanicity, Parental Psychosis |
| [[35](#_ENREF_35)] Ekstrom et al (2006) | *Copenhagen Perinatal Cohort* (Birth cohort) | SCID (DSM-III-R) or hospital records | Adjective Check List (ACL) | Controls matched on gender, mother’s marital status at time of conception, pregnancy number, social class, mother’s height and weight, maternal and paternal age |
| [[36](#_ENREF_36)] Erlenmeyer-Kimling et al (2000) | *New York High-Risk Project* (High-Risk) | SADS-L | Lincoln-Oseretsky Motor Development Scale | Gender, recruitment sample, age at testing |

| **Study citation** | **Cohort / Sample**  **(Study design)** | **Psychiatric outcome measure** | **Antecedent/risk factor measure** | **Analyses adjusted for the following variables:** |
| --- | --- | --- | --- | --- |
| [[37](#_ENREF_37)] Fisher et al (2013) *(update of [*[*96*](#_ENREF_96)*] Poulton et al 2000)* | *Dunedin Multidisciplinary Health and Development Study* (Birth cohort) | DIS (DSM-IV) | Diagnostic Interview Schedule for Children (DIS-C) | SES, diagnosis in childhood of attention-deficit hyperactivity disorder or conduct disorder |
| [[38](#_ENREF_38)] Freedman et al (2013) | *Child Health and Development Study* (Nested case-control) | DIGS | Recorded at birth (medical records) | Controls matched on: Kaiser Permanente membership at time of case ascertainment, date of birth, availability of maternal serum. |
| [[39](#_ENREF_39)] Goldstein (1987) | *UCLA High-Risk Project* (High-Risk) | Structured interview (DSM-III) | Thematic Apperception Test; Measures of Expressed Emotion and Affective Style |  |
| [[40](#_ENREF_40)] Griffith et al (1980) | *Danish High-Risk Study* (High-Risk) | CAPPS, PSE, clinical interview | Single-Word Association Test; Continuous Association Test |  |
| [[41](#_ENREF_41)] Gunther-Genta et al (1994) | *Swiss case-control study* (Case-control study) | Psychiatric clinic | Obstetric files | Controls matched on sex, month of birth, and birth order |
| [[42](#_ENREF_42)] Hare et al (1972) | *British case-control study* (Case-control) | Hospital inpatient (ICD-8) | Birth certificate |  |
| [[43](#_ENREF_43)] Harper et al (2011) | *Prenatal Determinants of Schizophrenia Study* (Nested case-control) | DIGS or chart review (DSM-IV) | Maternal serum samples obtained during pregnancy | Maternal education, gestational age at blood draw, maternal race, maternal psychosis  Controls matched on DOB, sex, number of maternal blood samples drawn, number of weeks after last menstrual period of first maternal blood draw, gestational age at serum collection |
| [[44](#_ENREF_44)] Harrison et al (2001) | *British case-control study* (Case-control) | SCAN (ICD-10) | Birth certificate | Controls matched on DOB, gender, and area of birth registration |

| **Study citation** | **Cohort / Sample**  **(Study design)** | **Psychiatric outcome measure** | **Antecedent/risk factor measure** | **Analyses adjusted for the following variables:** |
| --- | --- | --- | --- | --- |
| [[45](#_ENREF_45)] Haukka et al (2004) | *Finnish case-sibling study* (Case-control) | FHDR | Population Register Centre | Sex, ages of other siblings, birth order, birth cohort, maternal age, paternal age  Family size additionally adjusted for sex of youngest proband, lowest age-at-onset in the sibship, and whether or the mother was schizophrenic |
| [[46](#_ENREF_46)] Herman et al (2006) | *Prenatal Determinants of Schizophrenia Study* (Birth cohort) | DIGS or chart review (DSM-IV) | Interview at first contact with prenatal clinic | Parental age, paternal occupation, maternal race, maternal education, maternal marital status, family income, birth weight |
| [[47](#_ENREF_47)] Hollister et al (1996) | *Danish perinatal project* (Birth cohort) | National Psychiatric Register | Data concerning pregnancy collected by obstetricians | Parental diagnosis, age, marital status, number of previous pregnancies, length of gestation, and offspring mortality, gender (significant for males) |
| [[48](#_ENREF_48)] Hultman et al (1997) | *Swedish case-control study* (Case-control) | Adult psychiatric inpatient facility | Birth records examined by midwife | Controls matched on gender, DOB, place of birth  Gender, age at delivery, toxaemic signs, early rupture of membranes, instrumental delivery, head circumference, birth weight for body length |
| [[49](#_ENREF_49)] Hultman et al (1999) | *Swedish population-based study* (Case-control) | Swedish inpatient register | Swedish inpatient register | Maternal age at delivery, parity, hypertensive disease, diabetes, bleeding during pregnancy, uterine atony, birth weight for gestational age, ponderal index, apgar score at 1 minute, asphyxia, late winter birth  Matched controls on sex, year of birth, and hospital of birth |
| [[50](#_ENREF_50)] Isohanni et al (1998) | *Northern Finland 1966 Birth Cohort* (Birth cohort) | FHDR; OPCRIT (DSM-III-R) | School class level | School marks, sex, parental social class, place of residence, family type |
| [[51](#_ENREF_51)] Isohanni et al (2001) | *Northern Finland 1966 Birth Cohort* (Birth cohort) | FHDR case records validated for DSM-III-R criteria | Information obtained from visits to welfare centres and an examination by public health nurses and general practitioners |  |
| [[52](#_ENREF_52)] Jones et al (1994) | *1946 British Birth Cohort* (Birth cohort) | Central registers of admissions to psychiatric hospitals | Group tests on non-verbal, verbal, reading abilities, arithmetic and vocabulary | Sex, SES |

| **Study citation** | **Cohort / Sample**  **(Study design)** | **Psychiatric outcome measure** | **Antecedent/risk factor measure** | **Analyses adjusted for the following variables:** |
| --- | --- | --- | --- | --- |
| [[53](#_ENREF_53)] Jones et al (1998) | *Northern Finland 1966 Birth Cohort* (Birth cohort) | OPCRIT (DSM-III-R) | Information obtained from prenatal clinics, hospital delivery records and postnatal clinic visits | Sex, SES, status at birth, maternal depression |
| [[54](#_ENREF_54)] Kawai et al (2004) | *Japanese case-control study* (Case-control) | Patients at psychiatric hospitals | Mother and Child Health Handbooks | Number of antenatal care visits and BMI adjusted for age of participants, birth order of participants, gestational age at either first or last antenatal care visit  Obstetric complications adjusted for age of subjects, birth order of subjects, number of antenatal care visits, BMI at the first antenatal care visit |
| [[55](#_ENREF_55)] Kemppainen et al (2000) | *Northern Finland 1966 Birth Cohort* (Birth cohort) | FHDR | Information collected from pregnancy |  |
| [[57](#_ENREF_57)] Khashan et al (2008) | *Danish population study* (Population cohort) | Danish Psychiatric Central Register | Civil Registration System; National Hospital Register | Offspring age, sex, place of birth, family history, maternal age, calendar year, unknown father, statistical interaction between offspring age and sex |
| [[58](#_ENREF_58)] Kim-Cohen et al (2003) | *Dunedin Multidisciplinary Health and Development Study* (Birth cohort) | DIS (DSM-IV) | DIS-C | Sex |
| [[59](#_ENREF_59)] Koenen et al (2009) | *Dunedin Multidisciplinary Health and Development Study* (Birth cohort) | DIS (DSM-IV) | WISC-R |  |
| [[60](#_ENREF_60)] Koponen et al (2004) | *Northern Finland 1966 Birth Cohort* (Birth cohort) | FHDR | FHDR | Father’s social class, perinatal brain damage, mental retardation, childhood epilepsy |
| [[61](#_ENREF_61)] Kremen et al (2010) | *Prenatal Determinants of Schizophrenia Study* (Nested case-control) | DIGS or chart review | Peabody Picture Vocabulary Test |  |

| **Study citation** | **Cohort / Sample**  **(Study design)** | **Psychiatric outcome measure** | **Antecedent/risk factor measure** | **Analyses adjusted for the following variables:** |
| --- | --- | --- | --- | --- |
| [[62](#_ENREF_62)] Laursen et al (2007) | *Danish population study* (Population cohort) | Danish Psychiatric Central Register | Danish Medical Birth Register | *For obstetric variables:* Family history of psychiatric admission, place of birth, maternal age, paternal age, loss of a parent  *For paternal age:*  Age, calendar time, gender, family history of psychiatric admission, maternal age, loss of parent, place of birth  *For urban birth:*  Age, calendar time, gender, family history of psychiatric admission, maternal age, loss of parent, and paternal age |
| [[63](#_ENREF_63)] Leask et al (2002) | *National Child Development Study* (Birth cohort) | PSE diagnoses derived from psychiatric hospital case notes | Medical examinations by school medical officer and home interview with child’s main carer | Gender, social class |
| [[64](#_ENREF_64)] MacCabe et al (2013) | *Swedish population study* (Population cohort) | National Patient Register | Standardised timed tests assessing verbal, spatial, and inductive ability |  |
| [[65](#_ENREF_65)] Machon et al (1987) | *Danish High-Risk Study* (High-Risk) | CAPPS, PSE |  |  |
| [[66](#_ENREF_66)] Maki et al (2010) | *Northern Finland 1966 Birth Cohort* (Birth cohort) | SCID-I | Interview at antenatal clinic | Sex, obstetric complications |
| [[67](#_ENREF_67)] Makikyro et al (1997) | *Northern Finland 1966 Birth Cohort* (Birth cohort) | Case records OPCRIT (DSM-III-R) | Father’s occupation and prestige |  |
| [[68](#_ENREF_68)] Marcelis et al (1998) | *Dutch population-based study* (Population cohort) | SIG (ICD-9) | Defined by 1993 municipal population density |  |
| [[69](#_ENREF_69)] Mathiasen et al (2011) | *Danish population study* (Population cohort) | Central Psychiatric Research Register | Fertility register | Calendar time, age, gestational age, parental education, sex, plurality, SGA, cerebral palsy, paternal age, parental psychiatric status at birth or within one year of birth |
| [[70](#_ENREF_70)] McGrath et al (2004) | *Northern Finland 1966 Birth Cohort* (Birth cohort) | FHDR | Information collected during pregnancy | Parity, gestational and maternal age, length of maternal education, SES, birth weight |
| [[71](#_ENREF_71)] McGrath et al (2010) | *Danish case-control study* (Case-control) | Danish Psychiatric Central Register (ICD-10) | Neonatal bio-bank | Controls matched on gender, DOB, and birth in Denmark |

| **Study citation** | **Cohort / Sample**  **(Study design)** | **Psychiatric outcome measure** | **Antecedent/risk factor measure** | **Analyses adjusted for the following variables:** |
| --- | --- | --- | --- | --- |
| [[72](#_ENREF_72)] Menezes et al (2010) | *Swedish 1973-1980 Birth Cohort* (Birth cohort) | Swedish inpatient discharge and cause of death and emigration registers (ICD9-10) | Population and housing census | Sex, age, gestational age, family history of psychosis, SES, parental education, maternal age |
| [[73](#_ENREF_73)] Meyer et al (2004) | *US High-Risk Study* (High-Risk) | SCID-I (DSM-IV) | WISC-R |  |
| [[74](#_ENREF_74)] Meyer et al (2009) | *US High-Risk Study* (High-Risk) | SCID-I | CBCL (mother rated) |  |
| [[75](#_ENREF_75)] Moilanen et al (2010) | *Northern Finland 1966 Birth Cohort* (Birth cohort) | FHDR diagnoses validated using DSM-III-R criteria /SCID | Measured and recorded immediately after birth | Sex, parental history of psychosis |
| [[76](#_ENREF_76)] Monfils et al (2009) | *Swedish population study* (Population cohort) | Hospital discharge register | Swedish Medical Birth Registers | Medical diagnoses related to pregnancy/delivery, SES variables (maternal age, parity, parental education, marital status of mother at time of birth, parental country of origin) |
| [[77](#_ENREF_77)] Mortensen et al (2010) | *Danish National Birth Cohort* (Nested case-control) | Danish psychiatric case register | Blood samples from the Newborn Screening Biobank | Matched controls on gender, DOB  Maternal, paternal and sibling history of mental illness, urbanisation of place of birth, paternal and maternal age at birth of child, gestational age, immigration status |
| [[78](#_ENREF_78)] Mouridsen et al (2008) | *Danish population study* (Population cohort) | Danish Psychiatric Case Register | Referred to speech and hearing institute by speech and language therapist |  |
| [[79](#_ENREF_79)] Mulvany et al (2001) | *Irish case-control study* (Case-control) | Psychiatric Hospital Inpatients (ICD-9) | General Register Office | Controls matched on gender and birth registration district |
| [[80](#_ENREF_80)] Nielsen et al (2013) | *Danish population study* (Population cohort) | Danish psychiatric case register | Danish hospital register | Calendar year, age, sex, interaction between age and sex, family history of psychosis |
| [[81](#_ENREF_81)] Niemi et al (2004) | *Helsinki High-Risk Study* (High-Risk) | FHDR | Major Symptoms of Schizophrenia Scale; Global rating of bizarre behaviour from Scale for the Assessment of Positive Symptoms |  |

| **Study citation** | **Cohort / Sample**  **(Study design)** | **Psychiatric outcome measure** | **Antecedent/risk factor measure** | **Analyses adjusted for the following variables:** |
| --- | --- | --- | --- | --- |
| [[82](#_ENREF_82)] Niemi et al (2005) | *Helsinki High-Risk study* (High-Risk) | FHDR and out-patient treatment records | Childhood health cards | Gender, SES |
| [[83](#_ENREF_83)] Niemi et al (2005) | *Helsinki High-Risk study* (High-Risk) | FHDR | Childhood health cards |  |
| [[84](#_ENREF_84)] Niendam et al (2003) | *National Collaborative Perinatal Project* (Birth cohort) | Chart review (DSM-IV) | WISC |  |
| [[85](#_ENREF_85)] Nosarti et al (2012) | *Swedish population cohort* (Population cohort) | Swedish National Hospital Discharge Register | Swedish Medical Birth Register | Sex, parity, maternal age at delivery, maternal education, maternal psychiatric family history, gestational age, birth weight for gestational age, Apgar score at 5 min |
| [[86](#_ENREF_86)] Olin et al (1998) | *Copenhagen High-Risk Study* (High-Risk) | SADS-L, PSE, PDE | Teacher report (questionnaire) |  |
| [[87](#_ENREF_87)] Orlovska et al (2014) | *Danish population study* (Population cohort) | Danish Psychiatric Central Register | Danish National Hospital Register |  |
| [[88](#_ENREF_88)] Osler et al (2007) | *Project Metropolit* (Population cohort) | Danish Psychiatric Central Register | Härnquist test | Socioeconomic position (father’s occupational social class at birth, born outside of marriage), birth weight, educational attainment, indicators of social integration (marital status and labour participation), cognitive function at other ages |
| [[89](#_ENREF_89)] Ott et al (1998) | *New York High-Risk Project* (High-Risk) | RDC, SADS-L | WISC/WISC-R or WAIS/WAIS-R |  |
| [[90](#_ENREF_90)] Ott et al (2002) | *New York High-Risk Project* (High-Risk) | SADS-L and RDC | Scale for the Assessment of Thought, Language and Communication | Intelligence (IQ); SES; parental risk |
| [[91](#_ENREF_91)] Parboosing et al (2013) | *Child Health and Development Study* (Nested case-control) | SCID-I/P (DSM-IV-TR) | Maternal medical records | Maternal age, race, educational level, psychiatric history and gestational age at birth Matched controls on DOB |
| [[92](#_ENREF_92)] Parnas et al (1982) | *Danish High-Risk Study* (High-Risk) | CAPPS; PSE | Birth records |  |
| [[93](#_ENREF_93)] Parnas et al (1989) | *Danish High-Risk Study* (High-Risk) | PSE; CAPPS | Premorbid behaviour scales (derived from the ACL and SBQ) |  |

| **Study citation** | **Cohort / Sample**  **(Study design)** | **Psychiatric outcome measure** | **Antecedent/risk factor measure** | **Analyses adjusted for the following variables:** |
| --- | --- | --- | --- | --- |
| [[94](#_ENREF_94)] Perrin et al (2010) | *Jerusalem Perinatal Study* (Birth cohort) | National psychiatric registry | Population Registry, National psychiatric registry | Maternal age, maternal occupation, duration of marriage, paternal SES, paternal education, birth order and history of schizophrenia hospitalisation in other first degree family members |
| [[95](#_ENREF_95)] Perrin et al (2007) | *Jerusalem Perinatal Study* (Birth cohort) | Diagnostic Interview for Genetic Studies (DIGS; Nurnberger et al 1994) or chart review | Records from regular paediatric visits (from birth to age 13 years) |  |
| [[96](#_ENREF_96)] Poulton et al (2000) | *Dunedin Multidisciplinary Health and Development Study* (Birth cohort) | DIS (DSM-IV) | DIS-C (schizophrenia section) |  |
| [[97](#_ENREF_97)] Preti et al (2000) | *Italian case-control study* (Case-control) | Inpatient at Adult Psychiatric Hospital Unit (ICD-9) | Obstetric records | Controls matched on: gender, date of birth, parity of pregnancy, maternal age, marital status |
| [[98](#_ENREF_98)] Rantakallio et al (1997) | *Northern Finland 1966 Birth Cohort* (Birth cohort) | FHDR | Admissions to children’s hospitals; FHDR; neurological outpatient clinics | Father’s social class, perinatal brain damage, mental retardation, childhood epilepsy, and hearing defects |
| [[99](#_ENREF_99)] Reichart et al (2005) | *Dutch High-Risk Study* (High-Risk) | SCID (DSM-IV) | General Behaviour Inventory (GBI) |  |
| [[100](#_ENREF_100)] Riordan et al (2012) | *Scottish population-based study* (Population cohort) | SMR (ICD9/10) | Scottish Morbidity Record (SMR) | Birth weight, maternal age, maternal parity, family size and SES deprivation |
| [[101](#_ENREF_101)] Rosso et al (2000) | *National Collaborative Perinatal Project* (Birth cohort) | Chart-review-based DSM-IV | Standardised psychological and neurological examinations | Race, gender, parental education level, parental SES, age at time of examination, obstetric variables |
| [[102](#_ENREF_102)] Rosso et al (2000) | *Helsinki 1955 Birth Cohort* (Nested case-control) | SCID (DSM-III-R) | Prenatal clinic and obstetric hospital records | Age, gender, and SES |
| [[103](#_ENREF_103)] Sacker et al (1995) | *National Child Development Study* (Birth cohort) | PSE diagnoses derived from hospital case notes | British 1958 Perinatal Mortality Survey |  |

| **Study citation** | **Cohort / Sample**  **(Study design)** | **Psychiatric outcome measure** | **Antecedent/risk factor measure** | **Analyses adjusted for the following variables:** |
| --- | --- | --- | --- | --- |
| [[104](#_ENREF_104)] Schaefer et al (2000) | *Prenatal Determinants of Schizophrenia Study* (Birth cohort) | DIGS or chart review (DSM-IV) | Child Health and Development Study interview data | Parity, maternal age, maternal ethnicity, cigarette smoking during pregnancy, gender of offspring, age at onset of disorder |
| [[105](#_ENREF_105)] Schiffman et al (2002) | *Danish High-Risk Study* (High-Risk) | SCID; PSE; psychiatric hospital records (DSM-III-R) | Assessment by paediatric neurologist |  |
| [[106](#_ENREF_106)] Schiffman et al (2002) | *Danish High-Risk Study* (High-Risk) | PSE; Current and Past Psychopathology Scales | Perception of relationship with Mother and Perception of Relationship with Father Scales |  |
| [[107](#_ENREF_107)] Schiffman et al (2004) | *Danish High-Risk Study* (High-Risk) | SCID-II; PSE (psychosis section); Register diagnoses | Sociability scale ratings based on videotaped social interactions |  |
| [[108](#_ENREF_108)] Schiffman et al (2005) | *Danish High-Risk Study* (High-Risk) | SCID; PSE psychosis section (DSM-III-R); Register diagnoses | Examination by paediatric neurologist based on standardised assessments | Parental psychiatric status, parent with schizophrenia |
| [[109](#_ENREF_109)] Schiffman et al (2006) | *Danish High-Risk Study* (High-Risk) | SCID; PSE psychosis section (DSM-III-R); Register diagnoses | Eye exam performed by paediatric neurologist |  |
| [[110](#_ENREF_110)] Schiffman et al (2009) | *Danish High-Risk Study* (High-Risk) | SCID; PSE psychosis section (DSM-III-R); Register diagnoses | Neurological examination by child neurologist | Parental age, marital status, social class and sex |
| [[111](#_ENREF_111)] Schulz et al (2014) | *National Child Development Study* (Birth cohort) | Hospital case note diagnoses (DSM-IV) | Southgate Reading Test, Problem Arithmetic Test | Paternal education, multiple birth, BSAG total score at age 7/11 years, relation with natural parents at age 7/11 years |
| [[112](#_ENREF_112)] Seidman et al (2013) | *National Collaborative Perinatal Project* (Nested case-control) | SCID (DSM-IV) | WISC; Wide Range Achievement Test measures of reading, spelling and arithmetic; tests of auditory-vocal associations, visual-motor integration and tactile form recognition |  |

| **Study citation** | **Cohort / Sample**  **(Study design)** | **Psychiatric outcome measure** | **Antecedent/risk factor measure** | **Analyses adjusted for the following variables:** |
| --- | --- | --- | --- | --- |
| [[113](#_ENREF_113)] Sipos et al (2004) | *Swedish 1973-1980 Birth Cohort* (Birth cohort) | Swedish inpatient discharge and cause of death and emigration registers | Population and housing census | Birth weight, birth length, gestational age, place of birth, season, Apgar score at 1 and 5 minutes, parity, multiple birth, highest annual income of either parent, highest SES of either parent, highest educational level of either parent |
| [[114](#_ENREF_114)] Sorensen et al (2006) | *Copenhagen High-Risk Study* (High-Risk) | PSE, PSE Etiology scale, PSE Syndrome Checklist, Current and Past Psychopathology Scales | WISC |  |
| [[115](#_ENREF_115)] Sorensen et al (2009) | *Copenhagen Perinatal Cohort* (Birth cohort) | Danish Psychiatric Central Research Register | Interview with mother 5 days after delivery | Parental SES, maternal schizophrenia, second-trimester exposure to analgesics, third-trimester exposure to diuretics, hypertension during pregnancy, offspring of single mother, breastfed ≤2 weeks  Exposure to analgesics during pregnancy and SES included in final model |
| [[116](#_ENREF_116)] Sorensen et al (2010) | *Danish High-Risk Study* (High-Risk) | SCID-II; PSE (psychosis section);Register diagnoses | WISC |  |
| [[117](#_ENREF_117)] Stringaris et al (2009) | *New York population-based study* (Population cohort) | SCID (DSM-IV) | Parent-rated DISC; parent responses to Disorganised Poverty Index (DIPOV) | Age, sex, family SES, emotional and behavioural disorders in adolescence |
| [[118](#_ENREF_118)] Suvisaari et al (2012) | *Helsinki High-Risk Study* (High-Risk) | FHDR; OPCRIT; Major Symptoms of Schizophrenia Scale | Obstetric records from public hospitals providing obstetric care | Sex, birth weight, maternal infections during pregnancy, maternal hypertension during pregnancy, and placental abnormalities |
| [[119](#_ENREF_119)] Talati et al (2013) | *Child Health and Development Study* (Nested case-control) | SCID/DIGS | Maternal interview during pregnancy | Maternal race, maternal alcohol use during pregnancy, maternal daily caffeine use during pregnancy, maternal lifetime psychopathology, maternal lifetime severe psychopathology, offspring birth weight  Matched controls on date of birth, sex, membership in the cohort at the time of illness onset, availability of maternal archived sera |
| **Study citation** | **Cohort / Sample**  **(Study design)** | **Psychiatric outcome measure** | **Antecedent/risk factor measure** | **Analyses adjusted for the following variables:** |
|  |  |  |  |  |
| [[120](#_ENREF_120)] Talovic et al (1980) | *Danish High-Risk Study* (High-Risk) | PSE, CATEGO program, and CAPPS | Maternal mental hospital records |  |
| [[121](#_ENREF_121)] Tuovinen et al (2012) | *Helsinki Birth Cohort* (Birth cohort) | FHDR; FCDR | Maternal blood pressure and urinary protein tests during pregnancy | Sex, childhood SES (paternal occupation), gestational age, parity, year of birth, birth weight, mother’s height, weight, age and BMI |
| [[122](#_ENREF_122)] Ullman et al (2012) | *Israeli population-based study* (Population cohort) | National Psychiatric Hospitalization Case Registry | *Behavioural problems* - Teacher ratings  *Academic performance* – Jerusalem school grade archive | Age, sex, school |
| [[123](#_ENREF_123)] Walker et al (1981) | *Danish High-Risk Project* (High-Risk) | CAPPS, PSE, clinical interview | Social history interviews with parents conducted by social worker; official records | IQ, mean age at follow-up, SES, paternal education |
| [[124](#_ENREF_124)] Welham et al (2009) | *Mater-University Study of Pregnancy* (Birth cohort) | CIDI or questionnaire item ‘Have you ever been told by a doctor that you have schizophrenia?’ | *Cannabis use* - Self-report  *Behavioural problems* – CBCL (mother rated) |  |
| [[125](#_ENREF_125)] Werner et al (2007) | *Jerusalem Perinatal Study* (Birth cohort) | Israeli National Psychiatric Case Registry (ICD-9) | Scale ranking 220 occupations in Israel | Year of birth  For paternal occupational prestige, adjusted for: Sex, year of birth, age of father at time of birth, father’s ethnicity |
| [[126](#_ENREF_126)] Wicks et al (2005) | *Swedish birth cohort* (Birth Cohort) | National Hospital Discharge Register (ICD-9/10) | Swedish Population and Housing Census | Gender, age, urbanicity, foreign-born parents, paternal age, and parental inpatient care for psychosis and alcohol/drug abuse |
| [[127](#_ENREF_127)] Zornberg et al (2000) | *National Collaborative Perinatal Project* (Birth cohort) | DIS-III (DSM-IV) | Neonatal neurological examination | Familial risk, gender, age, race, prenatal care, SES |

**References to Supplementary Table 5:**

1. Amminger GP, Pape S, Rock D, Roberts SA, Ott SL, Squires-Wheeler E, Kestenbaum C, Erlenmeyer-Kimling L: **Relationship between childhood behavioral disturbance and later schizophrenia in the New York High-Risk Project**. *American Journal of Psychiatry* 1999, **156**(4):525-530.

2. Arseneault L, Cannon M, Poulton R, Murray R, Caspi A, Moffitt TE: **Cannabis use in adolescence and risk for adult psychosis: longitudinal prospective study**. *British Medical Journal* 2002, **325**(7374):1212-1213.

3. Babulas V, Factor-Litvak P, Goetz R, Schaefer CA, Brown AS: **Prenatal exposure to maternal genital and reproductive infections and adult schizophrenia**. *American Journal of Psychiatry* 2006, **163**(5):927-929.

4. Bain M, Juszczak E, McInneny K, Kendell RE: **Obstetric complications and affective psychoses. Two case-control studies based on structured obstetric records**. *British Journal of Psychiatry* 2000, **176**:523-526.

5. Bao Y, Ibram G, Blaner WS, Quesenberry CP, Shen L, McKeague IW, Schaefer CA, Susser ES, Brown AS: **Low maternal retinol as a risk factor for schizophrenia in adult offspring**. *Schizophrenia Research* 2012, **137**(1-3):159-165.

6. Bearden CE, Rosso IM, Hollister JM, Sanchez LE, Hadley T, Cannon TD: **A prospective cohort study of childhood behavioral deviance and language abnormalities as predictors of adult schizophrenia**. *Schizophr Bull* 2000, **26**(2):395-410.

7. Bresnahan M, Begg MD, Brown A, Schaefer C, Sohler N, Insel B, Vella L, Susser E: **Race and risk of schizophrenia in a US birth cohort: another example of health disparity?** *International Journal of Epidemiology* 2007, **36**(4):751-758.

8. Brown AS, Schaefer CA, Wyatt RJ, Goetz R, Begg MD, Gorman JM, Susser ES: **Maternal exposure to respiratory infections and adult schizophrenia spectrum disorders: a prospective birth cohort study**. *Schizophr Bull* 2000, **26**(2):287-295.

9. Brown AS, Begg MD, Gravenstein S, Schaefer CA, Wyatt RJ, Bresnahan M, Babulas VP, Susser ES: **Serologic evidence of prenatal influenza in the etiology of schizophrenia**. *Archives of General Psychiatry* 2004, **61**(8):774-780.

10. Brown AS, Hooton J, Schaefer CA, Zhang H, Petkova E, Babulas V, Perrin M, Gorman JM, Susser ES: **Elevated maternal interleukin-8 levels and risk of schizophrenia in adult offspring**. *American Journal of Psychiatry* 2004, **161**(5):889-895.

11. Brown AS, Schaefer CA, Quesenberry CP, Jr., Liu L, Babulas VP, Susser ES: **Maternal exposure to toxoplasmosis and risk of schizophrenia in adult offspring**. *American Journal of Psychiatry* 2005, **162**(4):767-773.

12. Brown AS, Schaefer CA, Quesenberry CP, Jr., Shen L, Susser ES: **No evidence of relation between maternal exposure to herpes simplex virus type 2 and risk of schizophrenia?** *American Journal of Psychiatry* 2006, **163**(12):2178-2180.

13. Brown AS, Schaefer CA, Wyatt RJ, Begg MD, Goetz R, Bresnahan MA, Harkavy-Friedman J, Gorman JM, Malaspina D, Susser ES: **Paternal age and risk of schizophrenia in adult offspring**. *American Journal of Psychiatry* 2006, **159**(9):1528-1533.

14. Buizer-Voskamp JE, Laan W, Staal WG, Hennekam EAM, Aukes MF, Termorshuizen F, Kahn RS, Boks MPM, Ophoff RA: **Paternal age and psychiatric disorders: Findings from a Dutch population registry**. *Schizophrenia Research* 2011, **129**(2–3):128-132.

15. Buka SL, Cannon TD, Torrey EF, Yolken RH: **Maternal Exposure to Herpes Simplex Virus and Risk of Psychosis Among Adult Offspring**. *Biological Psychiatry* 2008, **63**(8):809-815.

16. Burman B, Mednick SA, Machon RA, Parnas J, Schulsinger F: **Children at high risk for schizophrenia: parent and offspring perceptions of family relationships**. *Journal of Abnormal Psychology* 1987, **96**(4):364-366.

17. Canetta S, Sourander A, Surcel HM, Hinkka-Yli-Salomaki S, Leiviska J, Kellendonk C, McKeague IW, Brown AS: **Elevated Maternal C-Reactive Protein and Increased Risk of Schizophrenia in a National Birth Cohort**. *Am J Psychiatry* in press.

18. Canetta SE, Bao Y, Co MDT, Ennis FA, Cruz J, Terajima M, Shen L, Kellendonk C, Schaefer CA, Brown AS: **Serological documentation of maternal influenza exposure and bipolar disorder in adult offspring**. *American Journal of Psychiatry* 2014, **171**(5):557-563.

19. Cannon M, Jones P, Murray RM, Wadsworth ME: **Childhood laterality and later risk of schizophrenia in the 1946 British birth cohort**. *Schizophrenia Research* 1997, **26**(2-3):117-120.

20. Cannon M, Caspi A, Moffitt TE, Harrington H, Taylor A, Murray RM, Poulton R: **Evidence for early-childhood, pan-developmental impairment specific to schizophreniform disorder: results from a longitudinal birth cohort**. *Archives of General Psychiatry* 2002, **59**(5):449-456.

21. Cannon TD, Bearden CE, Hollister JM, Rosso IM, Sanchez LE, Hadley T: **Childhood Cognitive Functioning in Schizophrenia Patients and Their Unaffected Siblings: A Prospective Cohort Study**. *Schizophr Bull* 2000, **26**(2):379-393.

22. Cantor-Graae E, McNeil TF, Sjöström K, Nordström LG, Rosenlund T: **Maternal demographic correlates of increased history of obstetric complications in schizophrenia**. *Journal of Psychiatric Research* 1997, **31**(3):347-357.

23. Carlson GA, Weintraub S: **Childhood behavior problems and bipolar disorder - relationship or coincidence?** *Journal of Affective Disorders* 1993, **28**(3):143-153.

24. Carter JW, Schulsinger F, Parnas J, Cannon T, Mednick SA: **A multivariate prediction model of schizophrenia**. *Schizophr Bull* 2003, **28**(4):649-682.

25. Castle DJ, Scott K, Wessely S, Murray RM: **Does social deprivation during gestation and early life predispose to later schizophrenia?** *Soc Psychiatry Psychiatr Epidemiol* 1993, **28**(1):1-4.

26. Chong S, Subramaniam M, Lee IM, Pek E, Cheok C, Verma S, Wong J: **Academic attainment: a predictor of psychiatric disorders?** *Social Psychiatry and Psychiatric Epidemiology* 2009, **44**(11):999-1004.

27. Clarke MC, Tanskanen A, Huttunen M, Whittaker JC, Cannon M: **Evidence for an Interaction Between Familial Liability and Prenatal Exposure to Infection in the Causation of Schizophrenia**. *American Journal of Psychiatry* 2009, **166**(9):1025-1030.

28. Clarke MC, Tanskanen A, Huttunen M, Leon DA, Murray RM, Jones PB, Cannon M: **Increased risk of schizophrenia from additive interaction between infant motor developmental delay and obstetric complications: evidence from a population-based longitudinal study.[Erratum appears in Am J Psychiatry. 2011, 168(12):1345]**. *American Journal of Psychiatry* 2011, **168**(12):1295-1302.

29. Corcoran C, Perrin M, Harlap S, Deutsch L, Fennig S, Manor O, Nahon D, Kimhy D, Malaspina D, Susser E: **Effect of socioeconomic status and parents’ education at birth on risk of schizophrenia in offspring**. *Social Psychiatry and Psychiatric Epidemiology* 2009, **44**(4):265-271.

30. Corcoran C, Perrin M, Harlap S, Deutsch L, Fennig S, Manor O, Nahon D, Kimhy D, Malaspina D, Susser E: **Incidence of schizophrenia among second-generation immigrants in the jerusalem perinatal cohort**. *Schizophr Bull* 2009, **35**(3):596-602.

31. Cornblatt BA, Obuchowski M, Roberts S, Pollack S, Erlenmeyer–Kimling L: **Cognitive and behavioral precursors of schizophrenia**. *Development and Psychopathology* 1999, **11**(03):487-508.

32. Crow TJ, Done DJ, Sacker A: **Chidhood precursors of psychiosis as clues to its evolutionary orgins**. *European Archives of Psychiatry and Clinical Neuroscience* 1995, **245**(2):61-69.

33. Dalman C, Allebeck P, Gunnell D, Harrison G, Kristensson K, Lewis G, Lofving S, Rasmussen F, Wicks S, Karlsson H: **Infections in the CNS during childhood and the risk of subsequent psychotic illness: a cohort study of more than one million Swedish subjects**. *American Journal of Psychiatry* 2008, **165**(1):59-65.

34. Done DJ, Crow TJ, Johnstone EC, Sacker A: **Childhood antecedents of schizophrenia and affective illness: Social adjustment at ages 7 and 11**. *British Medical Journal* 1994, **309**(6956):699.

35. Ekstrom M, Sorensen H, Mednick SA: **Premorbid personality in schizophrenia spectrum: A prospective study**. *Nordic Journal of Psychiatry* 2006, **60**(5):417-422.

36. Erlenmeyer-Kimling L, Rock D, Roberts SA, Janal M, Kestenbaum C, Cornblatt B, Adamo UH, Gottesman II: **Attention, memory, and motor skills as childhood predictors of schizophrenia-related psychoses: the New York High-Risk Project**. *American Journal of Psychiatry* 2000, **157**(9):1416-1422.

37. Fisher HL, Caspi A, Poulton R, Meier MH, Houts R, Harrington H, Arseneault L, Moffitt TE: **Specificity of childhood psychotic symptoms for predicting schizophrenia by 38 years of age: a birth cohort study**. *Psychol Med* 2013, **43**(10):2077-2086.

38. Freedman D, Bao Y, Kremen WS, Vinogradov S, McKeague IW, Brown AS: **Birth weight and neurocognition in schizophrenia spectrum disorders**. *Schizophr Bull* 2013, **39**(3):592-600.

39. Goldstein MJ: **The UCLA High-Risk Project**. *Schizophr Bull* 1987, **13**(3):505-514.

40. Griffith JJ, Mednick SA, Schulsinger F, Diderichsen B: **Verbal associative disturbances in children at high risk for schizophrenia**. *Journal of Abnormal Psychology* 1980, **89**(2):125-131.

41. Gunther-Genta F, Bovet P, Hohlfeld P: **Obstetric complications and schizophrenia. A case-control study**. *British Journal of Psychiatry* 1994, **164**(2):165-170.

42. Hare EH, Price JS, Slater E: **Parental social class in psychiatric patients**. *Br J Psychiatry* 1972, **121**(564):515-534.

43. Harper KN, Hibbeln JR, Deckelbaum R, Quesenberry CP, Jr., Schaefer CA, Brown AS: **Maternal serum docosahexaenoic acid and schizophrenia spectrum disorders in adult offspring**. *Schizophrenia Research* 2011, **128**(1-3):30-36.

44. Harrison G, Gunnell D, Glazebrook C, Page K, Kwiecinski R: **Association between schizophrenia and social inequality at birth: case-control study**. *Br J Psychiatry* 2001, **179**:346-350.

45. Haukka JK, Suvisaari J, Lonnqvist J: **Family structure and risk factors for schizophrenia: case-sibling study**. *BMC Psychiatry* 2004, **4**:41.

46. Herman DB, Brown AS, Opler MG, Desai M, Malaspina D, Bresnahan M, Schaefer CA, Susser ES: **Does unwantedness of pregnancy predict schizophrenia in the offspring? Findings from a prospective birth cohort study**. *Social Psychiatry and Psychiatric Epidemiology* 2006, **41**(8):605-610.

47. Hollister J, Laing P, Mednick SA: **Rhesus incompatibility as a risk factor for schizophrenia in male adults**. *Archives of General Psychiatry* 1996, **53**(1):19-24.

48. Hultman CM, Ohman A, Cnattingius S, Wieselgren IM, Lindström LH: **Prenatal and neonatal risk factors for schizophrenia**. *British Journal of Psychiatry* 1997, **170**(2):128-133.

49. Hultman CM, Sparen P, Takei N, Murray RM, Cnattingius S: **Prenatal and perinatal risk factors for schizophrenia, affective psychosis, and reactive psychosis of early onset: Case-control study**. *British Medical Journal* 1999, **318**(7181):421-426.

50. Isohanni I, Jarvelin M-R, Nieminen P, Jones P, Rantakallio P, Jokelainen J, Isohanni M: **School performance as a predictor of psychiatric hospitalization in adult life. A 28-year follow-up in the Northern Finland 1966 Birth Cohort**. *Psychol Med* 1998, **28**(04):967-974.

51. Isohanni M, Jones PB, Moilanen K, Rantakallio P, Veijola J, Oja H, Koiranen M, Jokelainen J, Croudace T, Järvelin MR: **Early developmental milestones in adult schizophrenia and other psychoses. A 31-year follow-up of the Northern Finland 1966 Birth Cohort**. *Schizophrenia Research* 2001, **52**(1–2):1-19.

52. Jones P, Murray R, Rodgers B, Marmot M: **Child developmental risk factors for adult schizophrenia in the British 1946 birth cohort**. *The Lancet* 1994, **344**(8934):1398-1402.

53. Jones PB, Rantakallio P, Hartikainen AL, Isohanni M, Sipila P: **Schizophrenia as a long-term outcome of pregnancy, delivery, and perinatal complications: a 28-year follow-up of the 1966 north Finland general population birth cohort**. *American Journal of Psychiatry* 1998, **155**(3):355-364.

54. Kawai M, Minabe Y, Takagai S, Ogai M, Matsumoto H, Mori N, Takei N: **Poor maternal care and high maternal body mass index in pregnancy as a risk factor for schizophrenia in offspring**. *Acta Psychiatrica Scandinavica* 2004, **110**(4):257-263.

55. Kemppainen L, Makikyro T, Jokelainen J, Nieminen P, Jarvelin MR, Isohanni M: **Is grand multiparity associated with offsprings' hospital-treated mental disorders? A 28-year follow-up of the North Finland 1966 birth cohort**. *Social Psychiatry and Psychiatric Epidemiology* 2000, **35**(3):104-108.

56. Kendell RE, McInneny K, Juszczak E, Bain M: **Obstetric complications and schizophrenia. Two case-control studies based on structured obstetric records**. *British Journal of Psychiatry* 2000, **176**:516-522.

57. Khashan AS, Abel KM, McNamee R, et al.: **Higher risk of offspring schizophrenia following antenatal maternal exposure to severe adverse life events**. *Archives of General Psychiatry* 2008, **65**(2):146-152.

58. Kim-Cohen J, Caspi A, Moffitt TE, Harrington H, Milne BJ, Poulton R: **Prior juvenile diagnoses in adults with mental disorder: Developmental follow-back of a prospective-longitudinal cohort**. *Archives of General Psychiatry* 2003, **60**(7):709-717.

59. Koenen KC, Moffitt TE, Roberts AL, Martin LT, Kubzansky L, Harrington H, Poulton R, Caspi A: **Childhood IQ and adult mental disorders: a test of the cognitive reserve hypothesis**. *American Journal of Psychiatry* 2009, **166**(1):50-57.

60. Koponen H, Rantakallio P, Veijola J, Jones P, Jokelainen J, Isohanni M: **Childhood central nervous system infections and risk for schizophrenia**. *European Archives of Psychiatry and Clinical Neuroscience* 2004, **254**(1):9-13.

61. Kremen WS, Vinogradov S, Poole JH, Schaefer CA, Deicken RF, Factor-Litvak P, Brown AS: **Cognitive decline in schizophrenia from childhood to midlife: a 33-year longitudinal birth cohort study**. *Schizophrenia Research* 2010, **118**(1-3):1-5.

62. Laursen TM, Munk-Olsen T, Nordentoft M, Bo Mortensen P: **A comparison of selected risk factors for unipolar depressive disorder, bipolar affective disorder, schizoaffective disorder, and schizophrenia from a danish population-based cohort**. *Journal of Clinical Psychiatry* 2007, **68**(11):1673-1681.

63. Leask SJ, Done DJ, Crow TJ: **Adult psychosis, common childhood infections and neurological soft signs in a national birth cohort**. *British Journal of Psychiatry* 2002, **181**:387-392.

64. MacCabe JH, Wicks S, Lofving S, David AS, Berndtsson A, Gustafsson JE, Allebeck P, Dalman C: **Decline in cognitive performance between ages 13 and 18 years and the risk for psychosis in adulthood: a Swedish longitudinal cohort study in males**. *JAMA Psychiatry* 2013, **70**(3):261-270.

65. Machon RA, Mednick SA, Schulsinger F: **Seasonality, birth complications and schizophrenia in a high risk sample**. *British Journal of Psychiatry* 1987, **151**:122-124.

66. Maki P, Riekki T, Miettunen J, Isohanni M, Jones PB, Murray GK, Veijola J: **Schizophrenia in the offspring of antenatally depressed mothers in the northern Finland 1966 birth cohort: relationship to family history of psychosis**. *American Journal of Psychiatry* 2010, **167**(1):70-77.

67. Makikyro T, Isohanni M, Moring J, Oja H, Hakko H, Jones P, Rantakallio P: **Is a child's risk of early onset schizophrenia increased in the highest social class?** *Schizophrenia Research* 1997, **23**(3):245-252.

68. Marcelis M, Navarro-Mateu F, Murray R, Selten J-P, Van Os J: **Urbanization and psychosis: a study of 1942–1978 birth cohorts in The Netherlands**. *Psychol Med* 1998, **28**(04):871-879.

69. Mathiasen R, Hansen BM, Forman JL, Kessing LV, Greisen G: **The risk of psychiatric disorders in individuals born prematurely in Denmark from 1974 to 1996**. *Acta Paediatrica* 2011, **100**(5):691-699.

70. McGrath J, Saari K, Hakko H, Jokelainen J, Jones P, Jarvelin M-R, Chant D, Isohanni M: **Vitamin D supplementation during the first year of life and risk of schizophrenia: a Finnish birth cohort study**. *Schizophrenia Research* 2004, **67**(2-3):237-245.

71. McGrath JJ, Eyles DW, Pedersen CB, Anderson C, Ko P, Burne TH, Norgaard-Pedersen B, Hougaard DM, Mortensen PB: **Neonatal vitamin D status and risk of schizophrenia: a population-based case-control study**. *Arch Gen Psychiatry* 2010, **67**(9):889-894.

72. Menezes PR, Lewis G, Rasmussen F, Zammit S, Sipos A, Harrison GL, Tynelius P, Gunnell D: **Paternal and maternal ages at conception and risk of bipolar affective disorder in their offspring**. *Psychol Med* 2010, **40**(03):477-485.

73. Meyer SE, Carlson GA, Wiggs EA, Martinez PE, Ronsaville DS, Klimes-dougan B, Gold PW, Radke-yarrow M: **A prospective study of the association among impaired executive functioning, childhood attentional problems, and the development of bipolar disorder**. *Development and Psychopathology* 2004, **16**(2):461-476.

74. Meyer SE, Carlson GA, Youngstrom E, Ronsaville DS, Martinez PE, Gold PW, Hakak R, Radke-Yarrow M: **Long-term outcomes of youth who manifested the CBCL-Pediatric Bipolar Disorder phenotype during childhood and/or adolescence**. *Journal of Affective Disorders* 2009, **113**(3):227-235.

75. Moilanen K, Jokelainen J, Jones PB, Hartikainen A-L, Jarvelin M-R, Isohanni M: **Deviant intrauterine growth and risk of schizophrenia: a 34-year follow-up of the Northern Finland 1966 Birth Cohort**. *Schizophrenia Research* 2010, **124**(1-3):223-230.

76. Monfils GW, Josefsson A, Ekholm Selling K, Sydsjö G: **Preterm birth or foetal growth impairment and psychiatric hospitalization in adolescence and early adulthood in a Swedish population-based birth cohort**. *Acta Psychiatrica Scandinavica* 2009, **119**(1):54-61.

77. Mortensen PB, Pedersen CB, Hougaard DM, Nørgaard-Petersen B, Mors O, Børglum AD, Yolken RH: **A Danish National Birth Cohort study of maternal HSV-2 antibodies as a risk factor for schizophrenia in their offspring**. *Schizophrenia Research* 2010, **122**(1–3):257-263.

78. Mouridsen SE, Hauschild K-M: **A longitudinal study of schizophrenia- and affective spectrum disorders in individuals diagnosed with a developmental language disorder as children**. *Journal of Neural Transmission* 2008, **115**(11):1591-1597.

79. Mulvany F, O'Callaghan E, Takei N, Byrne M, Fearon P, Larkin C: **Effect of social class at birth on risk and presentation of schizophrenia: case-control study**. *BMJ* 2001, **323**(7326):1398-1401.

80. Nielsen PR, Laursen TM, Mortensen PB: **Association Between Parental Hospital-Treated Infection and the Risk of Schizophrenia in Adolescence and Early Adulthood**. *Schizophr Bull* 2013, **39**(1):230-237.

81. Niemi LT, Suvisaari JM, Haukka JK, Lonnqvist JK: **Do maternal psychotic symptoms predict offspring's psychotic disorder? Findings from the Helsinki High-Risk Study**. *Psychiatry Research* 2004, **125**(2):105-115.

82. Niemi LT, Suvisaari JM, Haukka JK, Lonnqvist JK: **Childhood predictors of future psychiatric morbidity in offspring of mothers with psychotic disorder: results from the Helsinki High-Risk Study**. *British Journal of Psychiatry* 2005, **186**:108-114.

83. Niemi LT, Suvisaari JM, Haukka JK, Lonnqvist JK: **Childhood growth and future development of psychotic disorder among Helsinki high-risk children**. *Schizophrenia Research* 2005, **76**(1):105-112.

84. Niendam TA, Bearden CE, Rosso IM, Sanchez LE, Hadley T, Nuechterlein KH, Cannon TD: **A prospective study of childhood neurocognitive functioning in schizophrenic patients and their siblings**. *American Journal of Psychiatry* 2003, **160**(11):2060-2062.

85. Nosarti C, Reichenberg A, Murray RM, et al.: **Preterm birth and psychiatric disorders in young adult life**. *Archives of General Psychiatry* 2012, **69**(6):610-617.

86. Olin SS, John RS, Mednick SA: **Assessing the predictive value of teacher reports in a high risk sample for schizophrenia: a ROC analysis**. *Schizophrenia Research* 1998, **16**(1):53-66.

87. Orlovska S, Pedersen MS, Benros ME, Mortensen PB, Agerbo E, Nordentoft M: **Head injury as risk factor for psychiatric disorders: a nationwide register-based follow-up study of 113,906 persons with head injury**. *American Journal of Psychiatry* 2014, **171**(4):463-469.

88. Osler M, Lawlor DA, Nordentoft M: **Cognitive function in childhood and early adulthood and hospital admission for schizophrenia and bipolar disorders in Danish men born in 1953**. *Schizophrenia Research* 2007, **92**(1–3):132-141.

89. Ott SL, Spinelli S, Rock D, Roberts S, Amminger GP, Erlenmeyer-Kimling L: **The New York High-Risk Project: social and general intelligence in children at risk for schizophrenia**. *Schizophrenia Research* 1998, **31**(1):1-11.

90. Ott SL, Roberts S, Rock D, Allen J, Erlenmeyer-Kimling L: **Positive and negative thought disorder and psychopathology in childhood among subjects with adulthood schizophrenia**. *Schizophrenia Research* 2002, **58**(2-3):231-239.

91. Parboosing R, Bao Y, Shen L, Schaefer CA, Brown AS: **Gestational influenza and bipolar disorder in adult offspring**. *JAMA Psychiatry* 2013, **70**(7):677-685.

92. Parnas J, Schulsinger F, Teasdale TW, Schulsinger H, Feldman PM, Mednick SA: **Perinatal complications and clinical outcome within the schizophrenia spectrum**. *British Journal of Psychiatry* 1982, **140**:416-420.

93. Parnas J, Jorgensen A: **Pre-morbid psychopathology in schizophrenia spectrum**. *British Journal of Psychiatry* 1989, **155**:623-627.

94. Perrin M, Harlap S, Kleinhaus K, Lichtenberg P, Manor O, Draiman B, Fennig S, Malaspina D: **Older paternal age strongly increases the morbidity for schizophrenia in sisters of affected females**. *American Journal of Medical Genetics Part B, Neuropsychiatric Genetics* 2010, **153B**(7):1329-1335.

95. Perrin MA, Chen H, Sandberg DE, Malaspina D, Brown AS: **Growth trajectory during early life and risk of adult schizophrenia**. *British Journal of Psychiatry* 2007, **191**(6):512-520.

96. Poulton R, Caspi A, Moffitt TE, Cannon M, Murray R, Harrington H: **Children's self-reported psychotic symptoms and adult schizophreniform disorder: A 15-year longitudinal study**. *Archives of General Psychiatry* 2000, **57**(11):1053-1058.

97. Preti A, Cardascia L, Zen T, Marchetti M, Favaretto G, Miotto P: **Risk for obstetric complications and schizophrenia**. *Psychiatry Research* 2000, **96**(2):127-139.

98. Rantakallio P, Jones P, Moring J, Von Wendt L: **Association between central nervous system infections during childhood and adult onset schizophrenia and other psychoses: a 28-year follow-up**. *International Journal of Epidemiology* 1997, **26**(4):837-843.

99. Reichart CG, van der Ende J, Wals M, Hillegers MHJ, Nolen WA, Ormel J, Verhulst FC: **The use of the GBI as predictor of bipolar disorder in a population of adolescent offspring of parents with a bipolar disorder**. *Journal of Affective Disorders* 2005, **89**(1-3):147-155.

100. Riordan DV, Morris C, Hattie J, Stark C: **Interbirth spacing and offspring mental health outcomes**. *Psychol Med* 2012, **42**(12):2511-2521.

101. Rosso IM, Bearden CE, Hollister JM, Gasperoni TL, Sanchez LE, Hadley T, Cannon TD: **Childhood neuromotor dysfunction in schizophrenia patients and their unaffected siblings: a prospective cohort study**. *Schizophr Bull* 2000, **26**(2):367-378.

102. Rosso IM, Cannon TD, Huttunen T, Huttunen MO, Lonnqvist J, Gasperoni TL: **Obstetric risk factors for early-onset schizophrenia in a Finnish birth cohort**. *American Journal of Psychiatry* 2000, **157**(5):801-807.

103. Sacker A, Done DJ, Crow TJ, Golding J: **Antecedents of schizophrenia and affective illness. Obstetric complications**. *British Journal of Psychiatry* 1995, **166**(6):734-741.

104. Schaefer CA, Brown AS, Wyatt RJ, Kline J, Begg MD, Bresnahan MA, Susser ES: **Maternal Prepregnant Body Mass and Risk of Schizophrenia in Adult Offspring**. *Schizophr Bull* 2000, **26**(2):275-286.

105. Schiffman J, Ekstrom M, LaBrie J, Schulsinger F, Sorensen H, Mednick S: **Minor physical anomalies and schizophrenia spectrum disorders: a prospective investigation**. *American Journal of Psychiatry* 2002, **159**(2):238-243.

106. Schiffman J, LaBrie J, Carter J, Cannon T, Schulsinger F, Parnas J, Mednick S: **Perception of parent-child relationships in high-risk families, and adult schizophrenia outcome of offspring**. *Journal of Psychiatric Research* 2002, **36**(1):41-47.

107. Schiffman J, Walker E, Ekstrom M, Schulsinger F, Sorensen H, Mednick S: **Childhood videotaped social and neuromotor precursors of schizophrenia: a prospective investigation**. *American Journal of Psychiatry* 2004, **161**(11):2021-2027.

108. Schiffman J, Pestle S, Mednick S, Ekstrom M, Sorensen H, Mednick S: **Childhood laterality and adult schizophrenia spectrum disorders: a prospective investigation**. *Schizophrenia Research* 2005, **72**(2-3):151-160.

109. Schiffman J, Maeda JA, Hayashi K, Michelsen N, Sorensen HJ, Ekstrom M, Abe KA, Chronicle EP, Mednick SA: **Premorbid childhood ocular alignment abnormalities and adult schizophrenia-spectrum disorder**. *Schizophrenia Research* 2006, **81**(2-3):253-260.

110. Schiffman J, Sorensen HJ, Maeda J, Mortensen EL, Victoroff J, Hayashi K, Michelsen NM, Ekstrom M, Mednick S: **Childhood motor coordination and adult schizophrenia spectrum disorders**. *American Journal of Psychiatry* 2009, **166**(9):1041-1047.

111. Schulz J, Sundin J, Leask S, Done DJ: **Risk of adult schizophrenia and its relationship to childhood IQ in the 1958 British birth cohort**. *Schizophr Bull* 2014, **40**(1):143-151.

112. Seidman LJ, Cherkerzian S, Goldstein JM, Agnew-Blais J, Tsuang MT, Buka SL: **Neuropsychological performance and family history in children at age 7 who develop adult schizophrenia or bipolar psychosis in the New England Family Studies**. *Psychol Med* 2013, **43**(01):119-131.

113. Sipos A, Rasmussen F, Harrison G, Tynelius P, Lewis G, Leon DA, Gunnell D: **Paternal Age And Schizophrenia: A Population Based Cohort Study**. *British Medical Journal* 2004, **329**(7474):1070-1073.

114. Sorensen HJ, Mortensen EL, Parnas J, Mednick SA: **Premorbid neurocognitive functioning in schizophrenia spectrum disorder**. *Schizophr Bull* 2006, **32**(3):578-583.

115. Sørensen HJ, Mortensen EL, Reinisch JM, Mednick SA: **Association Between Prenatal Exposure to Bacterial Infection and Risk of Schizophrenia**. *Schizophr Bull* 2009, **35**(3):631-637.

116. Sørensen HJ, Mortensen EL, Schiffman J, Ekstrøm M, Denenney D, Mednick SA: **Premorbid IQ and adult schizophrenia spectrum disorder: Verbal Performance subtests**. *Psychiatry Research* 2010, **178**(1):23-26.

117. Stringaris A, Cohen P, Pine DS, Leibenluft E: **Adult Outcomes of Youth Irritability: A 20-Year Prospective Community-Based Study**. *American Journal of Psychiatry* 2009, **166**(9):1048-1054.

118. Suvisaari JM, Taxell-Lassas V, Pankakoski M, Haukka JK, Lönnqvist JK, Häkkinen LT: **Obstetric Complications as Risk Factors for Schizophrenia Spectrum Psychoses in Offspring of Mothers With Psychotic Disorder**. *Schizophr Bull* 2012, **39**(5):1056-1066.

119. Talati A, Bao Y, Kaufman J, Shen L, Schaefer CA, Brown AS: **Maternal smoking during pregnancy and bipolar disorder in offspring**. *American Journal of Psychiatry* 2013, **170**(10):1178-1185.

120. Talovic SA, Mednick SA, Schulsinger F, Falloon IR: **Schizophrenia in high-risk subjects: prognostic maternal characteristics**. *Journal of Abnormal Psychology* 1980, **89**(3):501-504.

121. Tuovinen S, Räikkönen K, Pesonen A-K, Lahti M, Heinonen K, Wahlbeck K, Kajantie E, Osmond C, Barker DJP, Eriksson JG: **Hypertensive disorders in pregnancy and risk of severe mental disorders in the offspring in adulthood: The Helsinki Birth Cohort Study**. *Journal of Psychiatric Research* 2012, **46**(3):303-310.

122. Ullman VZ, Levine SZ, Reichenberg A, Rabinowitz J: **Real-world premorbid functioning in schizophrenia and affective disorders during the early teenage years: A population-based study of school grades and teacher ratings**. *Schizophrenia Research* 2012, **136**(1–3):13-18.

123. Walker E, Hoppes E, Emory E, Mednick S, Schulsinger F: **Environmental factors related to schizophrenia in psychophysiologically labile high-risk males**. *Journal of Abnormal Psychology* 1981, **90**(4):313-320.

124. Welham J, Scott J, Williams G, Najman J, Bor W, O'Callaghan M, McGrath J: **Emotional and behavioural antecedents of young adults who screen positive for non-affective psychosis: a 21-year birth cohort study**. *Psychol Med* 2009, **39**(4):625-634.

125. Werner S, Malaspina D, Rabinowitz J: **Socioeconomic status at birth is associated with risk of schizophrenia: population-based multilevel study**. *Schizophr Bull* 2007, **33**(6):1373-1378.

126. Wicks S, Hjern A, Gunnell D, Lewis G, Dalman C: **Social adversity in childhood and the risk of developing psychosis: a national cohort study**. *Am J Psychiatry* 2005, **162**(9):1652-1657.

127. Zornberg GL, Buka SL, Tsuang MT: **Hypoxic-ischemia-related fetal/neonatal complications and risk of schizophrenia and other nonaffective psychoses: a 19-year longitudinal study**. *American Journal of Psychiatry* 2000, **157**(2):196-202.
